# Supplementary material for: Membrane-associated effluxosomes coordinate multi-metal resistance in Mycobacterium tuberculosis
Source: EMBO J. 2026 Feb 13;45(7):2306–37. doi: 10.1038/s44318-026-00715-1 (PMC13043812; doi:10.1038/s44318-026-00715-1)
Supplement: Supplementary file 1 — Appendix [file 44318_2026_715_MOESM1_ESM.pdf]

Appendix

Table of content

Page 3: Appendix Figure S1
Page 4: Appendix Figure S2

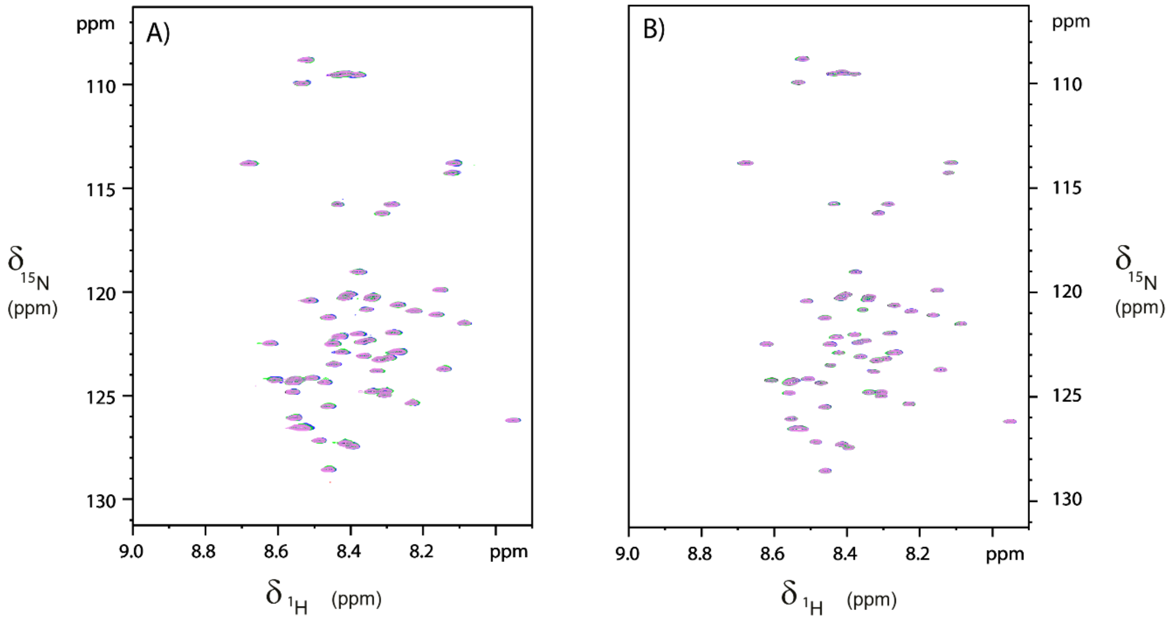

Appendix Figure S1. Titration of PacL2 by zinc and cadmium monitored by NMR. ^{1}H–^{15}N HSQC overlay spectra of 77 µM ^{15}N-labelled SolPacL2 with 0 (black), 2 (bleu), 5 (green), and 10 (light purple) equivalents of zinc (**A**) and cadmium (**B**).

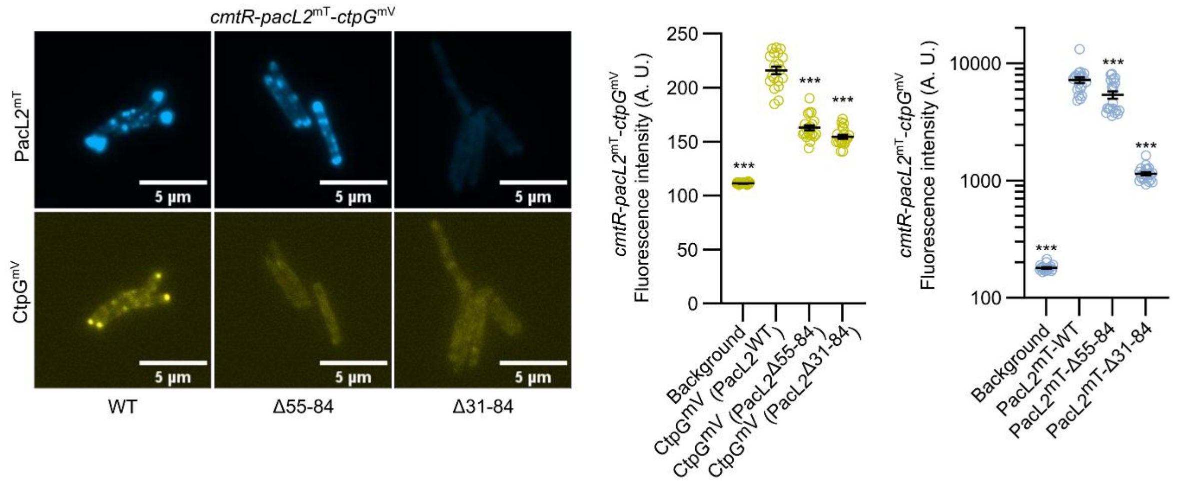

Appendix Figure S2. Epifluorescence microscopy images of live *M. smegmatis* strains carrying a genome-integrative vector expressing the indicated *M. tuberculosis* genes under the control of their native promoter, cultivated with 100 nM $CdSO_4$ (PacL2^{mT}: PacL2–mTurquoise fusion; CtpG^{mV}: CtpG–mVenus fusion; PacL1^{mT}: PacL1–mTurquoise fusion; CtpC^{mV}: CtpC–mVenus fusion). Graphs show mean fluorescence intensity (gray values) quantified in ImageJ from 8-bit grayscale images of cells of the indicated strains. Data are shown as mean ± SEM from individual cells, represented by yellow or blue dots. ***P < 0.001.
