# Supplementary material for: Membrane-associated effluxosomes coordinate multi-metal resistance in Mycobacterium tuberculosis
Source: EMBO J. 2026 Feb 13;45(7):2306–37. doi: 10.1038/s44318-026-00715-1 (PMC13043812; doi:10.1038/s44318-026-00715-1)
Supplement: Supplementary file 2 — Table EV1 [file 44318_2026_715_MOESM2_ESM.docx]

| Genes | ProteinDescriptions | Fold change (log2) | p-value (-log10) | Subcellular location* |
| --- | --- | --- | --- | --- |
| ***pacL1*** | DUF1490 family protein | 6,91 | 8,26 | Plasma membrane and cell wall |
| ***Rv1265*** | Uncharacterized protein | 4,48 | 6,06 | Plasma membrane and cell wall |
| ***ctpC*** | Metal cation transporter P-type ATPase | 3,20 | 7,33 | Plasma membrane and cell wall |
| *Rv2304c* | Uncharacterized protein | 2,47 | 4,83 | Unknown |
| ***PPE20*** | PPE family protein | 2,44 | 4,08 | Secreted |
| *Rv1066* | Rhodanese domain-containing protein | 1,69 | 1,63 | unknown |
| ***Rv2083*** | PPE family protein | 1,61 | 2,91 | Plasma membrane |
| *Rv2295* | CbrC family protein | 1,51 | 4,49 | unknown |
| *Rv0572c* | Uncharacterized protein | 1,51 | 2,38 | unknown |
| *Rv2204c* | FeS cluster biogenesis domain-containing prot. | 1,50 | 4,35 | Plasma membrane |
| *fdxA* | Ferredoxin | 1,33 | 1,75 | Cytosol |
| *Rv0680c* | Transmembrane protein | 1,29 | 3,07 | Plasma membrane |
| *Rv0784* | Deacetylase | 1,18 | 2,38 | Unknown |
| *ruvC* | Crossover junction endodeoxyribonuclease | 1,17 | 2,90 | Unknown |
| ***moeX*** | Molybdopterin biosynthesis protein | 1,11 | 1,53 | Unknown |
| *Rv2809* | Uncharacterized protein | 1,07 | 2,61 | Unknown |
| *Rv3877* | Transmembrane protein | 1,05 | 3,19 | Plasma membrane and cell wall |
| *Rv2686c* | Antibiotic ABC transporter | 1,04 | 2,58 | Plasma membrane |
| *Rv0133* | Acetyltransferase | 1,03 | 5,30 | Plasma membrane |
| *chdC* | Coproheme decarboxylase | 1,02 | 4,52 | Plasma membrane |
| *rpe* | Ribulose-phosphate 3-epimerase | 1,01 | 2,01 | Cytosol |
| ***ahpC*** | Alkyl hydroperoxide reductase subunit C | 1,01 | 4,32 | Cytosol, plasma membrane, and cell wall |
| *according to GO annotations on QuickGO | | | | |

**Table EV1. Network of PacL1^IntALFA^ interactions with *M. tuberculosis* proteins.** *M. tuberculosis* proteins significantly more biotinylated (Log2 fold-change > 1; -log10 p-value > 1.3) in a strain expressing a TurboID-nanobody fusion protein along with the PacL1 protein containing an internal ALFA tag (PacL1^Int-ALFA^), compared to a strain expressing the TurboID-nanobody fusion protein with the two first TM domains of the *E. coli* MalF protein carrying an ALFA-tag (MalF_(1,2)-_ALFA). Subcellular localizations were determined based on Gene Ontology (GO) annotations from QuickGO. Proteins highlighted in bold indicate hits also detected with the PacL1^Cter​-ALFA^ construct (see **Table 1**).
