# Supplementary material for: Membrane-associated effluxosomes coordinate multi-metal resistance in Mycobacterium tuberculosis
Source: EMBO J. 2026 Feb 13;45(7):2306–37. doi: 10.1038/s44318-026-00715-1 (PMC13043812; doi:10.1038/s44318-026-00715-1)
Supplement: Supplementary file 3 — Table EV2 [file 44318_2026_715_MOESM3_ESM.docx]

| Strains | Genetic backgrounds | References or sources |
| --- | --- | --- |
|  |  |  |
| *M. smegmatis* (MC^2^155) |  |  |
| BYM520 | ΔMSMEG_6059-6058::*dif5* ΔMSMEG0755::*dif4* | PMID: 35961955 |
|  |  |  |
| *M. tuberculosis* (H37Rv) |  |  |
| BYM-Mtb40 | WT H37Rv | This work |
| BYM-Mtb65 | ∆*pacL1-ctpC*::ZeoR | PMID: 35961955 |
| BYM-Mtb193 | ∆*pacL1*::*dif4* ∆*pacL2*::dif6 ∆*pacL3*::*dif5* | PMID: 35961955 |
| BYM-Mtb196 | ∆*pacL2-ctpG*::*dif5* | This work |
| BYM-Mtb223 | ∆*pacL1*::*dif4* ∆*pacL2*::*dif6* | This work |
| BYM-Mtb229 | ∆*pacL2*::*dif6* | This work |
| BYM-Mtb274 | *∆pacL2-ctpG*::*dif5 ∆pacL1-ctpC*::*dif4* | This work |

**Table EV2. Strains used in this work.**
