# Supplementary material for: Membrane-associated effluxosomes coordinate multi-metal resistance in Mycobacterium tuberculosis
Source: EMBO J. 2026 Feb 13;45(7):2306–37. doi: 10.1038/s44318-026-00715-1 (PMC13043812; doi:10.1038/s44318-026-00715-1)
Supplement: Supplementary file 4 — Table EV3 [file 44318_2026_715_MOESM4_ESM.docx]

| Primers name | Sequence (5'-3') |
| --- | --- |
| Construct of Δ(*pacL1-ctpC*)::Zeo^R^ and Δ(*pacL1-ctpC*):: *dif4* Mtb mutants | |
| *PCR amplification of pacL1 upstream fragment* | |
| 3269Am-Fw | AGCGATACTCGACGATTC |
| 3269-Am-Rv-Zeo | CAGTCGATCCACGTGGAGCATTGCCTGTACCTTTCTTCC |
| *PCR amplification of ctpC downstream fragment* | |
| 3270-Zeo-Av-Fw | CCACTGAGCGTCAGACCCACGTGCTCGCCAACAGTTCCCGGTTGATCC |
| 3270-Av-Rev | AGCCTGGCGGTATTGCTCAC |
|  |  |
| Construct of Δ*pacL1*::*dif4-ctpC*^+^ Mtb mutant | |
| *PCR amplification of pacL1 upstream fragment* | |
| 3269Am-Fw | AGCGATACTCGACGATTC |
| 3269-Am-Rv-Zeo2 | CAGTCGATCCACGTGGAGGCCATTGCCTGTACCTTTCTTCC |
| *PCR amplification of pacL1 downstream fragment* | |
| 3269-Zeo-Av-Fw | CCACTGAGCGTCAGACCCACGTGCTCACCTTGAACTCGCCAGGAC |
| Seq-ctpC-Rev1 | TTCTCGCGCAGGATGAGGCTTG |
|  |  |
| Construct of Δ*pacL2*::*dif6* Mtb mutant | |
| *PCR amplification of pcL2 upstream fragment* | |
| 1993c-Am-Fw | CGTGAGTTGGCGGCTCTG |
| 1993c-Am-Rv-Zeo | CAGTCGATCCACGTGGAGGTAACCACGGTCAGTTCTCC |
| *PCR amplification of pacL2 downstream fragment* | |
| 1993c-Zeo-Av-Fw | CCACTGAGCGTCAGACCCACGTGCTCCGAGTGACGACTGTAGTTGACGCCGAG |
| 1993c-Av-Rv | CAGCGGATAAGCCCATGCGG |
|  |  |
| Construct of Δ(*pacL2*-*ctpG*)::*dif5* Mtb mutant | |
| *PCR amplification of pcL2 upstream fragment* | |
| 1993c-Am-Fw | CGTGAGTTGGCGGCTCTG |
| 1993c-Am-Rv-Zeo | CAGTCGATCCACGTGGAGGTAACCACGGTCAGTTCTCC |
| *PCR amplification of pacL2 downstream fragment* | |
| ctpG-Zeo-Av-Fw | CCACTGAGCGTCAGACCCACGTGCTCGGGGTAGCGACGCGCGGAATCG |
| ctpG-Av-Rv | GCAAGAACACGTTGCCGGG |

**Table EV3. Construction of *M. tuberculosis* mutants.**
