# Supplementary material for: Membrane-associated effluxosomes coordinate multi-metal resistance in Mycobacterium tuberculosis
Source: EMBO J. 2026 Feb 13;45(7):2306–37. doi: 10.1038/s44318-026-00715-1 (PMC13043812; doi:10.1038/s44318-026-00715-1)
Supplement: Supplementary file 5 — Table EV4 [file 44318_2026_715_MOESM5_ESM.docx]

| Plasmids | Description | Vectors linearization cleavage with indicated enzymes or PCR amplification (template/primers) | Primers for inserts amplification (insertion or circularization by In-fusion reaction) | Reference  or source |
| --- | --- | --- | --- | --- |
|  |  |  |  |  |
| Vectors | | | |  |
| pJV53H | Recombineering enzymes expression vector (Hyg^R^, OriMyc) |  |  | PMID: 17179933 |
| pDB60 | Shuttle integrative complementation vector (Strep^R^, attP(L5), Ori *E. coli*) |  |  | Laboratory collection |
| pmsg419 | Empty ATC-on system vector  (Hyg^R^, OriMyc, *tet*- promoter) |  |  | Laboratory collection |
| pGMCS | Shuttle integrative vector (StrepR, attP(L5), Ori *E. coli*) |  |  | PMID: 35961955 |
| pET28 | Proteins expression vector |  |  | Laboratory collection |
|  |  |  |  |  |
| Metal sensitivity |  |  |  |  |
| pDP-MMEP3 | pDB60+cmtR-pacL2-ctpG | EcoR1 (pDB60) | PCR (chromosomal DNA H37Rv/ ODPMMEP7-ODPMMEP8) |  |
| pDP-MMEP4 | pDB60+csoR-pacL3-ctpV | EcoR1 (pDB60) | PCR (chromosomal DNA H37Rv/ ODPMMEP9-ODPMMEP10) |  |
| pDP-MMEP15 | pmsg419+*pacL1-ctpC* | ClaI (pmsg419) | PCR (chromosomal DNA H37Rv/ ODPMMEP40-ODPMMEP41) |  |
| pDP-MMEP16 | pmsg419+*pacL2-ctpG* | ClaI (pmsg419) | PCR (pDP-MMEP3/ ODPMMEP40-ODPMMEP41 |  |
| pDP-MMEP17 | pmsg419+*pacL3-ctpV* | ClaI (pmsg419) | PCR (pDP-MMEP4/ ODPMMEP40-ODPMMEP41 |  |
| pDP-MMEP18 | pDB60+cmtR-ctpG | NcoI+SpHI (pDB60) | ODPMMEP56-ODPMMEP52 +ODPMMEP53-ODPMMEP57 |  |
| pDP-MMEP19 | pDB60+cmtR-pacL2 | NcoI+SpHI (pDB60) | ODPMMEP56-ODPMMEP54 +ODPMMEP55-ODPMMEP57 |  |
| pDP-MMEP20 | pDB60-*pacL1-ctpC* | EcoR1 (pDB60) | ODPMMEP73-ODPMMEP74 |  |
| pDP-MMEP31 | pDB60+*csoR-pacL3-ctpV-Rv0970* | EcoR1 (pDB60) | ODPMMEP9-ODPMMEP59 +ODPMMEP58-ODPMMEP90 |  |
| pDP-MMEP38 | pmsg419+*pacL1-ctpG* | BamHI+AgeI (pmsg419) | ODPMMEP88-ODPMMEP102 +ODPMMEP101-ODPMMEP89 |  |
| pDP-MMEP39 | pmsg419+*pacL3-ctpG* | BamHI+AgeI (pmsg419) | ODPMMEP88-ODPMMEP103 +ODPMMEP101-ODPMMEP89 |  |
| pDP-MMEP53 | pDB60+*cmtR-pacL2*^3EA^-*ctpG* | EcoR1 (pDB60) | ODPMMEP7-ODPMMEP157 +ODPMMEP158-ODPMMEP8 |  |
| pDP-MMEP54 | pDB60+*cmtR-pacL2-ctpG*^APC>AAA^ | EcoR1 (pDB60) | ODPMMEP7-ODPMMEP159 +ODPMMEP160-ODPMMEP8 |  |
| pCG-MMEP1 | pDB60+*cmtR*-*pacL2*^E59A^-*ctpG* | PCR (pDP-MMEP3/ OGCMMEP24-OGCMMEP25) | Circularization |  |
| pCG-MMEP2 | pDB60+*cmtR*-*pacL2*^E71A^-*ctpG* | PCR (pDP-MMEP3/ OGCMMEP20-OGCMMEP21) | Circularization |  |
| pCG-MMEP3 | pDB60+*cmtR*-*pacL2*^ΔE55-A84^-*ctpG* | PCR (pDP-MMEP3/ OGCMMEP16-OGCMMEP18) | Circularization |  |
| pCG-MMEP4 | pDB60+*cmtR*-*pacL2*^ΔL31-A84^-*ctpG* | PCR (pDP-MMEP3/ OGCMMEP16-OGCMMEP19) | Circularization |  |
| pCG-MMEP5 | pDB60+*cmtR*-*pacL2*^G17L+G20L^-*ctpG* | Afe1+Xba1 (pDP-MMEP3) | OGCMMEP11-ODPMMEP149 |  |
| pCG-MMEP6 | pDB60+*cmtR*-*pacL2*^K9A^-*ctpG* | Afe1+Xba1 (pDP-MMEP3) | OGCMMEP13-ODPMMEP149 |  |
|  |  |  |  |  |
| Split GFP | | | | |
| pDP-MMEP 1 | pGMC+*pacL1*-*gfp11*+*ctpA*^MBD^-*gfp1-10* | PCR (pCG-MMEP19/ OGCMMEP29-OGCMMEP30) | ODPMMEP1-ODPMMEP2 |  |
| pCG-MMEP7 | pGMC+*pacL1*-*gfp11*+*gfp1-10* |  |  | PMID: 35961955 |
| pCG-MMEP8 | pGMC+*pacL1*-*gfp11*+*pacL1*-*gfp1-10* | PCR (pCG-MMEP7/ OGCMMEP30-OGCMMEP31) | ODPMMEP32-ODPMMEP33 |  |
| pCG-MMEP9 | pGMC+*pacL2*-*gfp11*+*pacL2*-*gfp1-10* | PCR (pCG-MMEP20/ OGCMMEP30-OGCMMEP31) | OGCMMEP7-OGCMMEP8 |  |
| pCG-MMEP10 | pGMC+*pacL2*-*gfp11*+*pacL1*-*gfp1-10* | PCR (pCG-MMEP8/ OGCMMEP9-OGCMMEP10) | OGCMMEP7-OGCMMEP8 |  |
| pCG-MMEP11 | pGMC+*pacL3*-*gfp11*+*pacL1*-*gfp1-10* | PCR (pCG-MMEP8/ OGCMMEP9-OGCMMEP10) | OGCMMEP41-OGCMMEP42 |  |
| pCG-MMEP12 | pGMC+*pacL2*-*gfp11*+*pacL3*-*gfp1-10* | PCR (pCG-MMEP10/ OGCMMEP30-OGCMMEP31) |  |  |
| pCG-MMEP13 | pGMC+*Rv1488*-*gfp11*+*pacL1*-*gfp1-10* | PCR (pCG-MMEP8/ OGCMMEP9-OGCMMEP10) | PCR (chromosomal DNA H37Rv/ OGCMMEP45-OGCMMEP46) |  |
| pCG-MMEP14 | pGMC+*pacL2*^E59A^-*gfp11*+*pacL2*^E59A^-*gfp1-10* | PCR (pCG-MMEP25/ OGCMMEP28-OGCMMEP29) | PCR (pCG-MMEP25/ OGCMMEP26-OGCMMEP27) |  |
| pCG-MMEP15 | pGMC+*pacL2*^E71A^-*gfp11*+*pacL2*^E71A^-*gfp1-10* | PCR (pCG-MMEP26/ OGCMMEP28-OGCMMEP29) | PCR (pCG-MMEP26/ OGCMMEP26-OGCMMEP27) |  |
| pCG-MMEP16 | pGMC+*pacL1*-*gfp11*+*ctpG^MBD^*-*gfp1-10* | PCR (pCG-MMEP19/ OGCMMEP29-OGCMMEP30) | ODPMMEP37-ODPMMEP38 |  |
| pCG-MMEP17 | pGMC+*pacL2*-*gfp11*+*ctpG^MBD^*-*gfp1-10* | PCR (pCG-MMEP20/ OGCMMEP30-OGCMMEP31) | ODPMMEP37-ODPMMEP38 |  |
| pCG-MMEP18 | pGMC+*pacL3*-*gfp11*+*ctpG^MBD^*-*gfp1-10* | PCR (pCG-MMEP21/ OGCMMEP30-OGCMMEP31) | ODPMMEP37-ODPMMEP38 |  |
| pCG-MMEP19 | pGMC+*pacL1*-*gfp11*+*ctpC^MBD^*-*gfp1-10* |  |  | ^1^ |
| pCG-MMEP20 | pGMC+*pacL2*-*gfp11*+*ctpC^MBD^*-*gfp1-10* | PCR (pCG-MMEP19/ OGCMMEP9-OGCMMEP10) | OGCMMEP7-OGCMMEP8 |  |
| pCG-MMEP21 | pGMC+*pacL3*-*gfp11*+*ctpC^MBD^*-*gfp1-10* | PCR (pCG-MMEP19/ OGCMMEP9-OGCMMEP10) | OGCMMEP41-OGCMMEP42 |  |
| pCG-MMEP22 | pGMC+*pacL1*-*gfp11*+*ctpV^MBD^*-*gfp1-10* | PCR (pCG-MMEP19/ OGCMMEP29-OGCMMEP30) | ODPMMEP37-ODPMMEP38 |  |
| pCG-MMEP23 | pGMC+*pacL2*-*gfp11*+*ctpV^MBD^*-*gfp1-10* | PCR (pCG-MMEP20/ OGCMMEP29-OGCMMEP30) | ODPMMEP37-ODPMMEP38 |  |
| pCG-MMEP24 | pGMC+*pacL3*-*gfp11*+*ctpV^MBD^*-*gfp1-10* | PCR (pCG-MMEP21/ OGCMMEP29-OGCMMEP30) | ODPMMEP37-ODPMMEP38 |  |
| pCG-MMEP25 | pGMC+*pacL2*^E59A^-*gfp11*+*ctpG*^MBD^-*gfp1-10* | PCR (pCG-MMEP17/ OGCMMEP24-OGCMMEP25) | Circularization |  |
| pCG-MMEP26 | pGMC+*pacL2*^E71A^-*gfp11*+*ctpG*^MBD^-*gfp1-10* | PCR (pCG-MMEP17/ OGCMMEP20-OGCMMEP21) | Circularization |  |
| pCG-MMEP27 | pGMC+*pacL2^ΔE55-E84^*-*gfp11*+*ctpG^MBD^*-*gfp1-10* | PCR (pCG-MMEP17/ OGCMMEP16-OGCMMEP18) | Circularization |  |
| pCG-MMEP28 | pGMC+*pacL2^ΔL31-E84^*-*gfp11*+*ctpG^MBD^*-*gfp1-10* | PCR (pCG-MMEP17/ OGCMMEP16-OGCMMEP19) | Circularization |  |
|  |  |  |  |  |
| Microscopy | | | | |
| pDP-MMEP44 | pDB60+*cmtR-pacL2-mEos3.2-ctpG* | EcoR1 (pDB60) | ODPMMEP7-ODPMMEP13 +ODPMMEP129-ODPMMEP130 +ODPMMEP132-ODPMMEP8 |  |
| pDP-MMEP45 | pDB60+*cmtR-pacL2-ctpG-mEos*3.2 | EcoR1 (pDB60) | ODPMMEP7-ODPMMEP133 +ODPMMEP129-ODPMMEP134 |  |
| pDP-MMEP46 | pDB60+*cmtR-pacL2-mTurquoise-ctpG* | EcoR1 (pDB60) | ODPMMEP7-ODPMMEP137 +ODPMMEP139-ODPMMEP140 +ODPMMEP138-ODPMMEP8 |  |
| pDP-MMEP56 | pDB60+*cmtR-ctpG-mVenus* | EcoR1 (pDB60) | ODPMMEP7-ODPMMEP142 +ODPMMEP139-ODPMMEP142 |  |
| pDP-MMEP57 | pDB60+*cmtR-pacL2-ctpG-mVenus* | EcoR1 (pDB60) | ODPMMEP7-ODPMMEP142 +ODPMMEP139-ODPMMEP142 |  |
| pDP-MMEP60 | pDB60+*cmtR-pacL2-mTurquoise-ctpG-mVenus* | EcoR1 (pDB60) | ODPMMEP7-ODPMMEP137 +ODPMMEP139-ODPMMEP140 +ODPMMEP138-ODPMMEP142 +ODPMMEP139-ODPMMEP141 |  |
| pDP-MMEP61 | pDB60+*cmtR-pacL2-mTurquoise+pacL1-mVenus* | EcoR1+XbaI (pDB60) | ODPMMEP165-ODPMMEP166 +ODPMMEP167-ODPMMEP168 |  |
| pDP-MMEP65 | pDB60+*cmtR-pacL2-ctpG-mVenus+pacL1-ctpC-mTurquoise* | XbaI+NotI (pDP-MMEP57) | ODPMMEP184-ODPMMEP185 |  |
| pCG-MMEP29 | pDB60+*cmtR*-*pacL2*^E59A^*-mTurquoise-ctpG-mVenus* | PCR (pCG-MMEP60/ OGCMMEP24-OGCMMEP25) | Circularization |  |
| pCG-MMEP30 | pDB60+*cmtR*-*pacL2*^E71A^*-mTurquoise-ctpG-mVenus* | PCR (pCG-MMEP60/ OGCMMEP20-OGCMMEP21) | Circularization |  |
| pCG-MMEP31 | pDB60+*cmtR*-*pacL2*^ΔE55-A84^-*mTurquoise-ctpG-mVenus* | PCR (pCG-MMEP60/ OGCMMEP16-OGCMMEP18) | Circularization |  |
| pCG-MMEP32 | pDB60+*cmtR*-*pacL2*^ΔL31-A84^*-mTurquoise-ctpG-mVenus* | PCR (pCG-MMEP60/ OGCMMEP16-OGCMMEP19) | Circularization |  |
| pCG-MMEP33 | pGMCS+*pacL1-mTurquoise*-*ctpC-mVenus* | |  | PMID: 35961955 |
| pCG-MMEP34 | pGMCS+*pacL1*^Δ54-86^-*mTurquoise-ctpC-mVenus* | PCR (pDP-MMEP33/ OGCMMEP34-OGCMMEP35) | Circularization |  |
| pCG-MMEP35 | pGMCS+*pacL1*^Δ37-86^*-mTurquoise-ctpC-mVenus* | PCR (pDP-MMEP33/ OGCMMEP34-OGCMMEP36) | Circularization |  |
| pCG-MMEP36 | pDB60+*cmtR*-*pacL2*^G17L+G20L^*-mTurquoise-ctpG-mVenus* | PCR (pDP-MMEP60/ OGCMMEP16-OGCMMEP18) | Circularization |  |
| pCG-MMEP37 | pDB60+*cmtR*-*pacL2*^K9A^-*mTurquoise*-*ctpG-mVenus* | PCR (pDP-MMEP60/ OGCMMEP16-OGCMMEP19) | Circularization |  |
| pCG-MMEP38 | pDB60+*cmtR*-*pacL2*^G17L+G20L^*-mEos3.2*-*ctpG-mVenus* | Afe1+Xba1 (pDP-MMEP60) | OGCMMEP11-ODPMMEP149 |  |
| pCG-MMEP39 | pDB60+*cmtR*-*pacL2*^K9A^*-mEos3.2*-*ctpG-mVenus* | Afe1+Xba1 (pDP-MMEP60) | OGCMMEP13-ODPMMEP149 |  |
|  |  |  |  |  |
| Proximity labelling | | | | |
| pDP-MMEP23 | pDB60+pacL1IntALFA-ctpC | EcoR1 (pDB60) | ODPMMEP73-ODPMMEP66 +ODPMMEP67-ODPMMEP74 |  |
| pDP-MMEP24 | pDB60+pacL1-CterALFA-ctpC | EcoR1 (pDB60) | ODPMMEP73-ODPMMEP68 +ODPMMEP69-ODPMMEP74 |  |
|  |  |  |  |  |
| Protein purification | | | | |
| pCG-MMEP40 | pET28+His-Tev-PacL1 |  |  | PMID: 35961955 |
| pCG-MMEP41 | pET28+His-Tev-PacL1ΔC5 | |  | PMID: 35961955 |
| pCG-MMEP42 | pET28+His-Tev-PacL2 | PCR (pET28a/ OGCMMEP49-OGCMMEP50) | OGCMMEP47-OGCMMEP48 |  |

**Table EV4. Plasmids used in this work and cloning methods.**
