# Supplementary material for: Membrane-associated effluxosomes coordinate multi-metal resistance in Mycobacterium tuberculosis
Source: EMBO J. 2026 Feb 13;45(7):2306–37. doi: 10.1038/s44318-026-00715-1 (PMC13043812; doi:10.1038/s44318-026-00715-1)
Supplement: Supplementary file 6 — Table EV5 [file 44318_2026_715_MOESM6_ESM.docx]

| Plasmids | Sequences |
| --- | --- |
| ODPMMEP1 | GAGCGCCTCGCCATGACGACGGCCGTGACCG |
| ODPMMEP2 | AGACAGCCCTGGACCATCACTCGCGCTCCGACCG |
| ODPMMEP7 | TCCAGCTGCAGAATTACTACACGCCGAAAGCGC |
| ODPMMEP8 | GATAAGCTTCGAATTCTACCCCGGGATAGTCCGATC |
| ODPMMEP9 | TCCAGCTGCAGAATTTTCATAATCGGTCAGCTGCAGG |
| ODPMMEP10 | GATAAGCTTCGAATTTCATGCGGTTCGGCCGTC |
| ODPMMEP13 | CTAGTATGCATCATAAAGCACCGTGTGTTCGCC |
| ODPMMEP40 | CAGAAAGGAGGCCATATGGCGATACAAGTGTTCTTGGC |
| ODPMMEP41 | AGGTCGACGGTATCGCTAGCGGTCCAGGCGGTAG |
| ODPMMEP52 | CACGGTCAGTTCTCCTTTGC |
| ODPMMEP53 | GGAGAACTGACCGTGATCGGTGAGGAGGCGCCC |
| ODPMMEP54 | AACGGTCAGCTGGACCTCGG |
| ODPMMEP55 | GTCCAGCTGACCGTTTAGAATTCGAAGCTTATCGATGTCGACGTAG |
| ODPMMEP56 | ATCTAGATATCCATGGATCCAGCTGC |
| ODPMMEP57 | GGAGGACGTTGTAGGCATGCC |
| ODPMMEP58 | TTTGCCGGTGGACCTCTGTTC |
| ODPMMEP59 | AGGTCCACCGGCAAACAACTG |
| ODPMMEP66 | ACGCCGCAGTTCCTCCTCCAACCGGCTTGGCGCTTTGGCCGCGGCCTTT |
| ODPMMEP67 | GAGGAACTGCGGCGTCGGCTCACGGAGCCACCGCTTCGTCAGACCGCGG |
| ODPMMEP68 | CGCCGCAGTTCCTCCTCCAACCGGCTTGGGTGGTCGTGGTCGTGCAGG |
| ODPMMEP69 | GGAGGAACTGCGGCGTCGGCTCACGGAGTGAGCGCCTCGCCATGACC |
| ODPMMEP73 | TCCAGCTGCAGAATTAGGTAGCCGTGCTGTCGCTG |
| ODPMMEP74 | GATAAGCTTCGAATTCTAGCGGTCCAGGCGGTAGC |
| ODPMMEP88 | TGATGGTACCGGATCCGTGGC |
| ODPMMEP89 | ACGATTAATCACCGGTGTATGGTCCG |
| ODPMMEP90 | GATAAGCTTCGAATTTCAAACCGGAAACAGCATGG |
| ODPMMEP101 | GTGACGACTGTAGTTGACGCCG |
| ODPMMEP102 | AACTACAGTCGTCACGGCGAGGCGCTCAGTGGTC |
| ODPMMEP103 | AACTACAGTCGTCACGGCGAGGCGCTCAGTGGTCATGACCGTCGTCC |
| ODPMMEP129 | GGTCCGGGTCTGTCGGGC |
| ODPMMEP130 | TCACCGACGGGCGTTATCCG |
| ODPMMEP132 | AACGCCCGTCGGTGAGCGCCTCGCCGTGACGACTGTAGTTGACGCC |
| ODPMMEP134 | GATAAGCTTCGAATTTCACCGACGGGCGTTATCCG |
| ODPMMEP136 | CCTCGAGGTCGACGGTATCG |
| ODPMMEP137 | GCCCGAACCCTCCAGCTCGTCGACCCTGGCGCC |
| ODPMMEP138 | GAACTCTACAAGTAAGTGACGACTGTAGTTGACGCC |
| ODPMMEP139 | CTGGAGGGTTCGGGCGTCAGC |
| ODPMMEP140 | TTACTTGTAGAGTTCGTCCATGCCG |
| ODPMMEP141 | GATAAGCTTCGAATTTTACTTGTAGAGTTCGTCCATGCCG |
| ODPMMEP142 | GCCCGAACCCTCCAGCCCCGGGATAGTCCGATCAGG |
| ODPMMEP157 | GACTGTCAGCCGGGCCGACGCGGCCGCCGCGGCCGCGCGCCGGGTTCCG |
| ODPMMEP158 | GCCCGGCTGACAGTCGCCGACGTCGTTGCCGCCGCTCGCGGGCGCATCG |
| ODPMMEP159 | CGCGGCCGCGGCGGCGGCGACTAATACCACGAG |
| ODPMMEP160 | GCCGCCGCGGCCGCGCTGGCCATCGCCGTTC |
| ODPMMEP165 | GATCTTTAAATCTAGTTACTTGTAGAGTTCGTCCATGCC |
| ODPMMEP166 | ACTACACGCCGAAAGCGCGATCACACCG |
| ODPMMEP167 | CTTTCGGCGTGTAGTATACTCGACGATTCTCCCGC |
| ODPMMEP168 | GATAAGCTTCGAATTTTACTTGTACAGCTCGTCCATGC |
| ODPMMEP184 | TCCATGGATATCTAGGAGTTGGTGGACGGCCTG |
| ODPMMEP185 | GCAACGCGTGCGGCCTTACTTGTAGAGTTCGTCCATGC |
| OGC-MMEP7 | AAGGTACAGGCAATGGTTACGCATGAGCTATTGG |
| OGC-MMEP8 | ACCGGATCCTTCCAGCTCGTCGACCCTGGCG |
| OGC-MMEP9 | CATTGCCTGTACCTTTCTTCC |
| OGC-MMEP10 | CTGGAAGGATCCGGTGGCG |
| OGC-MMEP11 | GGCGCCGTACCCAGCGCTTTGCGCAGCGTTTCGTAGGCGCTCACCagGACCAGcagGG TGAGCACCGCCCCGGCC |
| OGC-MMEP12 | GCTGGGTACGGCGCCC |
| OGC-MMEP13 | GCGGCCGGGGCGGTG |
| OGC-MMEP14 | CACCGCCCCGGCCGCCgcAACCAATAGCTCATGCGTAACCATTGC |
| OGC-MMEP15 | GGCGCCGTACCCAGCGCTTTGCGCAGCGTTTCGTAGGCGCTCACCCCGACCAGTCCG GTGAGCACCGCCCCGGCCGCCgcAACCAATAGCTCATGCGTAACCATTGC |
| OGC-MMEP16 | GGCGCCAGGGTCGACGAG |
| OGC-MMEP17 | GTCGACCCTGGCGCCGCGCCCGCGAGCCTC |
| OGC-MMEP18 | GTCGACCCTGGCGCCCGCGCGCCGGGTTCC |
| OGC-MMEP19 | GTCGACCCTGGCGCCCGCTTTGCGCAGCGTTTCG |
| OGC-MMEP20 | GCTCGCGGGCGCATCGGTGAG |
| OGC-MMEP21 | GATGCGCCCGCGAGCagCGGCAACGACGTCGGCG |
| OGC-MMEP22 | GATGCGCCCGCGAGCCTCGGCAACGACGgCGGCGACTGTCAGCCG |
| OGC-MMEP23 | GATGCGCCCGCGAGCagCGGCAACGACGgCGGCGACTGTCAGCCG |
| OGC-MMEP24 | TCGGCCCGGCTGACAGTC |
| OGC-MMEP25 | TGTCAGCCGGGCCGACgCGGCCGCCGCCTCCGCGC |
| OGC-MMEP26 | GAGCGCCTCGCCATGGTTACGCATGAGCTATTGG |
| OGC-MMEP27 | AGACAGCCCTGGACCCTCGTCGACCCTGGCG |
| OGC-MMEP28 | CATGGCGAGGCGCTCAAGATG |
| OGC-MMEP29 | GGTCCAGGGCTGTCTGGCCT |
| OGC-MMEP30 | CATGGCGAGGCGCTCAAG |
| OGC-MMEP34 | GACCTGCACGACCACGACCAC |
| OGC-MMEP35 | GTGGTCGTGCAGGTCCTTGCGGGTTCCGCGCAGACC |
| OGC-MMEP36 | GTGGTCGTGCAGGTCAAGCGGCGCTTTGGCCGCGG |
| OGC-MMEP37 | GAGCGCCTCGCCATGacgactgtagttgacgccg |
| OGC-MMEP38 | AGACAGCCCTGGACCagcatacgcgggcaccg |
| OGC-MMEP39 | GAGCGCCTCGCCATGACCACTGACGTTCTTTCTG |
| OGC-MMEP40 | AGACAGCCCTGGACCggcacgggcgggcac |
| OGC-MMEP41 | AAGGTACAGGCAATGGTGTGGCATGGATTCCTAG |
| OGC-MMEP42 | ACCGGATCCTTCCAGGTGGTCATGACCGTCGTCCG |
| OGC-MMEP45 | GGAAGAAAGGTACAGGCAATGcaaggagccgttgctgg |
| OGC-MMEP46 | CGCCACCGGATCCTTCCAGttgagtcaacctggggggc |
| OGC-MMEP47 | ACCATGGGCAGCAGCCACC |
| OGC-MMEP48 | GTTAGCAGCCGGATCttaTTCGTCAAC |
| 0GC-MMEP49 | GATCCGGCTGCTAACAAAG |
| 0GC-MMEP50 | GCTGCTGCCCATGGTATATCT |

**Table EV5. Primers used in this work.**
