# Supplementary material for: Membrane-associated effluxosomes coordinate multi-metal resistance in Mycobacterium tuberculosis
Source: EMBO J. 2026 Feb 13;45(7):2306–37. doi: 10.1038/s44318-026-00715-1 (PMC13043812; doi:10.1038/s44318-026-00715-1)
Supplement: Supplementary file 7 — Table EV6 [file 44318_2026_715_MOESM7_ESM.docx]

| Figures | Strains/conditions | n | p-values relative to the reference strain | |  | Figures | Strains/conditions | n | p-values relative to the reference strain |
| --- | --- | --- | --- | --- | --- | --- | --- | --- | --- |
|  |  |  | * | ** |  |  |  |  | * |
| 1B | *tet-empty** | 6 | N/A |  |  |  |  |  |  |
|  | *tet-pacL1-ctpC* | 6 | <0.0001 |  |  | EV1A | empty vector* | 12 | N/A |
|  | *tet-pacL2-ctpG* | 6 | 0.9990 |  |  |  | *ctpC* operon | 6 | <0.0001 |
|  | *tet-pacL3-ctpV* | 6 | 0.9778 |  |  |  | *ctpG* operon | 3 | 0.7751 |
|  |  |  |  |  |  |  | *ctpV* operon | 3 | 0.9973 |
| 1C | *tet-empty** | 6 | N/A |  |  |  |  |  |  |
|  | *tet-pacL1-ctpC* | 6 | 0.0072 |  |  | EV1B | empty vector* | 12 | N/A |
|  | *tet-pacL2-ctpG* | 6 | <0.0001 |  |  |  | *ctpC* operon | 3 | >0.9999 |
|  | *tet-pacL3-ctpV* | 6 | 0.3854 |  |  |  | *ctpG* operon | 6 | <0.0001 |
|  |  |  |  |  |  |  | *ctpG* operon^(APC>AAA)^ | 3 | 0.4513 |
| 1D | *tet-empty** | 6 | N/A |  |  |  | *ctpV* operon | 3 | >0.9999 |
|  | *tet-pacL1-ctpC* | 6 | 0.9232 |  |  |  |  |  |  |
|  | *tet-pacL2-ctpG* | 6 | 0.1903 |  |  | EV1C | empty vector* | 9 | N/A |
|  | *tet-pacL3-ctpV* | 6 | 0.3008 |  |  |  | *ctpC* operon | 3 | 0.2521 |
|  |  |  |  |  |  |  | *ctpG* operon | 3 | 0.9371 |
| 2A | empty vector* | 9 | N/A |  |  |  | *ctpV* operon | 3 | >0.9999 |
|  | *cmtR-pacL2-ctpG* | 9 | <0.0001 |  |  |  |  |  |  |
|  | *cmtR-ctpG* | 9 | <0.0001 |  |  | EV1D | empty vector* | 9 | N/A |
|  | *cmtR-pacL2* | 6 | 0.8704 |  |  |  | *ctpC* operon | 3 | 0.7489 |
|  |  |  |  |  |  |  | *ctpG* operon | 3 | 0.9889 |
| 2E | background | 10 | N/A |  |  |  | *ctpV* operon | 3 | 0.9952 |
|  | PacL2^mT^ (Cd-)* | 20 | N/A |  |  |  |  |  |  |
|  | PacL2^mT^ (Cd+) | 20 | <0.0001 |  |  | EV1E | empty vector* | 9 | N/A |
|  |  |  |  |  |  |  | *ctpC* operon | 3 | 0.0945 |
| 2H | background | 19 | N/A |  |  |  | *ctpG* operon | 3 | >0.9999 |
|  | CtpG^mV^ (+PacL2)* | 20 | N/A |  |  |  | *ctpV* operon | 3 | 0.8284 |
|  | CtpG^mV^ (-PacL2) | 20 | <0.0001 |  |  |  |  |  |  |
|  |  |  |  |  |  | EV1F | empty vector* | 9 | N/A |
| 2J | empty vector | 6 | <0.0001 |  |  |  | *ctpC* operon | 3 | 0.2650 |
|  | *cmtR-pacL2-ctpG** | 6 | N/A |  |  |  | *ctpG* operon | 3 | 0.7608 |
|  | *cmtR-pacL2*^E59A^*-ctpG* | 6 | <0.0001 |  |  |  | *ctpV* operon | 3 | 0.2105 |
|  | *cmtR-pacL2*^E71A^*-ctpG* | 6 | <0.0001 |  |  |  |  |  |  |
|  | *cmtR-pacL2*^3EA^*-ctpG* | 6 | <0.0001 |  |  | EV1G | empty vector* | 3 | N/A |
|  |  |  |  |  |  |  | *ctpV* full operon | 3 | 0.8822 |
| 2L | background | 12 | <0.0001 |  |  |  |  |  |  |
|  | PacL2^mT-WT^* | 20 | N/A |  |  | EV1H | empty vector* | 3 | N/A |
|  | PacL2^mT-E59A^ | 20 | 0.3028 |  |  |  | *ctpV* full operon | 3 | 0.8657 |
|  | PacL2^mT-E71A^ | 20 | 0.8831 |  |  |  |  |  |  |
|  |  |  |  |  |  | EV1I | *tet-empty* (Zn)*** | 3 | N/A |
| 2M | background | 12 | <0.0001 |  |  |  | *tet-pacL1-ctpC* (Zn) | 3 | >0.9999 |
|  | CtpG^mV^ (PacL2^WT^)* | 20 | N/A |  |  |  | *tet-pacL2-ctpG* (Zn) | 3 | >0.9999 |
|  | CtpG^mV^ (PacL2^E59A^) | 20 | <0.0001 |  |  |  | *tet-pacL3-ctpV* (Zn) | 3 | >0.9999 |
|  | CtpG^mV^ (PacL2^E71A^) | 20 | <0.0001 |  |  |  | *tet-empty* (Cd)*** | 3 | N/A |
|  |  |  |  |  |  |  | *tet-pacL1-ctpC* (Cd) | 3 | >0.9999 |
| 2O | Control | 6 | <0.0001 |  |  |  | *tet-pacL2-ctpG* (Cd) | 3 | >0.9999 |
|  | PacL2/CtpG^MBD^* | 6 | N/A |  |  |  | *tet-pacL3-ctpV* (Cd) | 3 | >0.9999 |
|  | PacL2^E59A^/CtpG^MBD^ | 6 | <0.0001 |  |  |  | *tet-empty* (Cu)*** | 3 | N/A |
|  | PacL2^E71A^/CtpG^MBD^ | 6 | <0.0001 |  |  |  | *tet-pacL1-ctpC* (Cu) | 3 | >0.9999 |
|  | PacL2^E59A+E71A^/CtpG^MBD^ | 6 | <0.0001 |  |  |  | *tet-pacL2-ctpG* (Cu) | 3 | >0.9999 |
|  |  |  |  |  |  |  | *tet-pacL3-ctpV* (Cu) | 3 | >0.9999 |
| 3C | PacL2* | 22 | N/A |  |  |  | *tet-empty* (Ni)*** | 3 | N/A |
|  | CpG | 37 | 0.0417 |  |  |  | *tet-pacL1-ctpC* (Ni) | 3 | 0.9769 |
|  |  |  |  |  |  |  | *tet-pacL2-ctpG* (Ni) | 3 | >0.9999 |
| 3D | PacL2* | 280 | N/A |  |  |  | *tet-pacL3-ctpV* (Ni) | 3 | 0.7313 |
|  | CpG | 393 | <0.0001 |  |  |  | *tet-empty* (Mn)*** | 3 | N/A |
|  |  |  |  |  |  |  | *tet-pacL1-ctpC* (Mn) | 3 | >0.9999 |
| 3E | PacL2* | 22 | N/A |  |  |  | *tet-pacL2-ctpG* (Mn) | 3 | >0.9999 |
|  | CtpG | 37 | <0.0001 |  |  |  | *tet-pacL3-ctpV* (Mn) | 3 | >0.9999 |
|  |  |  |  |  |  |  | *tet-empty* (Fe)*** | 3 | N/A |
| 4A | Control | 20 | <0.0001 |  |  |  | *tet-pacL1-ctpC* (Fe) | 3 | >0.9999 |
|  | PacL1/PacL1 | 13 | <0.0001 |  |  |  | *tet-pacL2-ctpG* (Fe) | 3 | >0.9999 |
|  | PacL2/PacL2 | 6 | 0.0004 |  |  |  | *tet-pacL3-ctpV* (Fe) | 3 | 0.9996 |
|  | PacL2/PacL1 | 5 | 0.0005 |  |  |  |  |  |  |
|  | PacL3/PacL1 | 5 | 0.0005 |  |  | EV1J | *tet-empty* (Zn)*** | 3 | N/A |
|  | PacL2/PacL3 | 7 | 0.0001 |  |  |  | *tet-pacL1-ctpC* (Zn) | 3 | <0.0001 |
|  | Rv1488/PacL1* | 7 | N/A |  |  |  | *tet-pacL2-ctpG* (Zn) | 3 | >0.9999 |
|  |  |  |  |  |  |  | *tet-pacL3-ctpV* (Zn) | 3 | 0.4314 |
| 4C | Background* | 20 | N/A | <0.0001 |  |  | *tet-empty* (Cd)*** | 3 | N/A |
|  | PacL1^mV^** | 20 | <0.0001 | N/A |  |  | *tet-pacL1-ctpC* (Cd) | 3 | <0.0001 |
|  | PacL1^mV^+Zn | 20 | <0.0001 | <0.0001 |  |  | *tet-pacL2-ctpG* (Cd) | 3 | <0.0001 |
|  | PacL1^mV^+Cd | 20 | <0.0001 | 0.0768 |  |  | *tet-pacL3-ctpV* (Cd) | 3 | 0.0204 |
|  | PacL1^mV^+Zn+Cd | 20 | <0.0001 | <0.0001 |  |  | *tet-empty* (Cu)*** | 3 | N/A |
|  |  |  |  |  |  |  | *tet-pacL1-ctpC* (Cu) | 3 | 0.9722 |
| 4D | Background | 20 | 0.0075 |  |  |  | *tet-pacL2-ctpG* (Cu) | 3 | >0.9999 |
|  | PacL2^mV^* | 20 | N/A |  |  |  | *tet-pacL3-ctpV* (Cu) | 3 | >0.9999 |
|  | PacL2^mV^+Zn | 20 | 0.2180 |  |  |  | *tet-empty* (Ni)*** | 3 | N/A |
|  | PacL2^mV^+Cd | 20 | <0.0001 |  |  |  | *tet-pacL1-ctpC* (Ni) | 3 | 0.9400 |
|  | PacL2^mV^+Zn+Cd | 20 | <0.0001 |  |  |  | *tet-pacL2-ctpG* (Ni) | 3 | >0.9999 |
|  |  |  |  |  |  |  | *tet-pacL3-ctpV* (Ni) | 3 | 0.9999 |
| 4F | Control* | 16 | N/A | N/A |  |  | *tet-empty* (Mn)*** | 3 | N/A |
|  | PacL1/CtpA^MBD^** | 3 | 0.8305 | N/A |  |  | *tet-pacL1-ctpC* (Mn) | 3 | >0.9999 |
|  | PacL1/CtpG^MBD^ | 4 | <0.0001 | <0.0001 |  |  | *tet-pacL2-ctpG* (Mn) | 3 | >0.9999 |
|  | PacL2/CtpG^MBD^ | 12 | <0.0001 | <0.0001 |  |  | *tet-pacL3-ctpV* (Mn) | 3 | >0.9999 |
|  | PacL3/CtpG^MBD^ | 7 | <0.0001 | <0.0001 |  |  | *tet-empty* (Fe)*** | 3 | N/A |
|  |  |  |  |  |  |  | *tet-pacL1-ctpC* (Fe) | 3 | >0.9999 |
| 4G | Control* | 21 | N/A |  |  |  | *tet-pacL2-ctpG* (Fe) | 3 | >0.9999 |
|  | PacL1/CtpC^MBD^ | 15 | <0.0001 |  |  |  | *tet-pacL3-ctpV* (Fe) | 3 | >0.9999 |
|  | PacL2/CtpC^MBD^ | 7 | <0.0001 |  |  |  |  |  |  |
|  | PacL3/CtpC^MBD^ | 7 | <0.0001 |  |  | EV1K | *cmtR-ctpG** | 6 | N/A |
|  |  |  |  |  |  |  | *cmtR-pacL2-ctpG* | 6 | <0.0001 |
| 4H | Control* | 10 | N/A |  |  |  | *cmtR-pacL2*^mT^*-ctpG* | 6 | 0.0235 |
|  | PacL1/CtpV^MBD^ | 9 | <0.0001 |  |  |  |  |  |  |
|  | PacL2/CtpV^MBD^ | 7 | <0.0001 |  |  | EV1L | empty vector* | 6 | N/A |
|  | PacL3/CtpV^MBD^ | 7 | <0.0001 |  |  |  | *cmtR-pacL2-ctpG* | 6 | <0.0001 |
|  |  |  |  |  |  |  | *cmtR-pacL2-ctpG*^mV^ | 6 | <0.0001 |
| 4I | *tet-empty** | 3 | N/A |  |  |  |  |  |  |
|  | *tet-pacL1-ctpG* | 3 | <0.0001 |  |  | EV5C | TurboID+empty* | 3 | N/A |
|  | *tet-pacL2-ctpG* | 3 | <0.0001 |  |  |  | TurboID+*pacL1-ctpC* | 3 | <0.0001 |
|  | *tet-pacL3-ctpG* | 3 | <0.0001 |  |  |  | TurboID+*pacL1*^(Int-ALFA)^-*ctpC* | 3 | <0.0001 |
|  |  |  |  |  |  |  | TurboID+*pacL1*^(Cter-ALFA)^-*ctpC* | 3 | <0.0001 |
| 4K | background | 10 | N/A |  |  |  |  |  |  |
|  | PacL2^mT^ +ATC* | 20 | N/A |  |  | EV5D | TurboID+empty* | 3 | N/A |
|  | PacL2^mT^ -ATC | 20 | <0.0001 |  |  |  | TurboID+*pacL1-ctpC* | 3 | <0.0001 |
|  |  |  |  |  |  |  | TurboID+*pacL1*^(Int-ALFA)^-*ctpC* | 3 | <0.0001 |
| 4L | background | 10 | N/A |  |  |  | TurboID+*pacL1*^(Cter-ALFA)^-*ctpC* | 3 | <0.0001 |
|  | CtpG^mV^ +ATC* | 20 | N/A |  |  |  |  |  |  |
|  | CtpG^mV^ -ATC | 20 | <0.0001 |  |  |  |  |  |  |
|  |  |  |  |  |  |  |  |  |  |
| 6C | background | 21 | <0.0001 |  |  |  |  |  |  |
|  | PacL2^mT-WT^* | 20 | N/A |  |  |  |  |  |  |
|  | PacL2^mT-∆55-84^ | 20 | 0.0002 |  |  |  |  |  |  |
|  |  |  |  |  |  |  |  |  |  |
| 6D | background | 21 | <0.0001 |  |  |  |  |  |  |
|  | CtpG^mV^ (PacL2^WT^)* | 20 | N/A |  |  |  |  |  |  |
|  | CtpG^mV^ (PacL2^∆55-84^) | 20 | <0.0001 |  |  |  |  |  |  |
|  |  |  |  |  |  |  |  |  |  |
| 6E | Control | 8 | <0.0001 |  |  |  |  |  |  |
|  | PacL2/CtpG^MBD^* | 8 | N/A |  |  |  |  |  |  |
|  | PacL2^Δ55-A84^/CtpG^MBD^ | 8 | <0.0001 |  |  |  |  |  |  |
|  |  |  |  |  |  |  |  |  |  |
| 6F | empty vector | 6 | <0.0001 |  |  |  |  |  |  |
|  | *cmtR-pacL2-ctpG** | 6 | N/A |  |  |  |  |  |  |
|  | *cmtR-pacL2*^Δ55-A84^*-ctpG* | 6 | <0.0001 |  |  |  |  |  |  |
|  |  |  |  |  |  |  |  |  |  |
| 6H | background | 12 | <0.0001 |  |  |  |  |  |  |
|  | PacL1^mT-WT^* | 16 | N/A |  |  |  |  |  |  |
|  | PacL1^mT-∆54-86^ | 16 | 0.8032 |  |  |  |  |  |  |
|  | PacL1^mT-∆37-86^ | 16 | <0.0001 |  |  |  |  |  |  |
|  |  |  |  |  |  |  |  |  |  |
| 6I | background | 12 | <0.0001 |  |  |  |  |  |  |
|  | CtpC^mV^ (PacL1^WT^)* | 16 | N/A |  |  |  |  |  |  |
|  | CtpC^mV^ (PacL1^∆54-86^) | 16 | <0.0001 |  |  |  |  |  |  |
|  | CtpC^mV^ (PacL1^∆37-86^) | 16 | <0.0001 |  |  |  |  |  |  |
|  |  |  |  |  |  |  |  |  |  |
| 7B | empty vector | 11 | <0.0001 |  |  |  |  |  |  |
|  | PacL2^WT^* | 11 | N/A |  |  |  |  |  |  |
|  | PacL2^G17L+G20L^ | 6 | <0.0001 |  |  |  |  |  |  |
|  | PacL2^K9A^ | 9 | <0.0001 |  |  |  |  |  |  |
|  |  |  |  |  |  |  |  |  |  |
| 7D | background | 21 | <0.0001 |  |  |  |  |  |  |
|  | CtpG^mV^+Cd (PacL2^WT^)* | 20 | N/A |  |  |  |  |  |  |
|  | CtpG^mV^+Cd (PacL2^G17L+G20L^) | 14 | 0.9654 |  |  |  |  |  |  |
|  | CtpG^mV^+Cd (PacL2^K9A^) | 20 | <0.0001 |  |  |  |  |  |  |
|  |  |  |  |  |  |  |  |  |  |
| 7E | background -Cd | 24 | <0.0001 | N/A |  |  |  |  |  |
|  | PacL2^mT-WT^ -Cd* | 20 | N/A | N/A |  |  |  |  |  |
|  | PacL2^mT-G17L+G20L^ -Cd | 20 | <0.0001 | N/A |  |  |  |  |  |
|  | PacL2^mT-K9A^ -Cd | 20 | 0.0008 | N/A |  |  |  |  |  |
|  | background +Cd | 21 | N/A | <0.0001 |  |  |  |  |  |
|  | PacL2^mT-WT^ +Cd** | 20 | N/A | N/A |  |  |  |  |  |
|  | PacL2^mT-G17L+G20L^ +Cd | 14 | N/A | <0.0001 |  |  |  |  |  |
|  | PacL2^mT-K9A^ +Cd | 20 | N/A | 0.0303 |  |  |  |  |  |
|  |  |  |  |  |  |  |  |  |  |
| 7G | PacL2^WT^* | 13 | N/A |  |  |  |  |  |  |
|  | PacL2^G17L+G20L^ | 16 | <0.0001 |  |  |  |  |  |  |
|  | PacL2^K9A^ | 19 | 0.0052 |  |  |  |  |  |  |
|  |  |  |  |  |  |  |  |  |  |
| 7H | PacL2^WT^* | 13 | N/A |  |  |  |  |  |  |
|  | PacL2^G17L+G20L^ | 16 | <0.0001 |  |  |  |  |  |  |
|  | PacL2^K9A^ | 19 | <0.0001 |  |  |  |  |  |  |
|  |  |  |  |  |  |  |  |  |  |
| 7I | PacL2^WT^* | 158 | N/A |  |  |  |  |  |  |
|  | PacL2^G17L+G20L^ | 94 | <0.0001 |  |  |  |  |  |  |
|  | PacL2^K9A^ | 157 | 0.0011 |  |  |  |  |  |  |

**Table EV6. Exact p-values and biological replicate numbers for all figure panels.** * or **: reference strains used for the statistical test. n: number of biological replicates.
