# Supplementary figures and images for: Membrane-associated effluxosomes coordinate multi-metal resistance in Mycobacterium tuberculosis
Source: EMBO J. 2026 Feb 13;45(7):2306–37. doi: 10.1038/s44318-026-00715-1 (PMC13043812; doi:10.1038/s44318-026-00715-1)

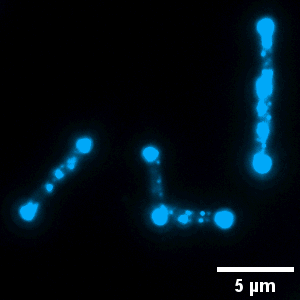

Supplement: Supplementary file 9 — Movie EV1 [file 44318_2026_715_MOESM9_ESM.zip › Movie EV1/Movie EV1.gif]

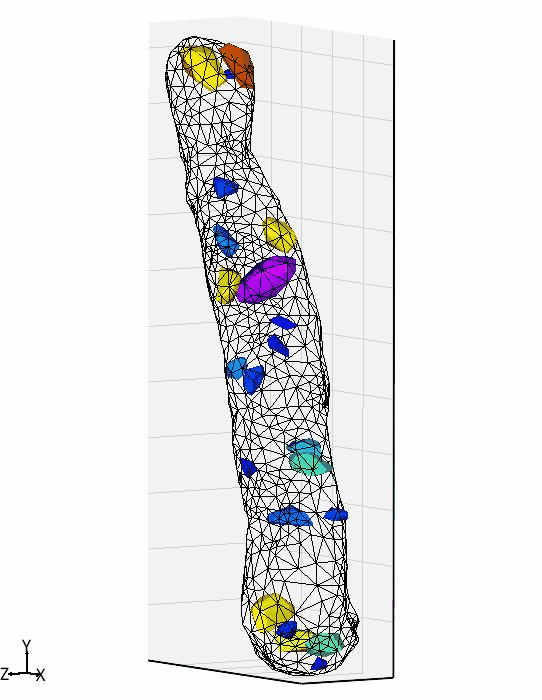

Supplement: Supplementary file 12 — Movie EV4 [file 44318_2026_715_MOESM12_ESM.zip › Movie EV4/Movie EV4.gif]

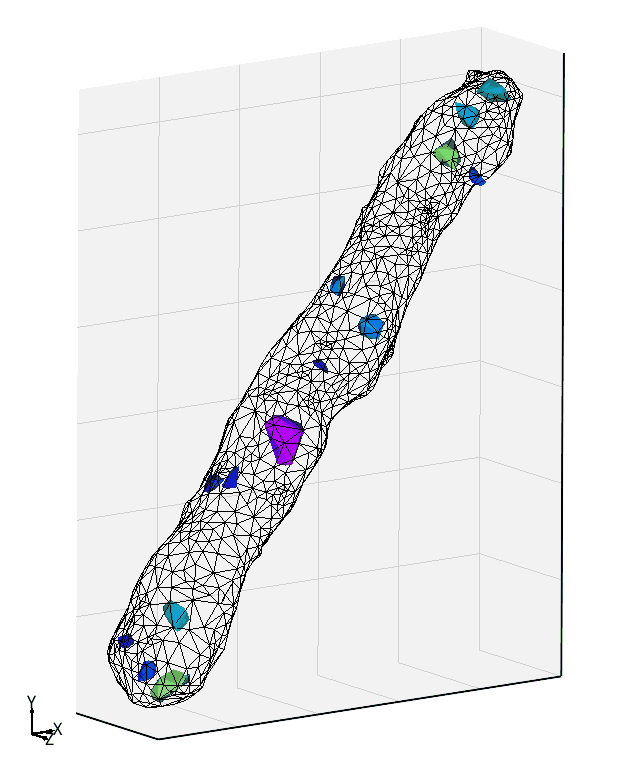

Supplement: Supplementary file 13 — Movie EV5 [file 44318_2026_715_MOESM13_ESM.zip › Movie EV5/Movie EV5.gif]

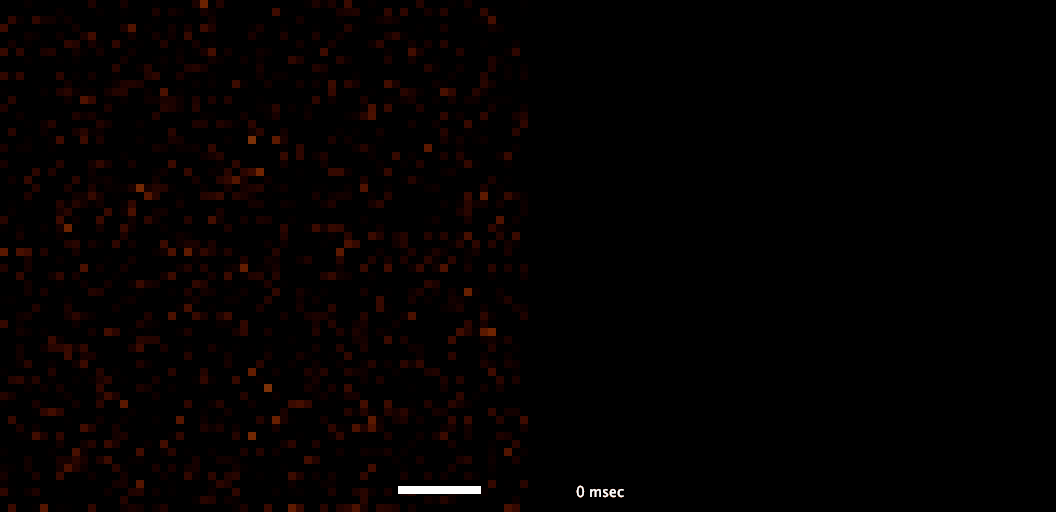

Supplement: Supplementary file 14 — Movie EV6 [file 44318_2026_715_MOESM14_ESM.zip › Movie EV6/Movie EV6.gif]

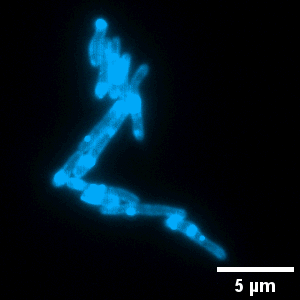

Supplement: Supplementary file 15 — Movie EV7 [file 44318_2026_715_MOESM15_ESM.zip › Movie EV7/Movie EV7.gif]

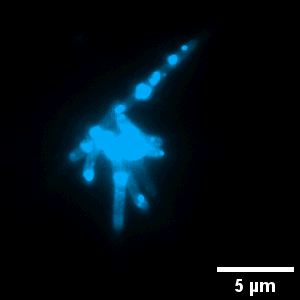

Supplement: Supplementary file 16 — Movie EV8 [file 44318_2026_715_MOESM16_ESM.zip › Movie EV8/MovieEV8.gif]

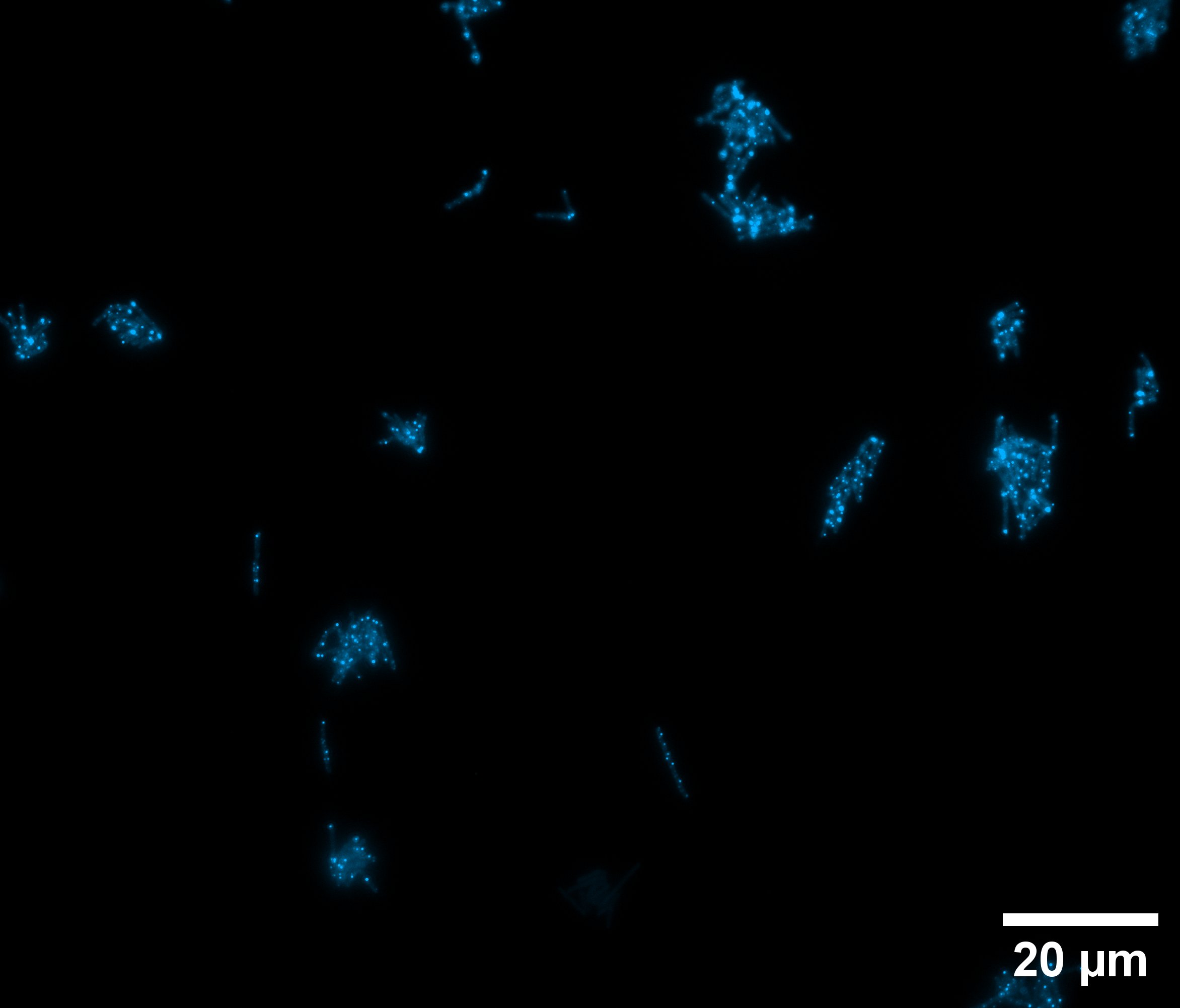

Supplement: Supplementary file 18 — Source data Fig. 2 [file 44318_2026_715_MOESM18_ESM.zip › Figure 2/Figure 2D/Figure 2D bottom left.jpg]

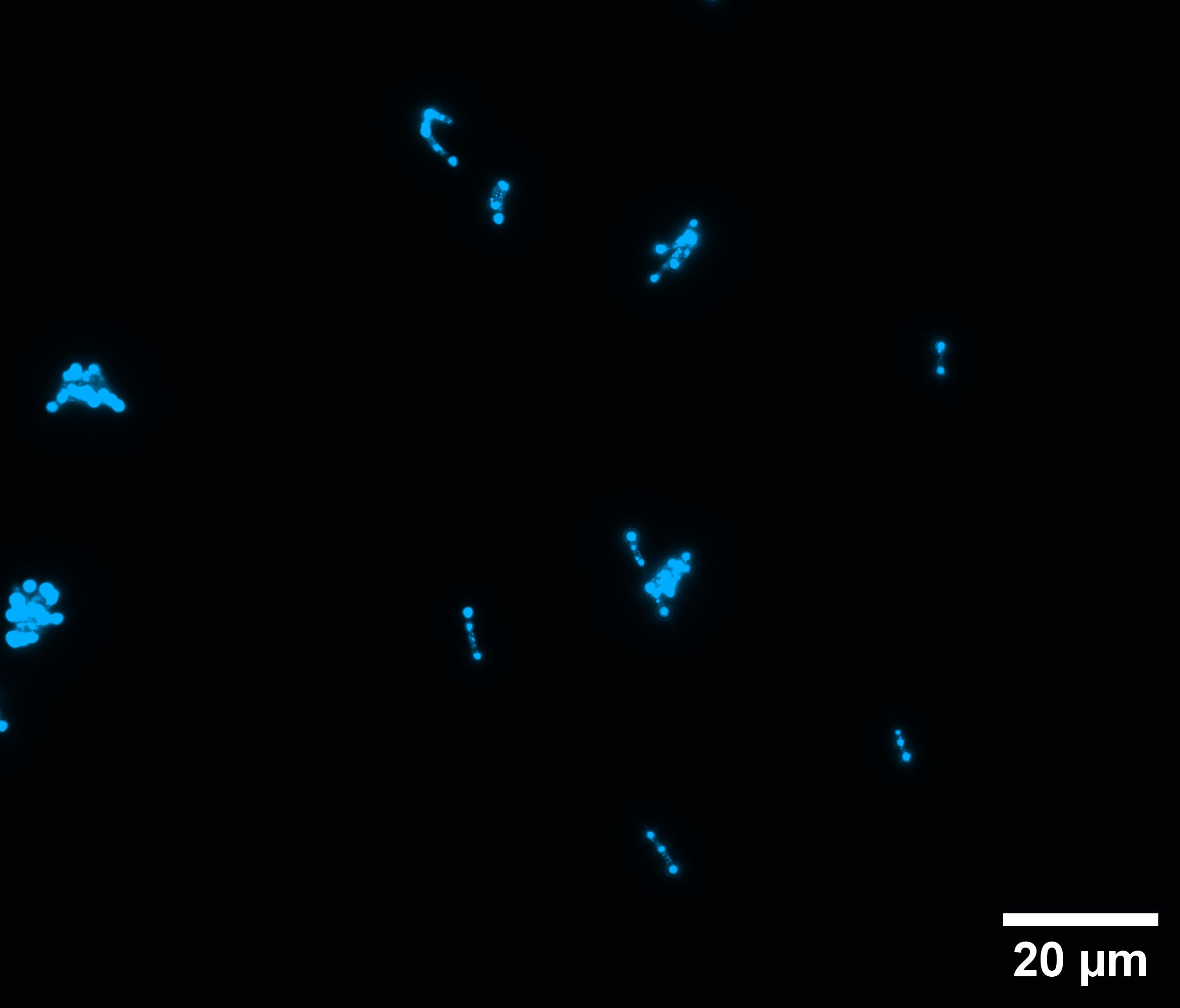

Supplement: Supplementary file 18 — Source data Fig. 2 [file 44318_2026_715_MOESM18_ESM.zip › Figure 2/Figure 2D/Figure 2D bottom right.jpg]

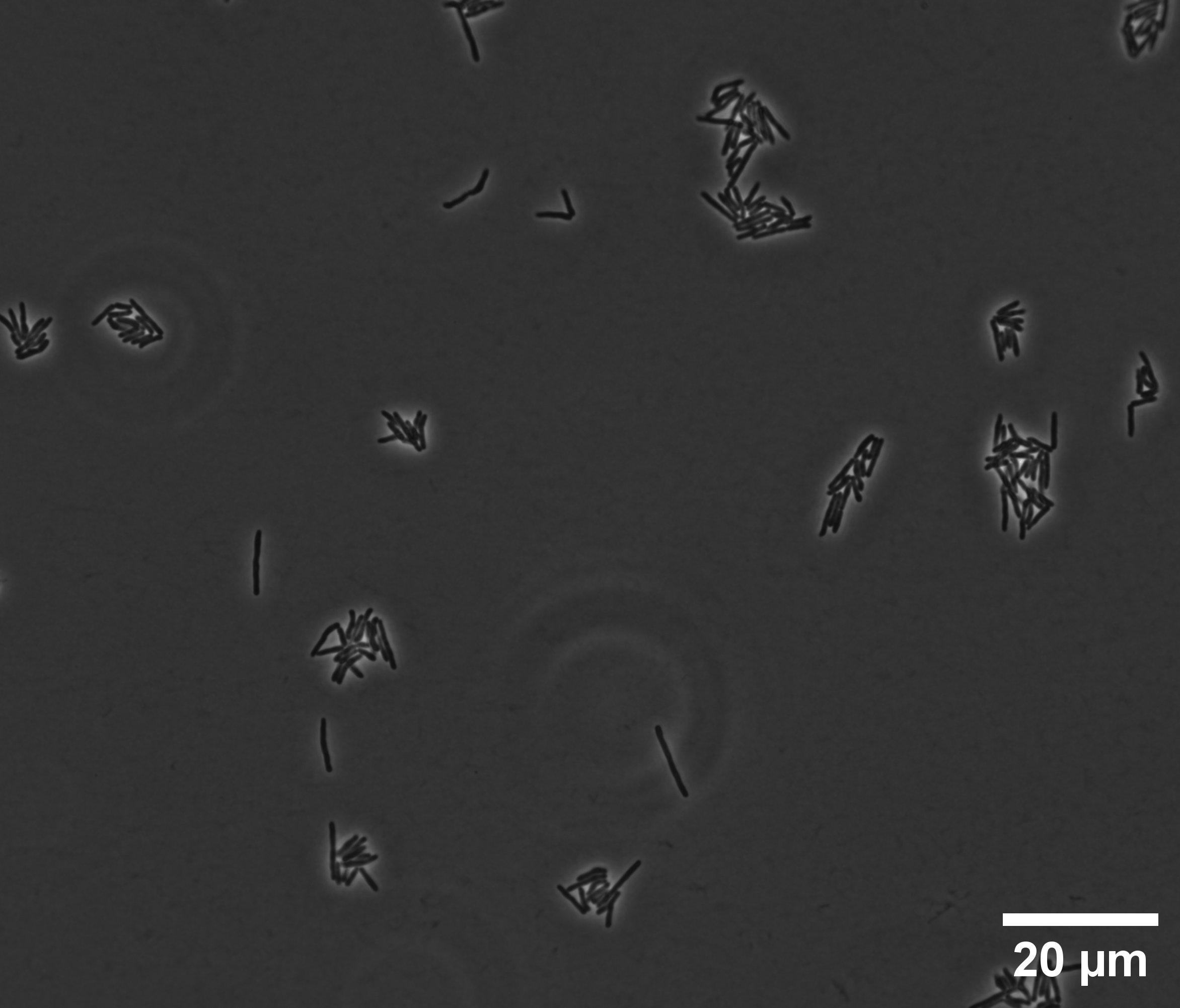

Supplement: Supplementary file 18 — Source data Fig. 2 [file 44318_2026_715_MOESM18_ESM.zip › Figure 2/Figure 2D/Figure 2D top left.jpg]

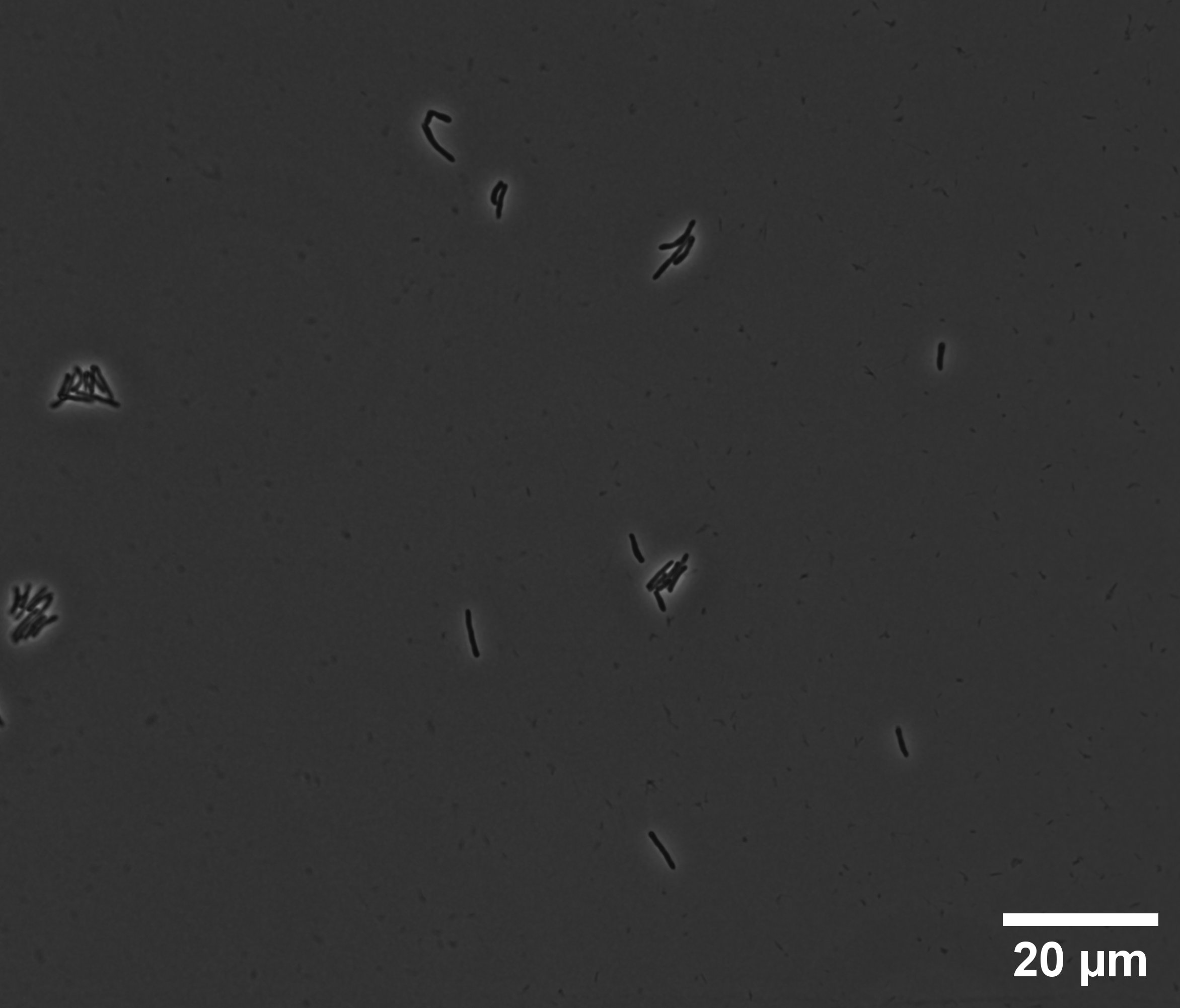

Supplement: Supplementary file 18 — Source data Fig. 2 [file 44318_2026_715_MOESM18_ESM.zip › Figure 2/Figure 2D/Figure 2D top right.jpg]

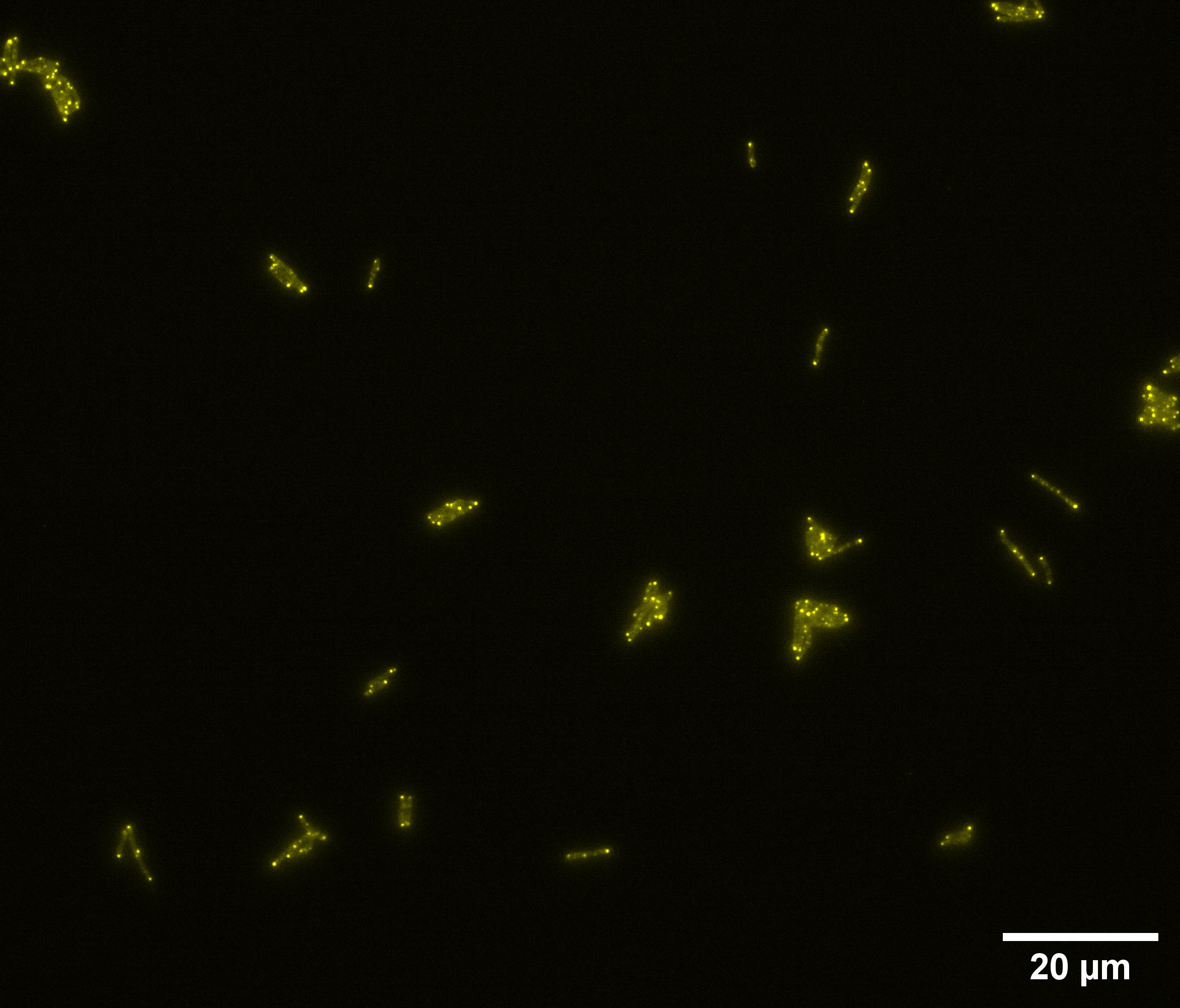

Supplement: Supplementary file 18 — Source data Fig. 2 [file 44318_2026_715_MOESM18_ESM.zip › Figure 2/Figure 2F/Figure 2F bottom left.jpg]

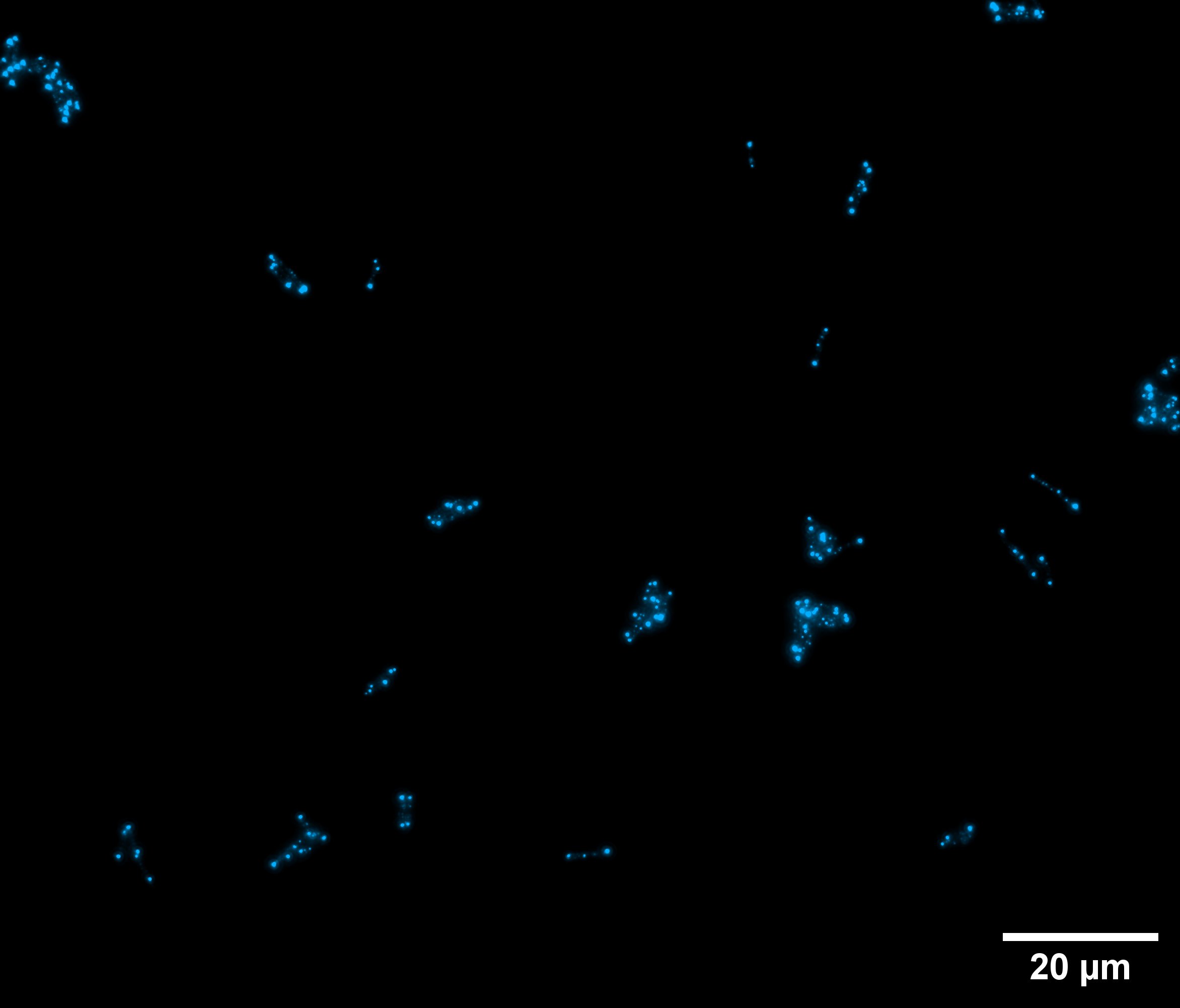

Supplement: Supplementary file 18 — Source data Fig. 2 [file 44318_2026_715_MOESM18_ESM.zip › Figure 2/Figure 2F/Figure 2F top left.jpg]

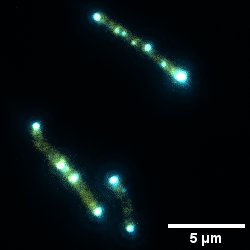

Supplement: Supplementary file 18 — Source data Fig. 2 [file 44318_2026_715_MOESM18_ESM.zip › Figure 2/Figure 2F/Figure 2F top right.jpg]

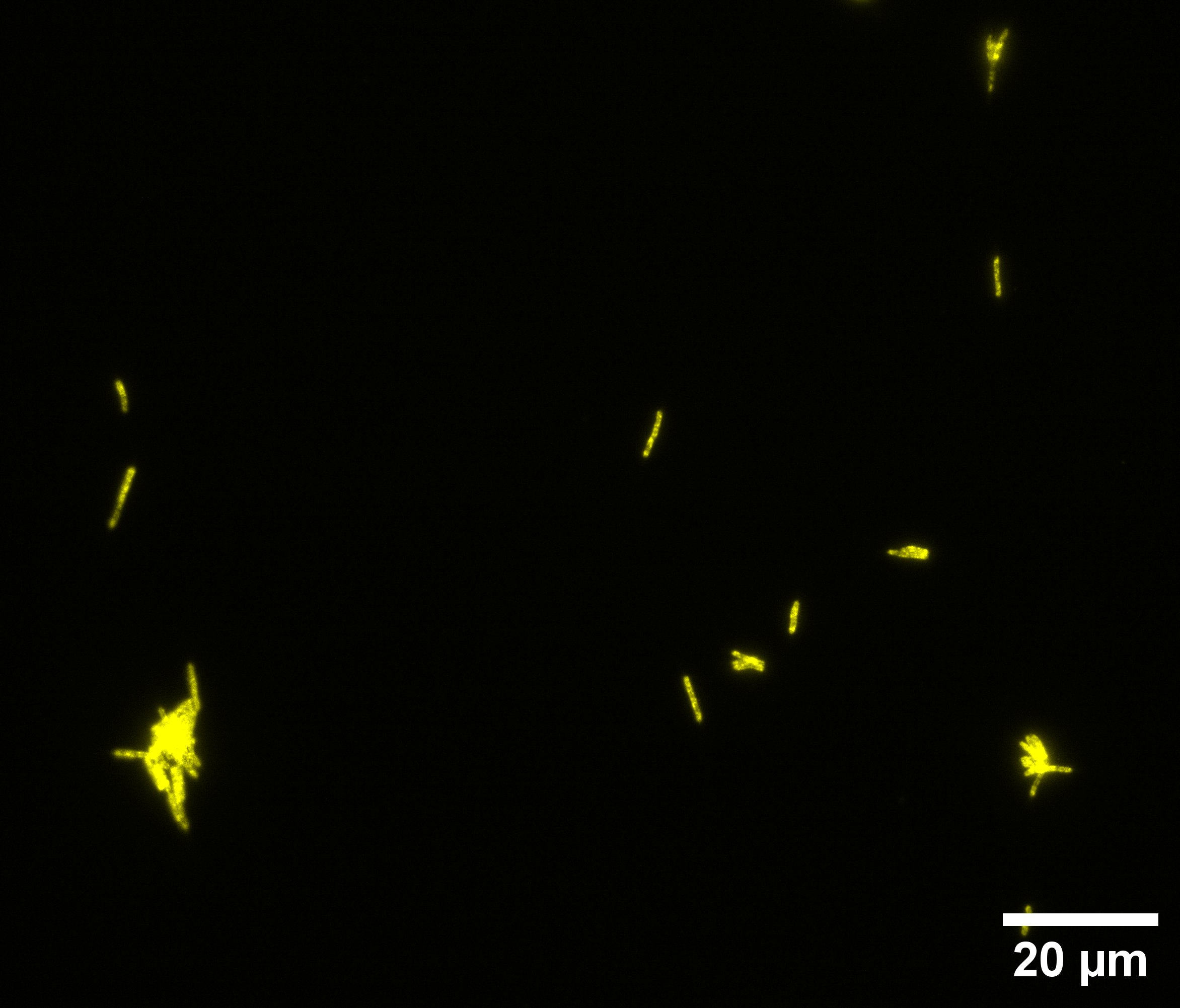

Supplement: Supplementary file 18 — Source data Fig. 2 [file 44318_2026_715_MOESM18_ESM.zip › Figure 2/Figure 2G/Figure 2G bottom left.jpg]

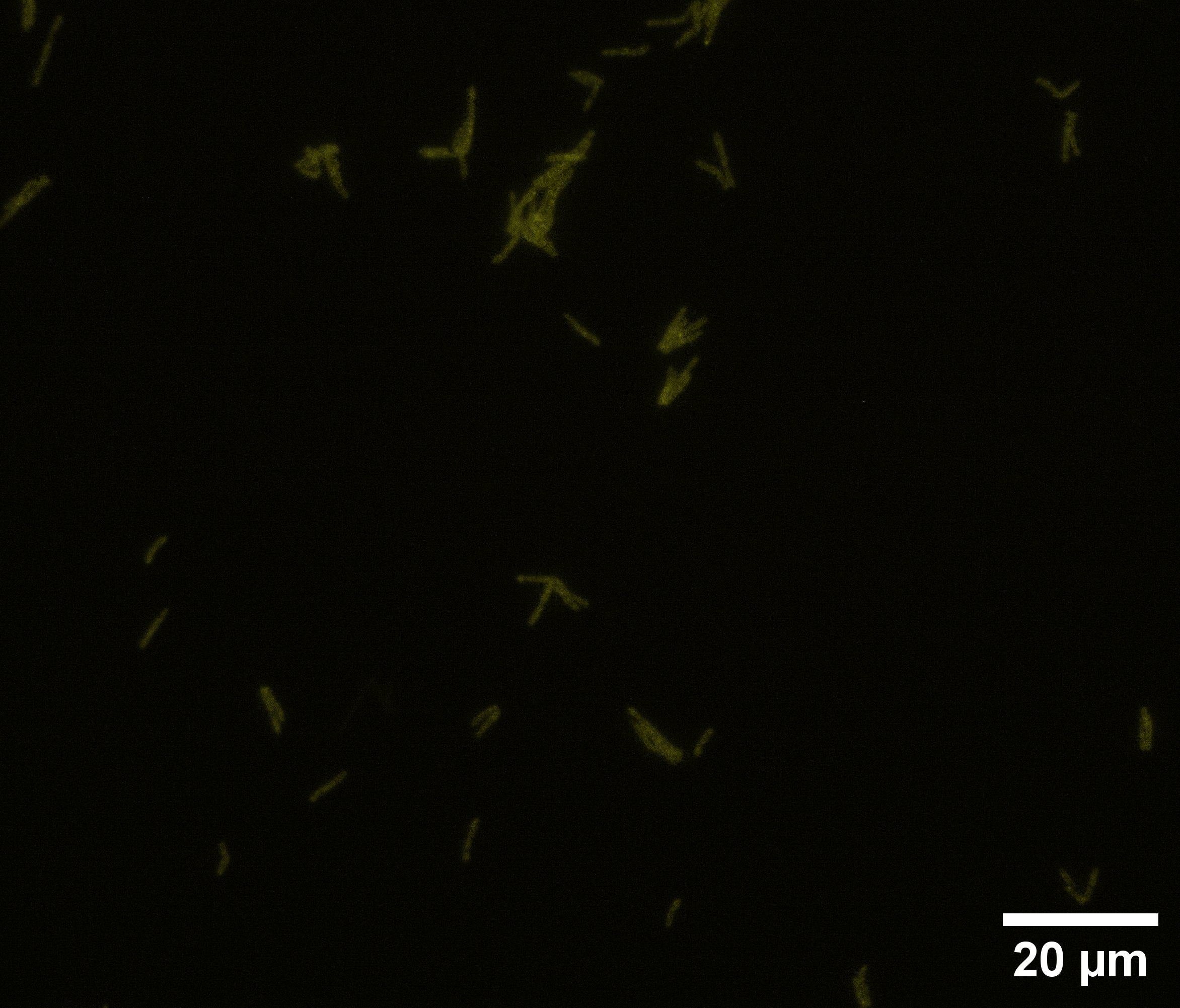

Supplement: Supplementary file 18 — Source data Fig. 2 [file 44318_2026_715_MOESM18_ESM.zip › Figure 2/Figure 2G/Figure 2G bottom right.jpg]

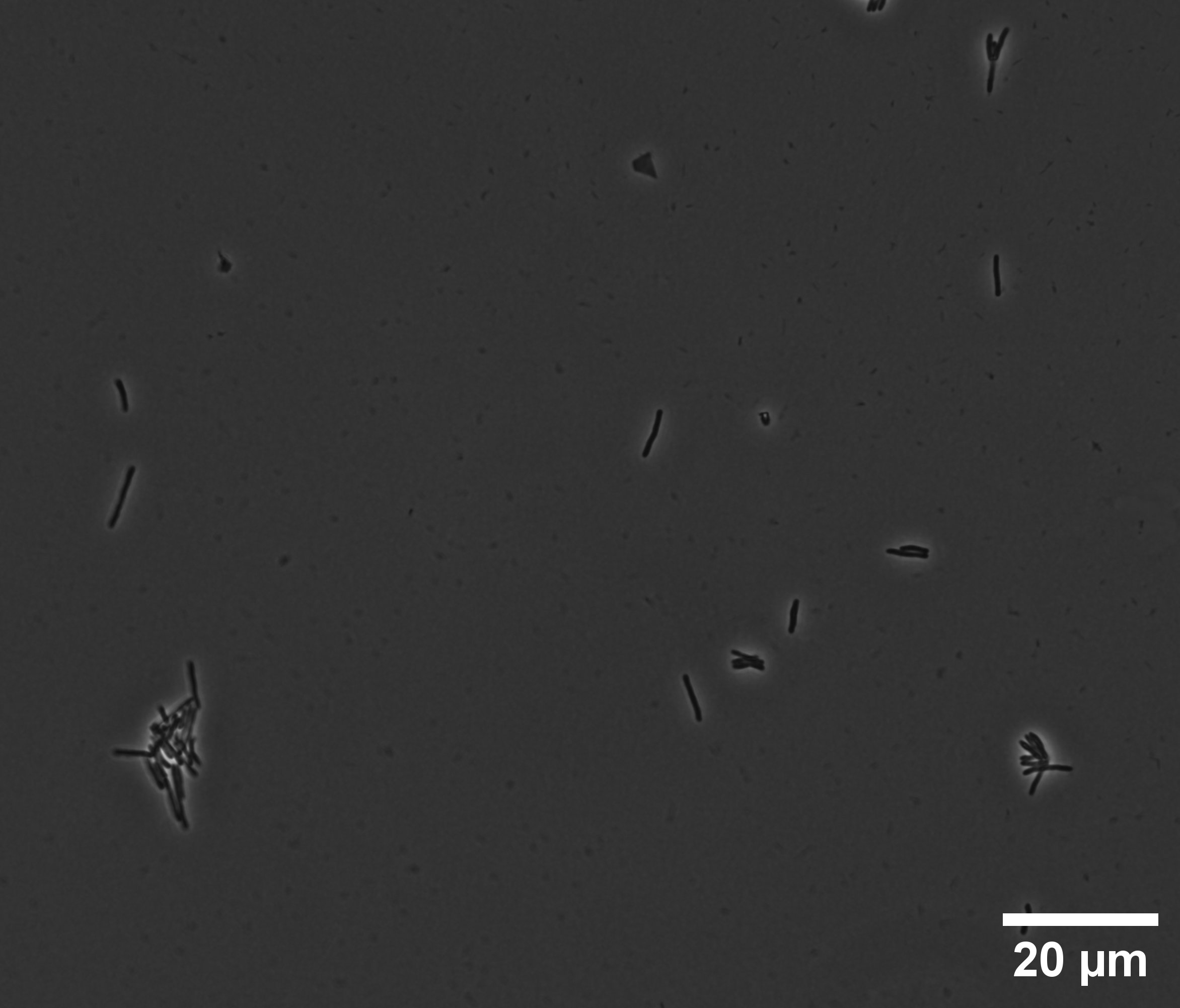

Supplement: Supplementary file 18 — Source data Fig. 2 [file 44318_2026_715_MOESM18_ESM.zip › Figure 2/Figure 2G/Figure 2G top left.jpg]

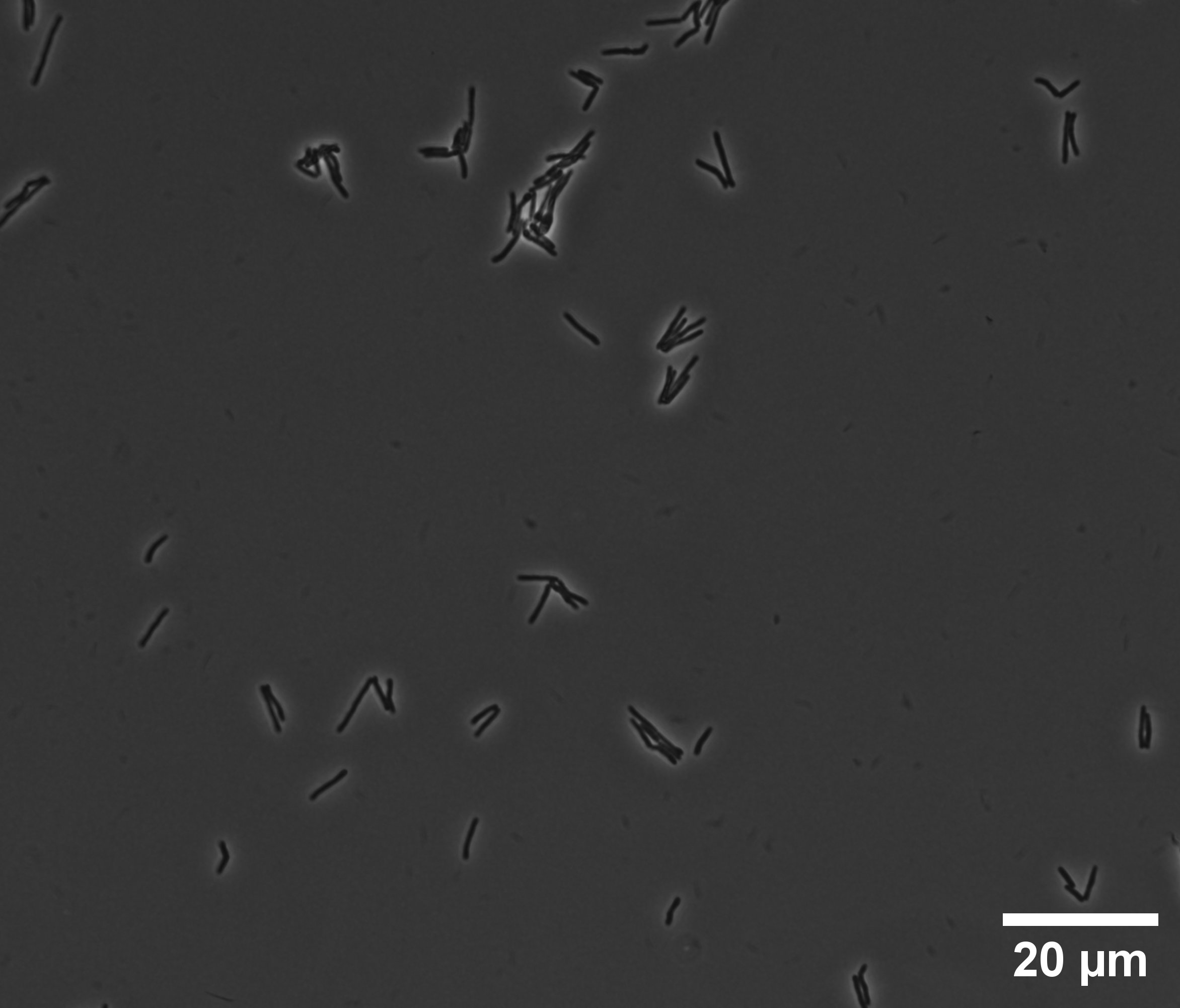

Supplement: Supplementary file 18 — Source data Fig. 2 [file 44318_2026_715_MOESM18_ESM.zip › Figure 2/Figure 2G/Figure 2G top right.jpg]

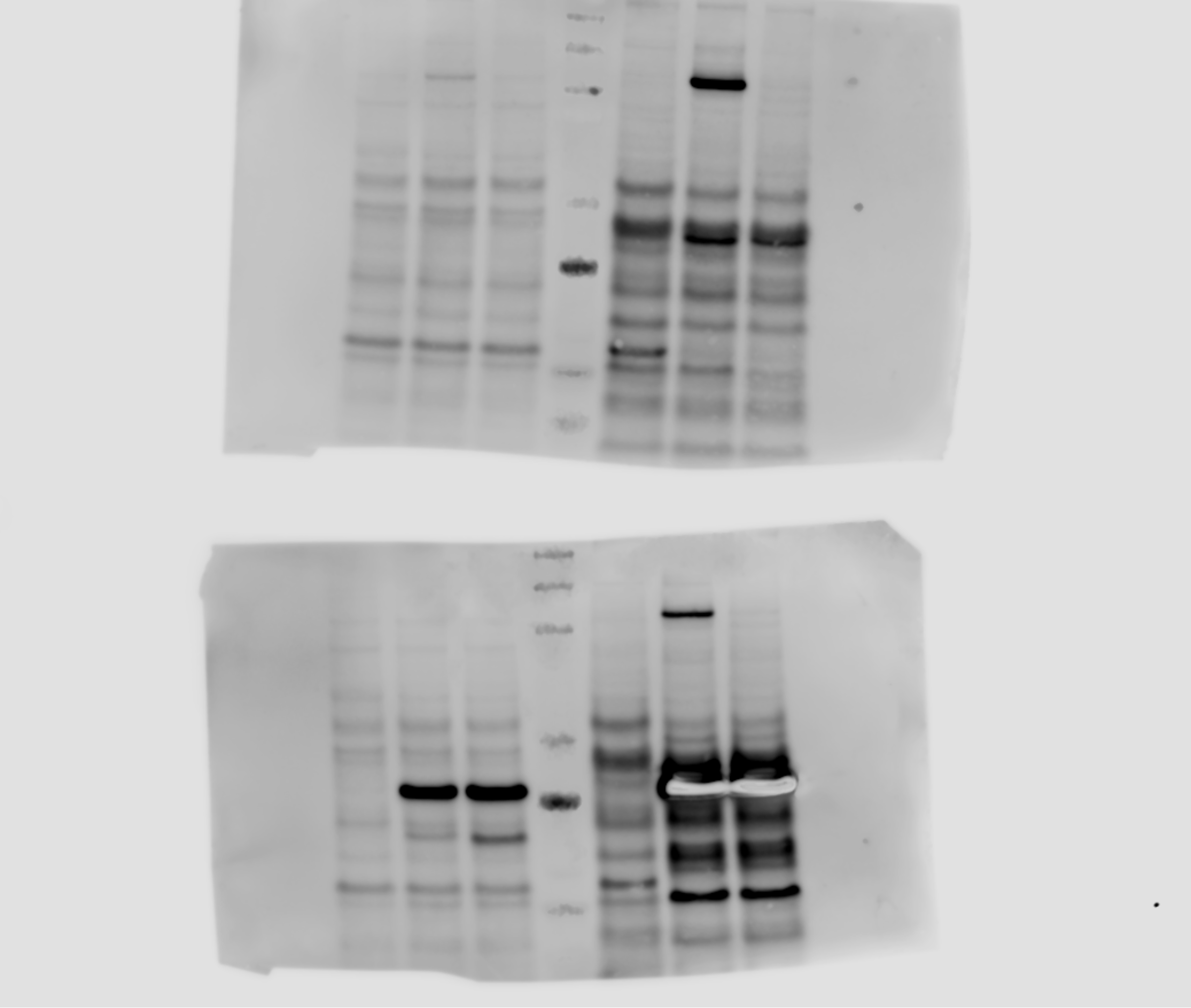

Supplement: Supplementary file 18 — Source data Fig. 2 [file 44318_2026_715_MOESM18_ESM.zip › Figure 2/Figure 2I/Figure 2I.tif]

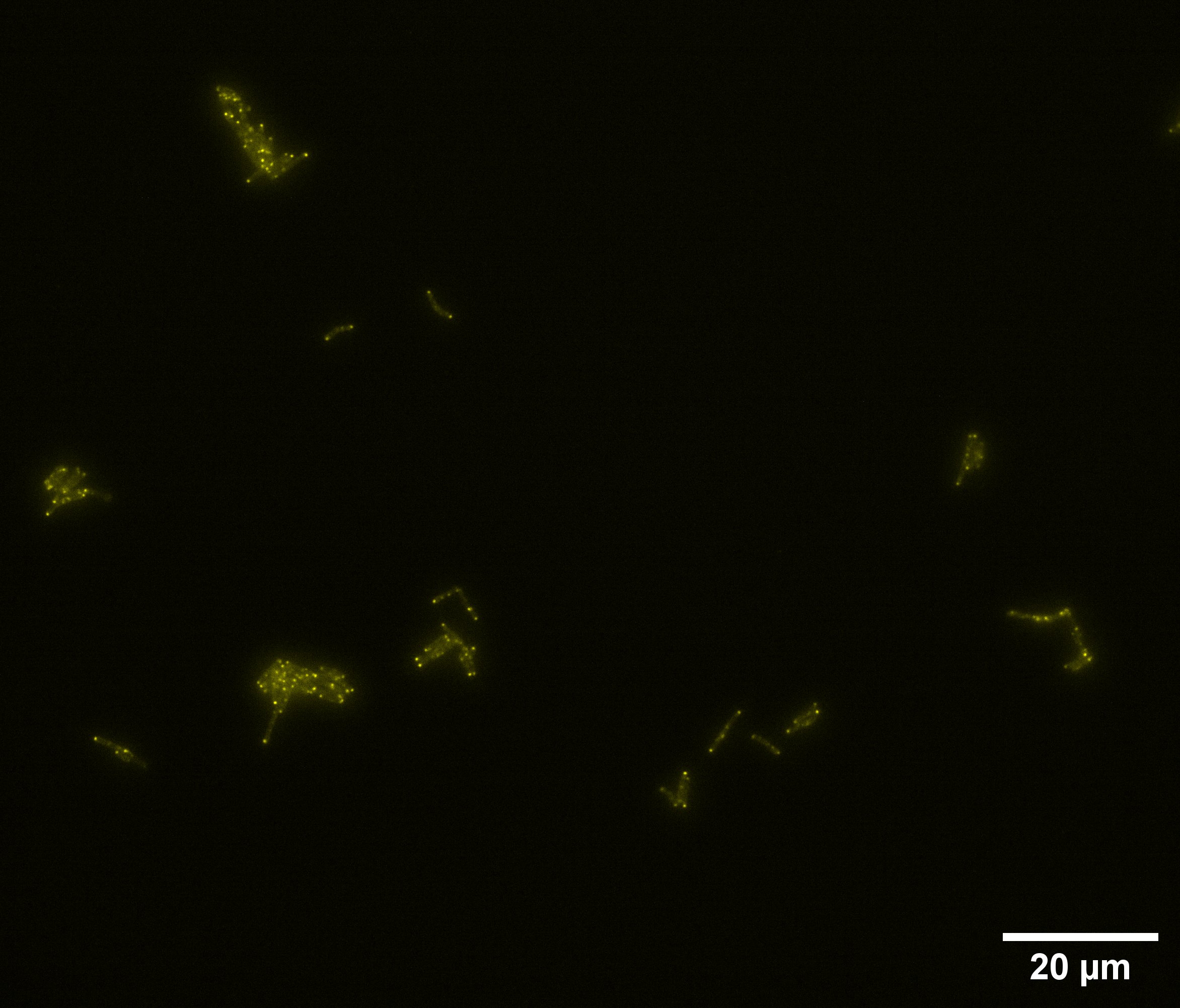

Supplement: Supplementary file 18 — Source data Fig. 2 [file 44318_2026_715_MOESM18_ESM.zip › Figure 2/Figure 2K/Figure 2K bottom left.jpg]

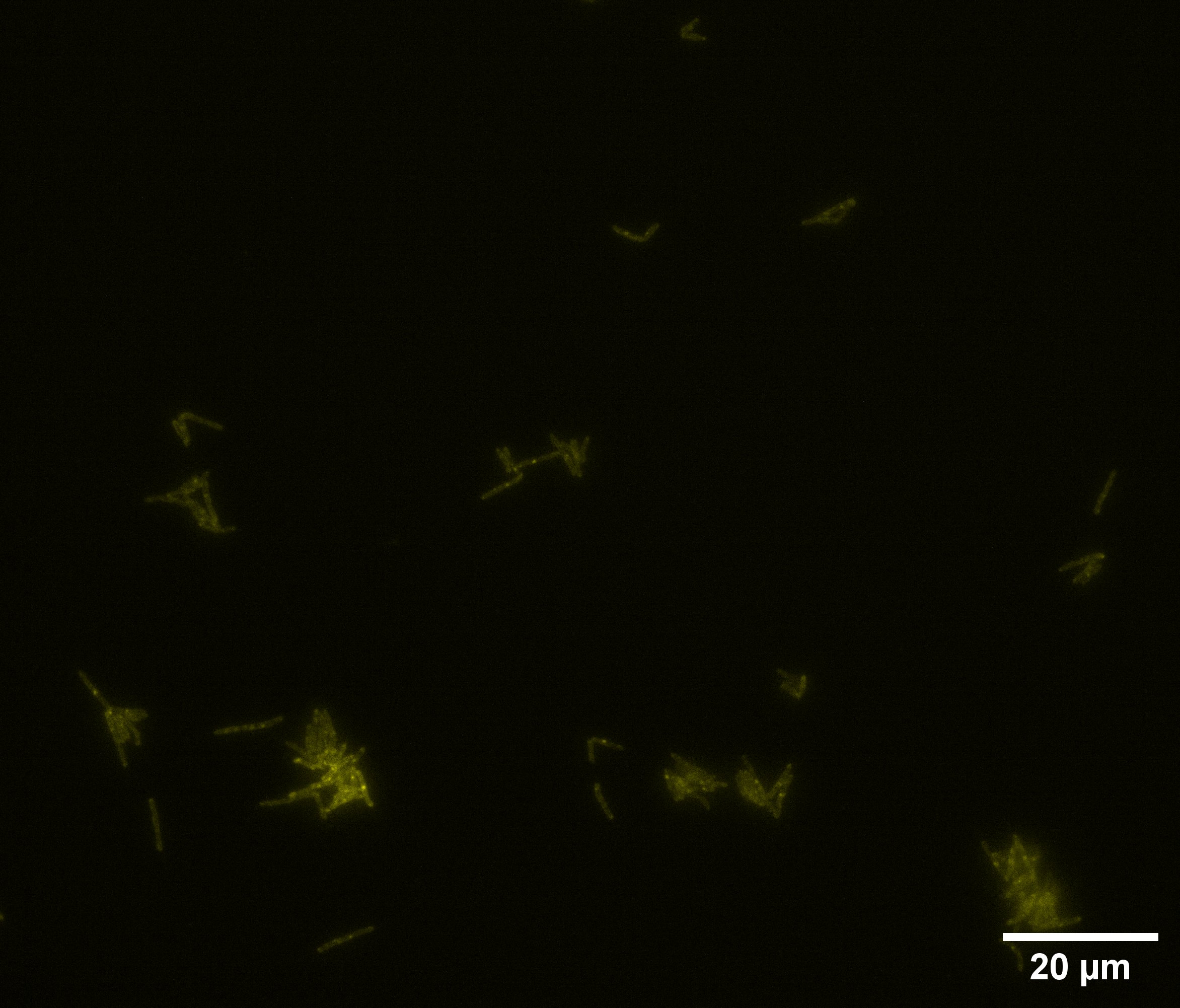

Supplement: Supplementary file 18 — Source data Fig. 2 [file 44318_2026_715_MOESM18_ESM.zip › Figure 2/Figure 2K/Figure 2K bottom middle.jpg]

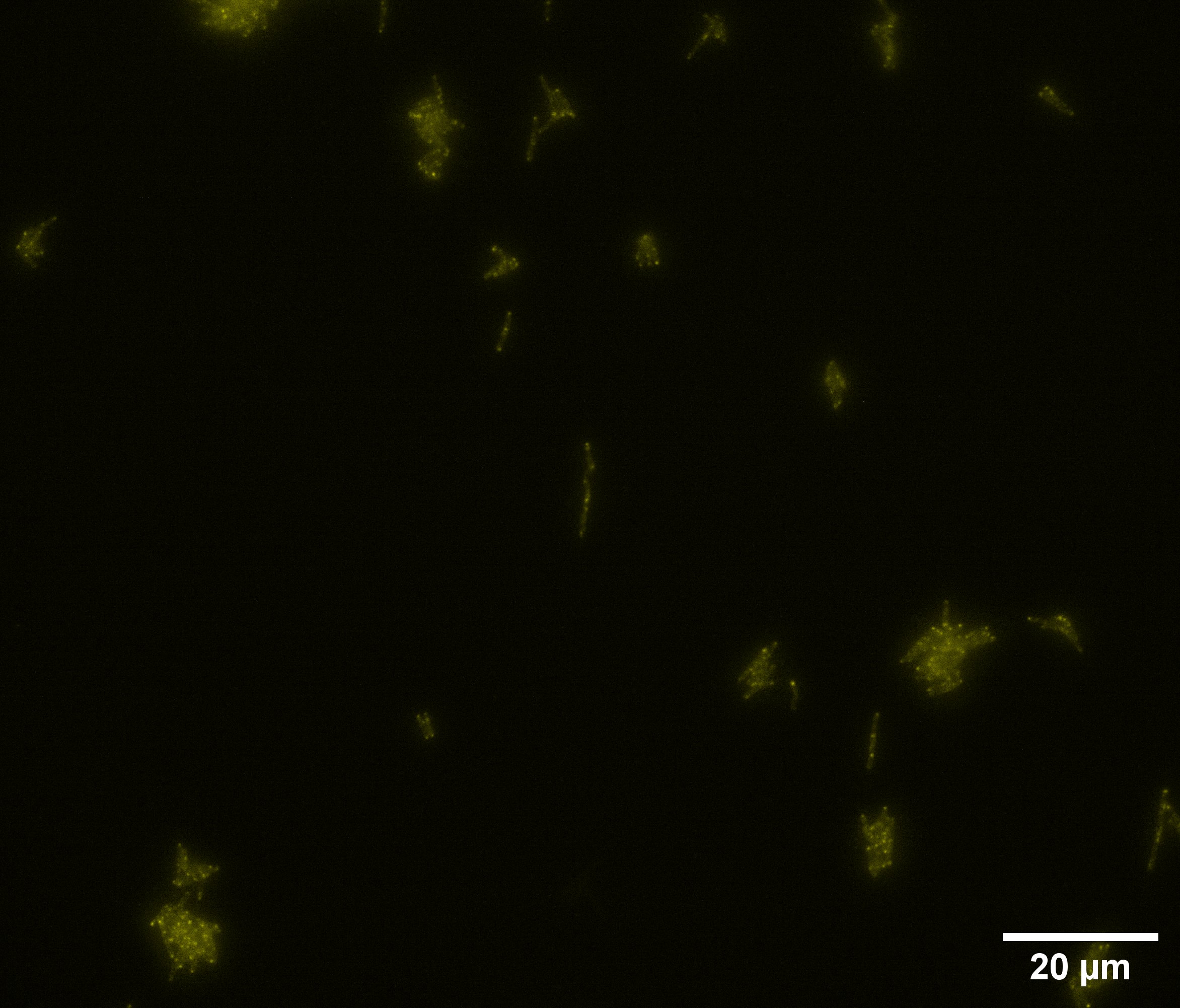

Supplement: Supplementary file 18 — Source data Fig. 2 [file 44318_2026_715_MOESM18_ESM.zip › Figure 2/Figure 2K/Figure 2K bottom right.jpg]

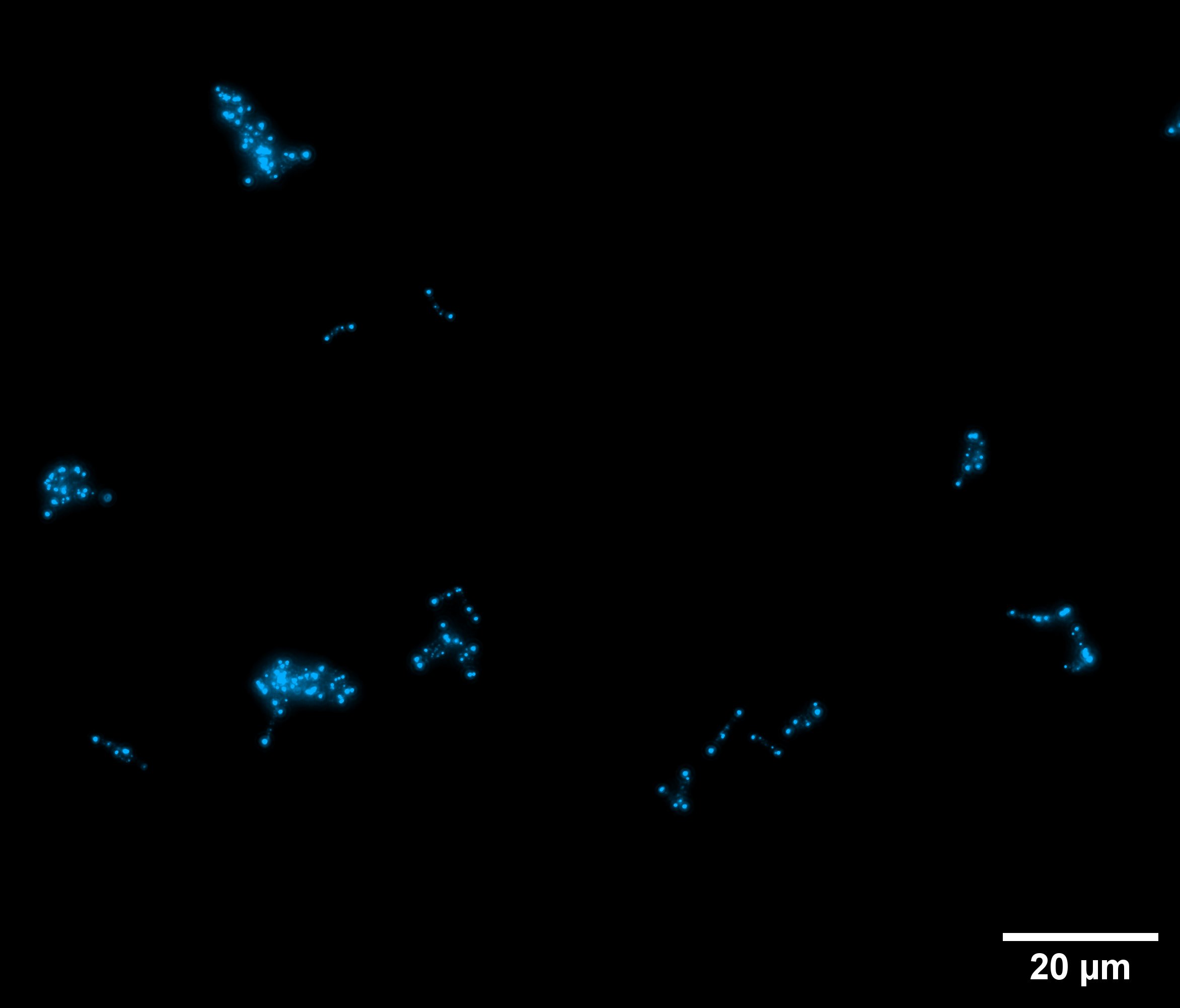

Supplement: Supplementary file 18 — Source data Fig. 2 [file 44318_2026_715_MOESM18_ESM.zip › Figure 2/Figure 2K/Figure 2K top left.jpg]

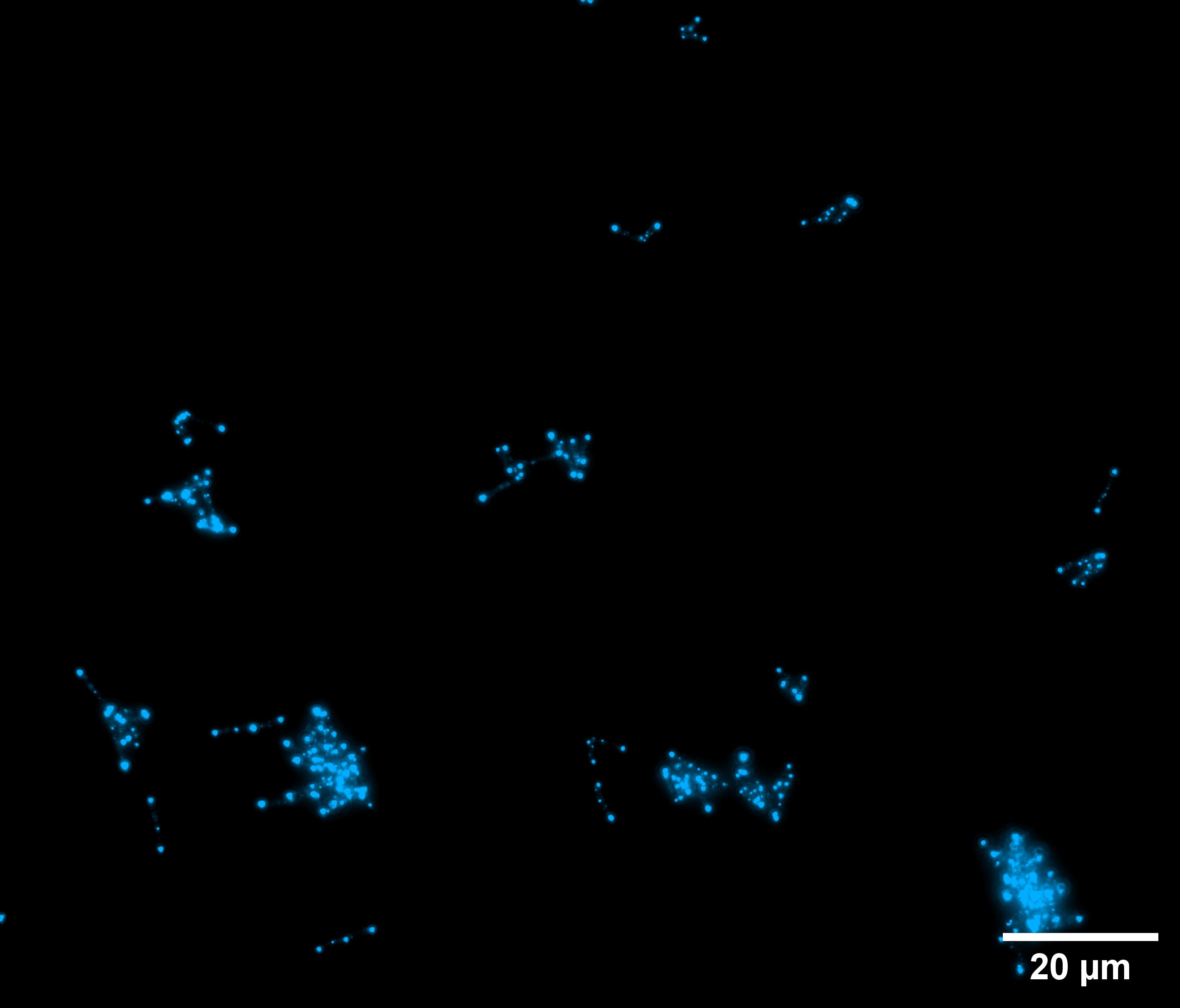

Supplement: Supplementary file 18 — Source data Fig. 2 [file 44318_2026_715_MOESM18_ESM.zip › Figure 2/Figure 2K/Figure 2K top middle.jpg]

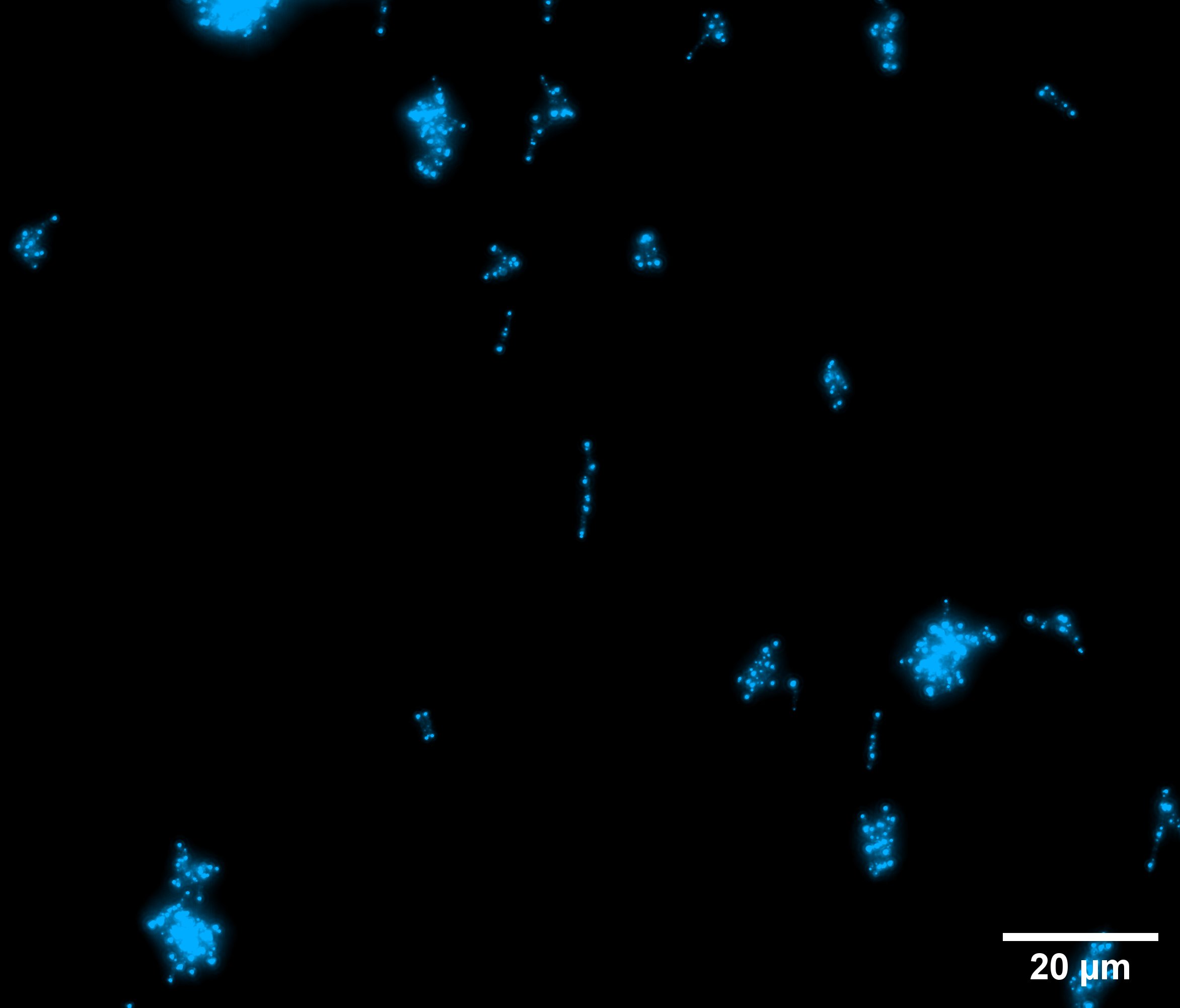

Supplement: Supplementary file 18 — Source data Fig. 2 [file 44318_2026_715_MOESM18_ESM.zip › Figure 2/Figure 2K/Figure 2K top right.jpg]

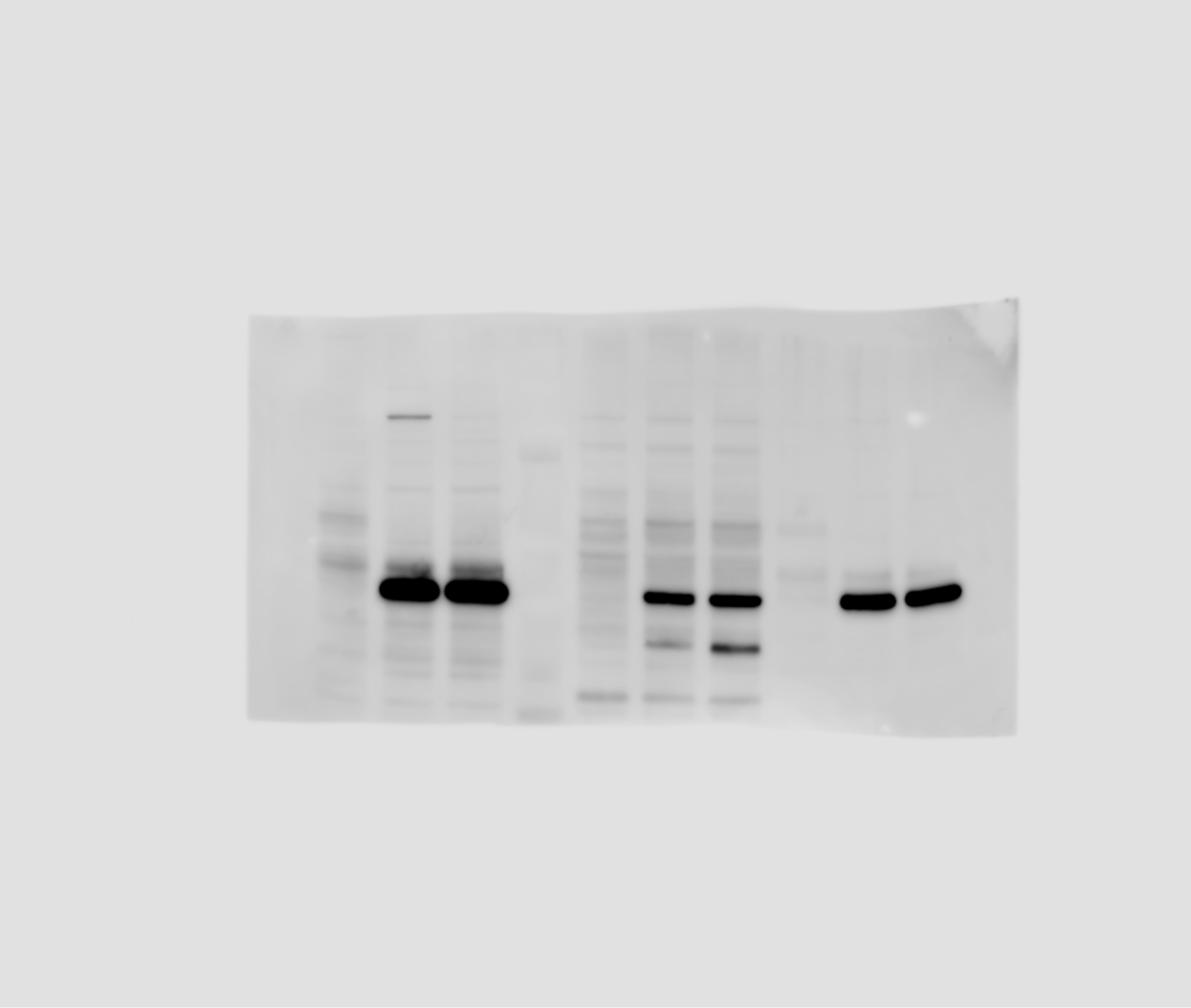

Supplement: Supplementary file 18 — Source data Fig. 2 [file 44318_2026_715_MOESM18_ESM.zip › Figure 2/Figure 2N/Figure 2N.tif]

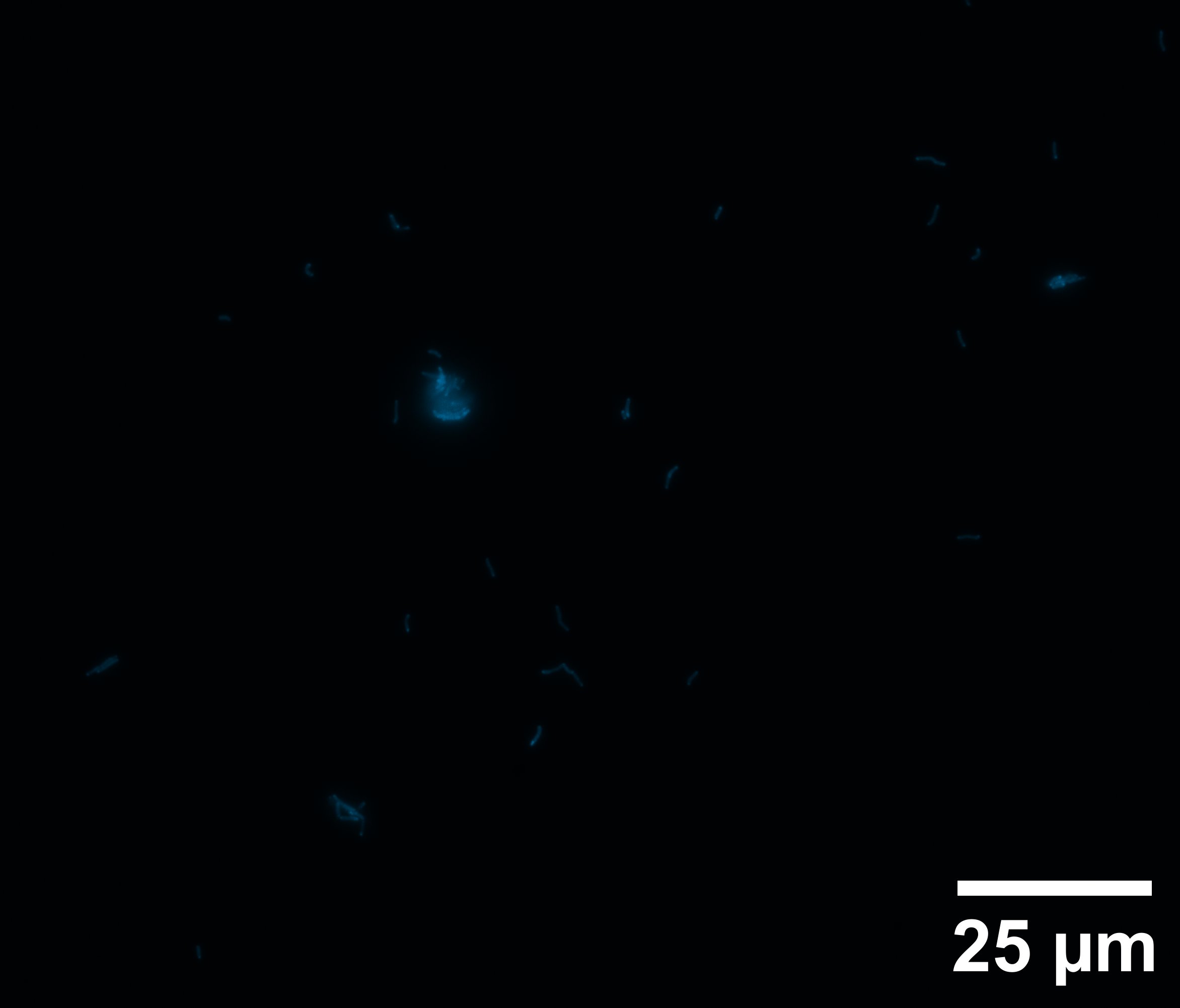

Supplement: Supplementary file 20 — Source data Fig. 4 [file 44318_2026_715_MOESM20_ESM.zip › Figure 4/Figure 4B/Figure 4B bottom left.jpg]

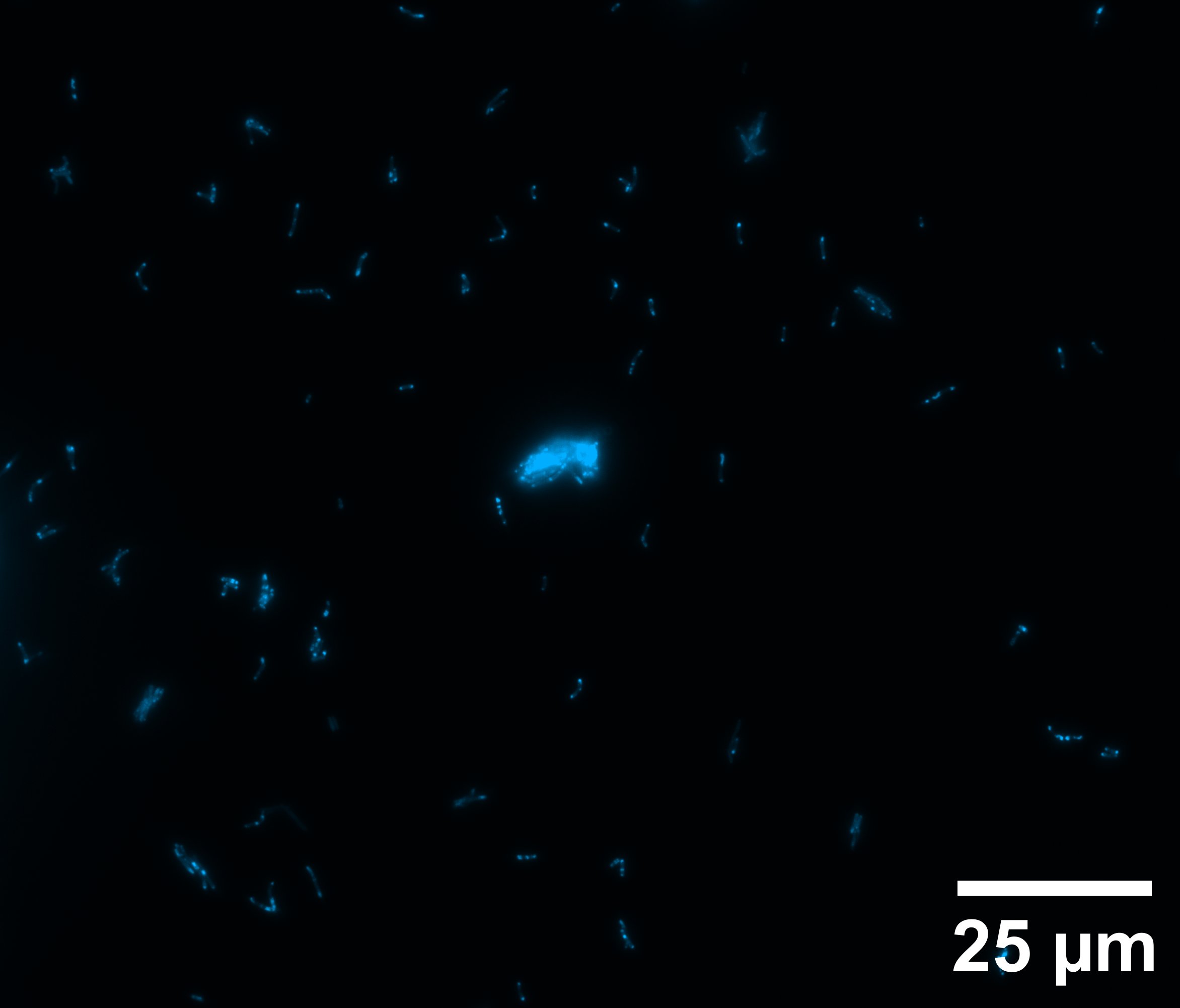

Supplement: Supplementary file 20 — Source data Fig. 4 [file 44318_2026_715_MOESM20_ESM.zip › Figure 4/Figure 4B/Figure 4B bottom middle left.jpg]

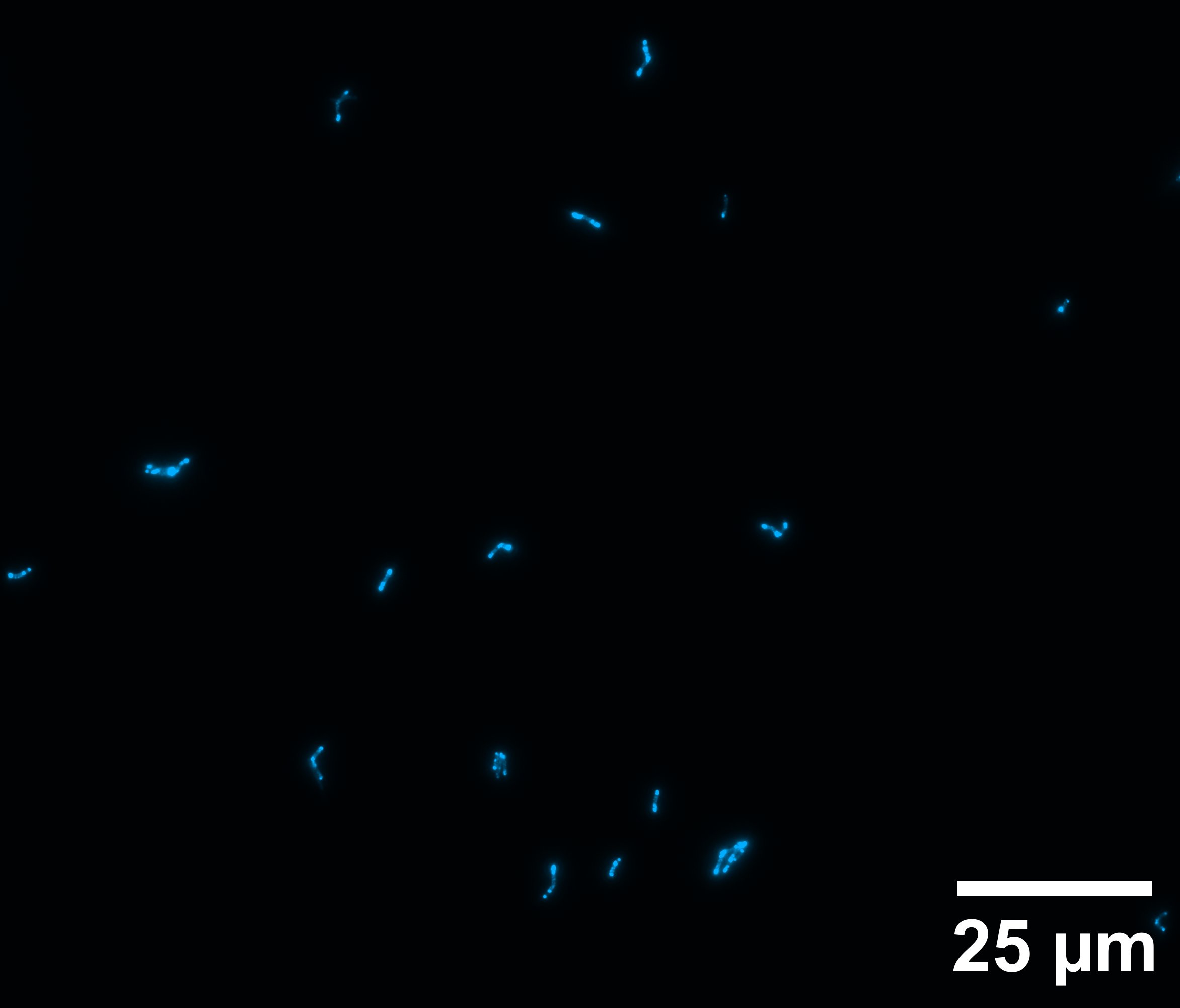

Supplement: Supplementary file 20 — Source data Fig. 4 [file 44318_2026_715_MOESM20_ESM.zip › Figure 4/Figure 4B/Figure 4B bottom middle right.jpg]

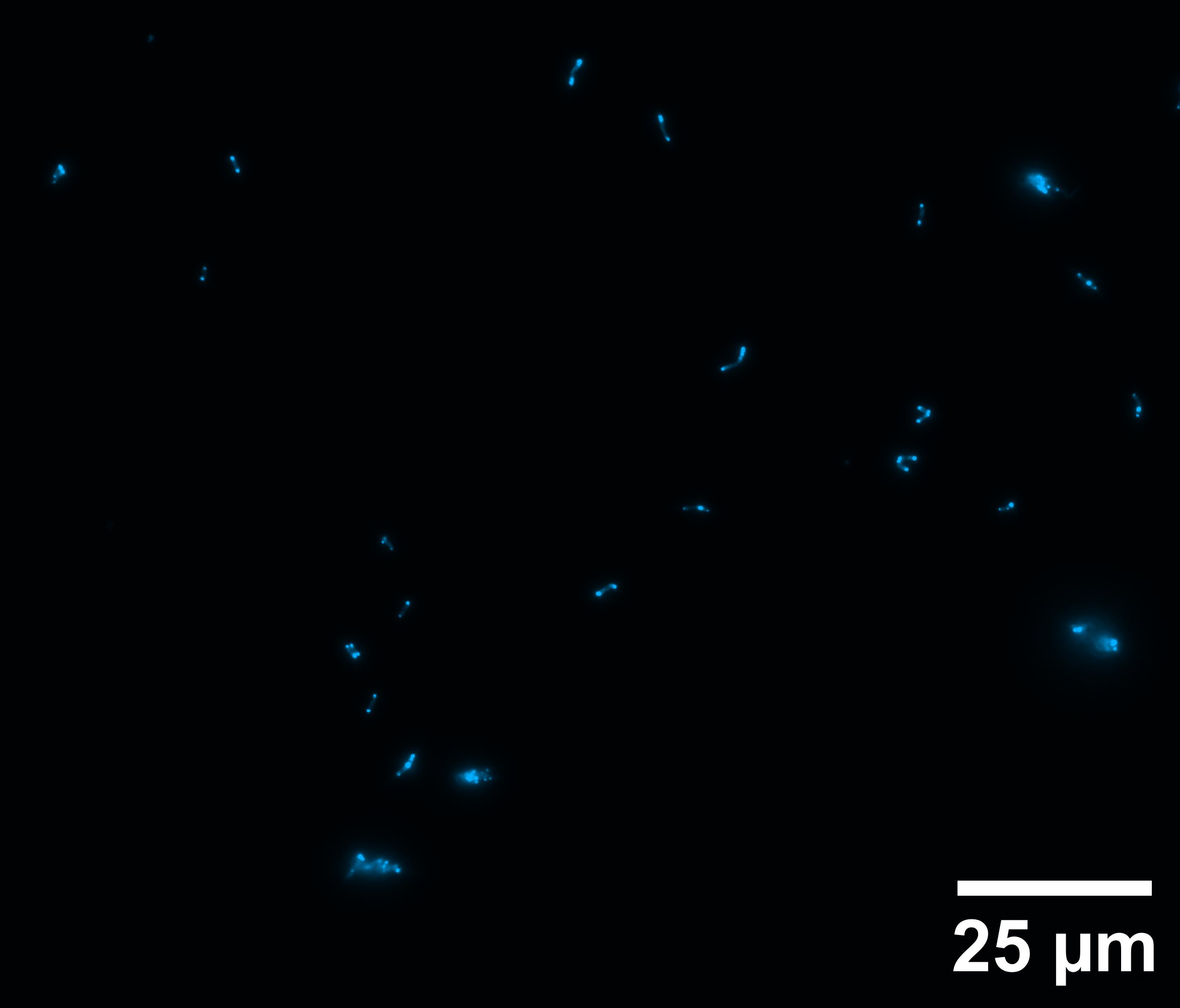

Supplement: Supplementary file 20 — Source data Fig. 4 [file 44318_2026_715_MOESM20_ESM.zip › Figure 4/Figure 4B/Figure 4B bottom right.jpg]

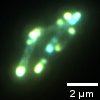

Supplement: Supplementary file 20 — Source data Fig. 4 [file 44318_2026_715_MOESM20_ESM.zip › Figure 4/Figure 4B/Figure 4B composite.jpg]

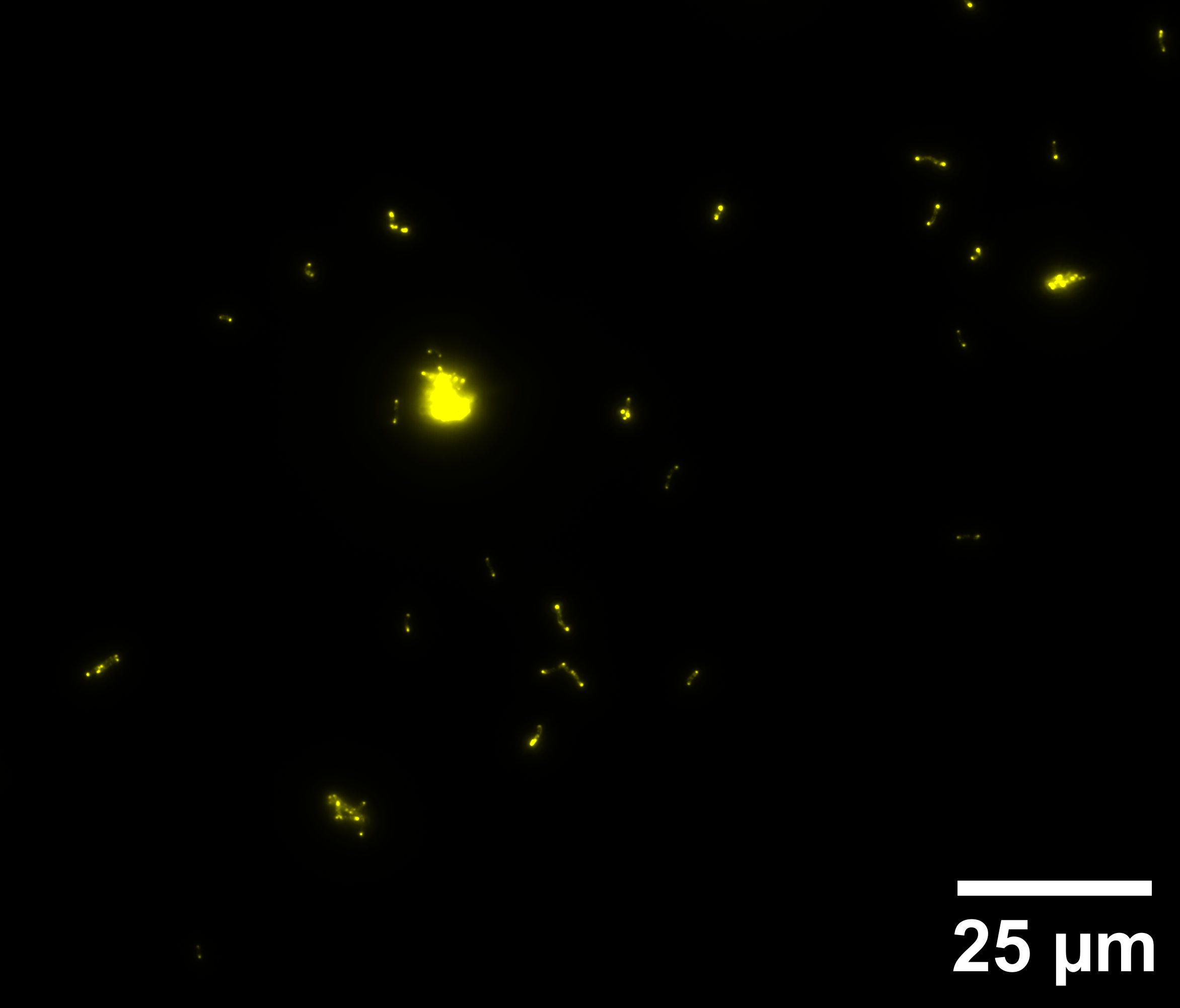

Supplement: Supplementary file 20 — Source data Fig. 4 [file 44318_2026_715_MOESM20_ESM.zip › Figure 4/Figure 4B/Figure 4B top left.jpg]

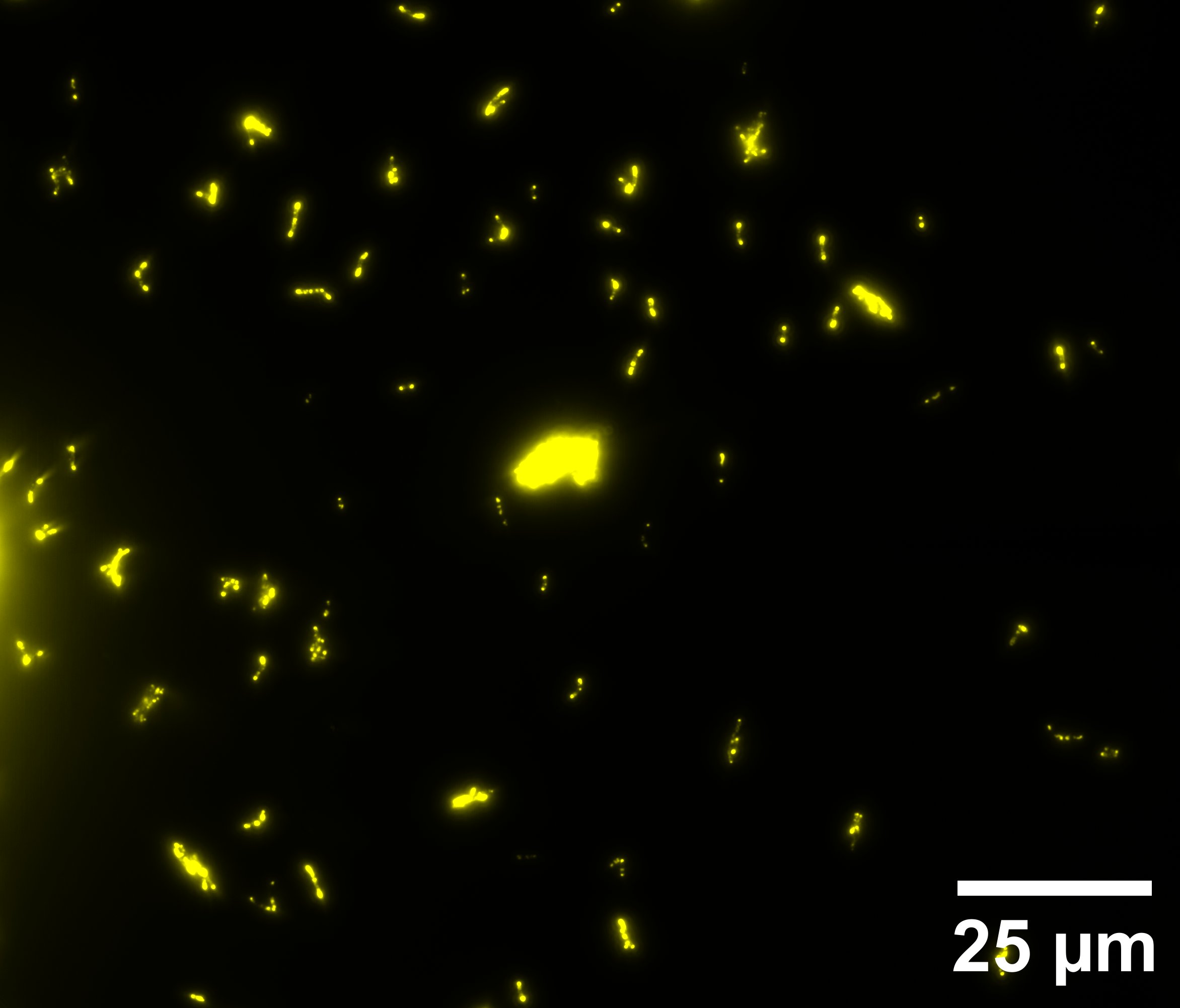

Supplement: Supplementary file 20 — Source data Fig. 4 [file 44318_2026_715_MOESM20_ESM.zip › Figure 4/Figure 4B/Figure 4B top middle left.jpg]

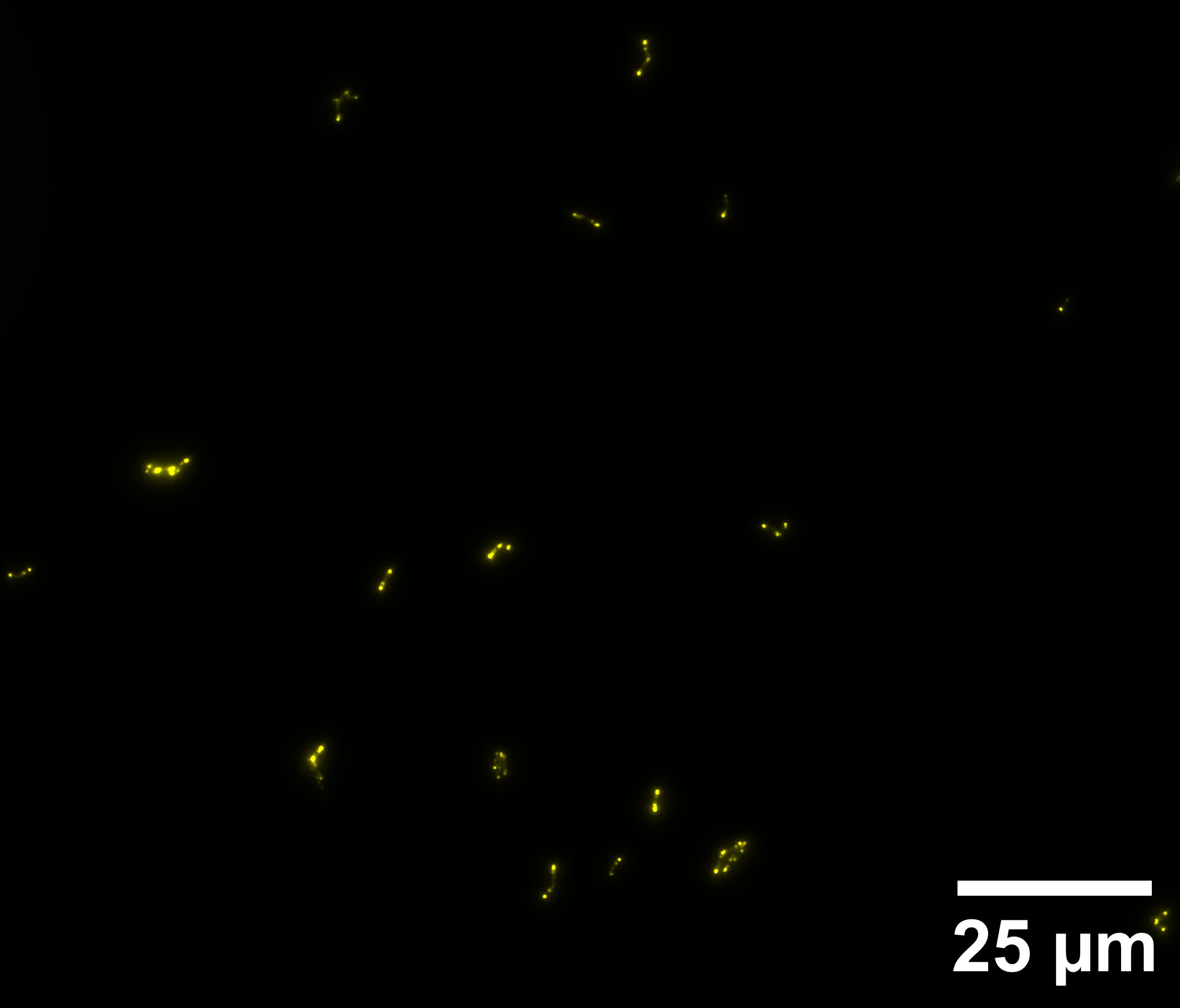

Supplement: Supplementary file 20 — Source data Fig. 4 [file 44318_2026_715_MOESM20_ESM.zip › Figure 4/Figure 4B/Figure 4B top middle right.jpg]

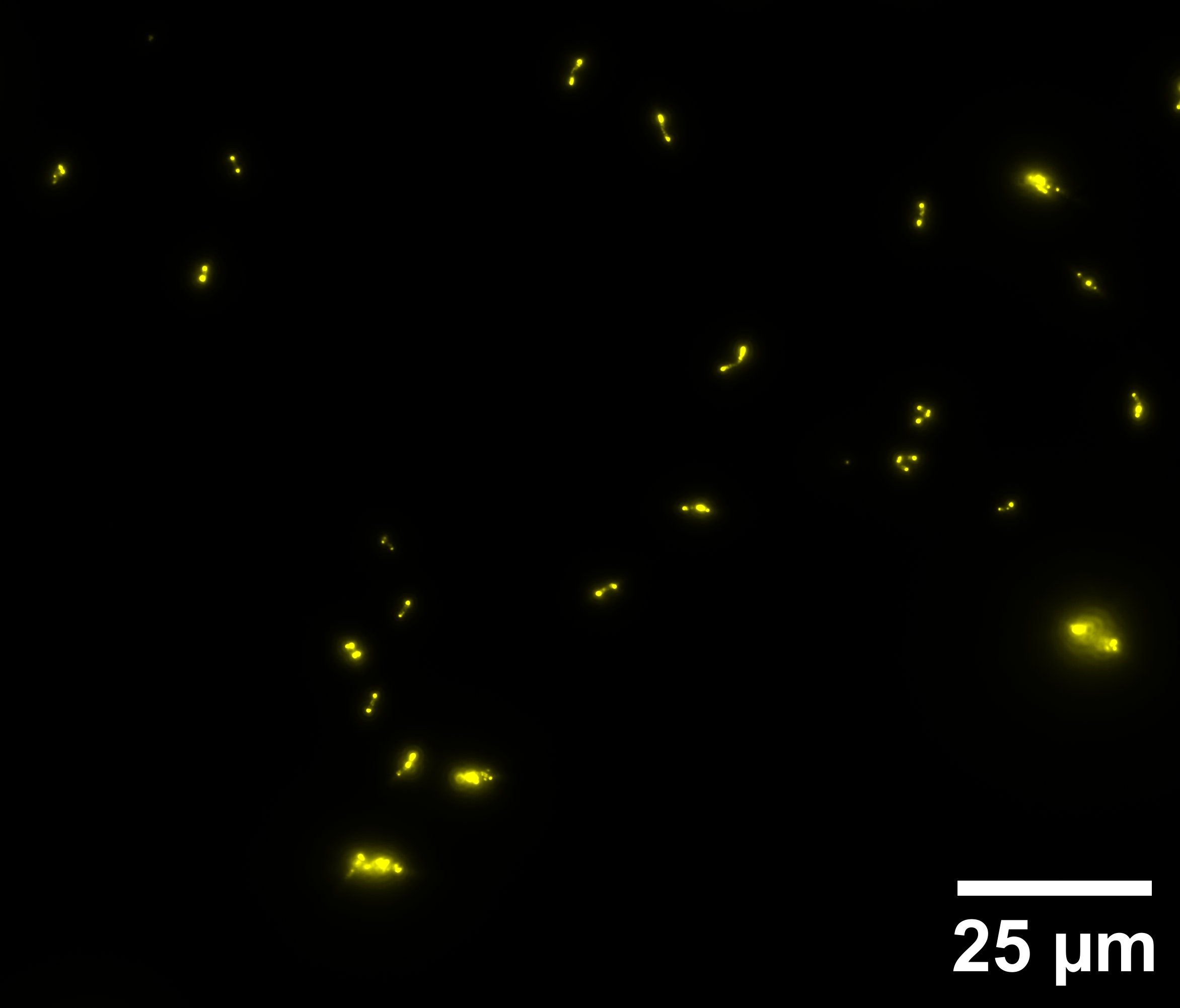

Supplement: Supplementary file 20 — Source data Fig. 4 [file 44318_2026_715_MOESM20_ESM.zip › Figure 4/Figure 4B/Figure 4B top right.jpg]

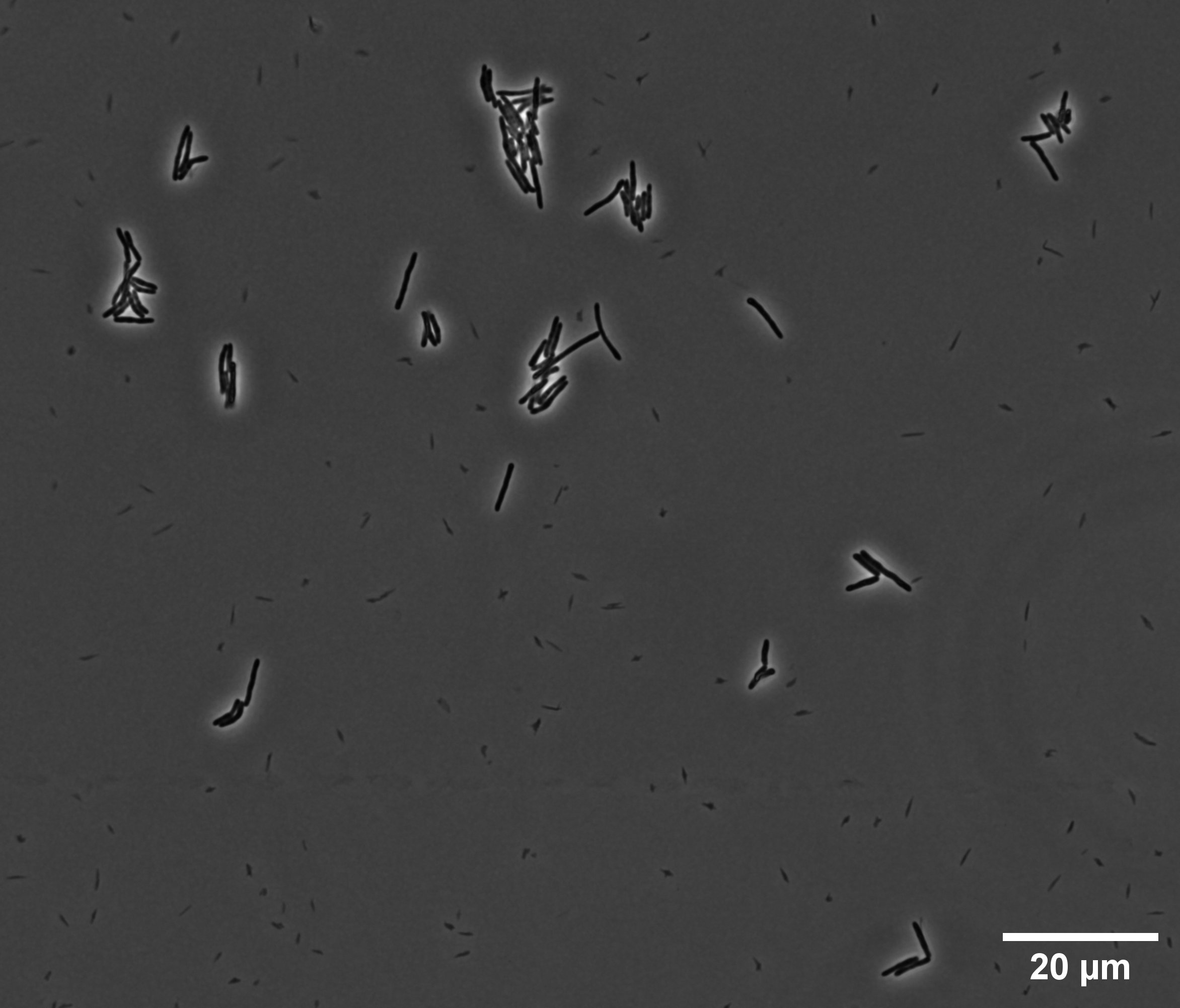

Supplement: Supplementary file 20 — Source data Fig. 4 [file 44318_2026_715_MOESM20_ESM.zip › Figure 4/Figure 4E/Figure 4E bottom left.jpg]

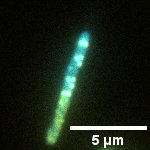

Supplement: Supplementary file 20 — Source data Fig. 4 [file 44318_2026_715_MOESM20_ESM.zip › Figure 4/Figure 4E/Figure 4E bottom right.jpg]

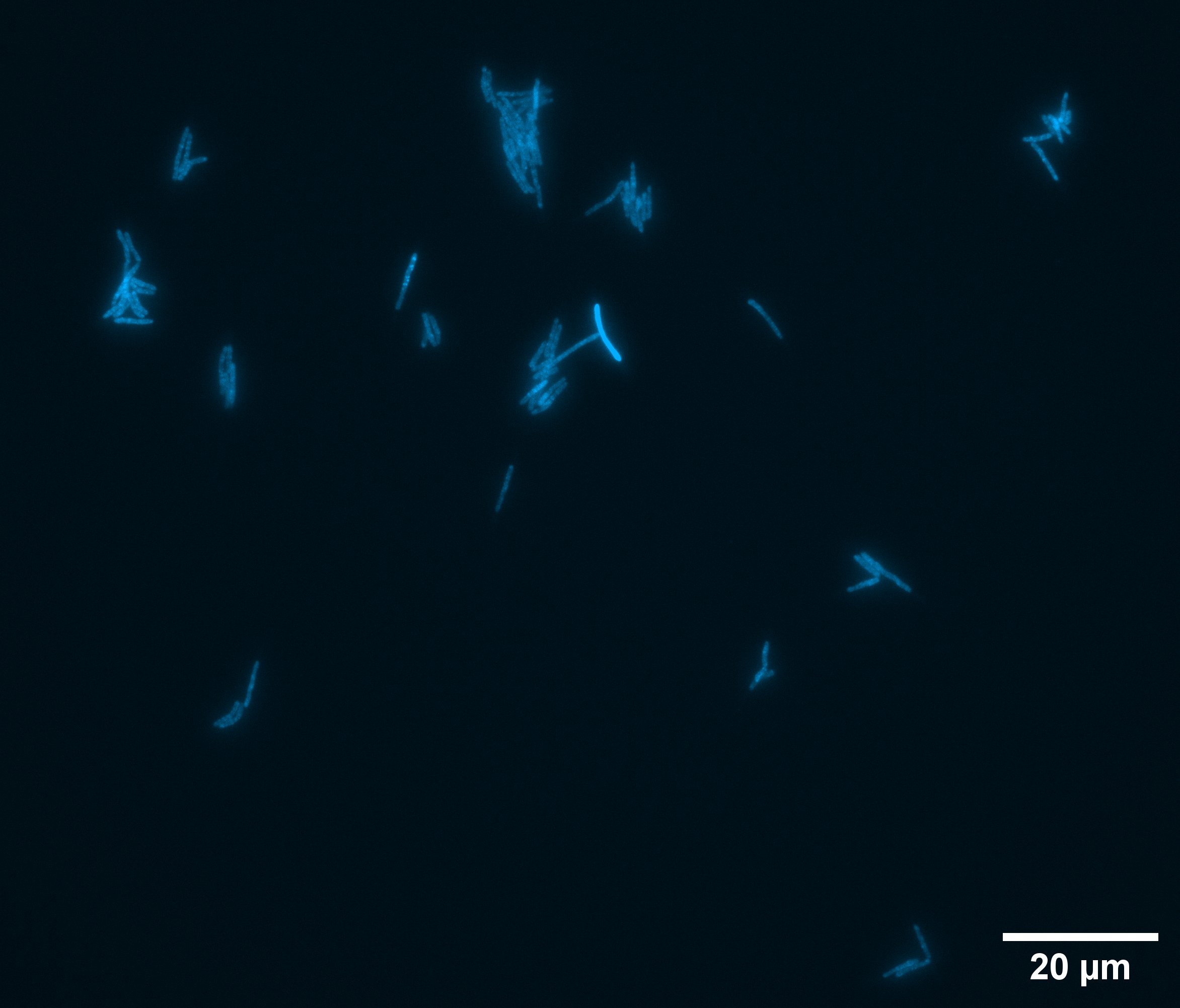

Supplement: Supplementary file 20 — Source data Fig. 4 [file 44318_2026_715_MOESM20_ESM.zip › Figure 4/Figure 4E/Figure 4E top left.jpg]

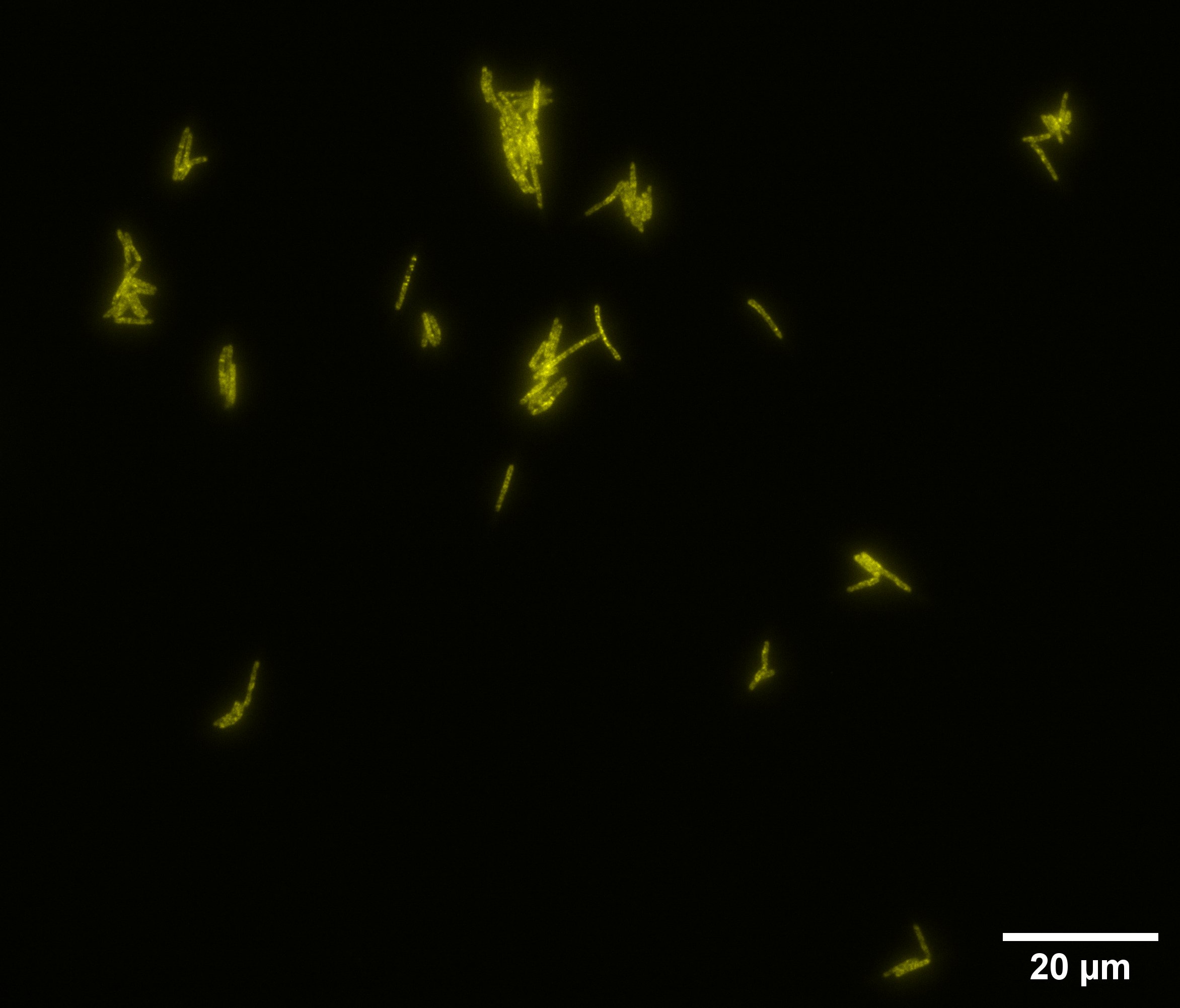

Supplement: Supplementary file 20 — Source data Fig. 4 [file 44318_2026_715_MOESM20_ESM.zip › Figure 4/Figure 4E/Figure 4E top right.jpg]

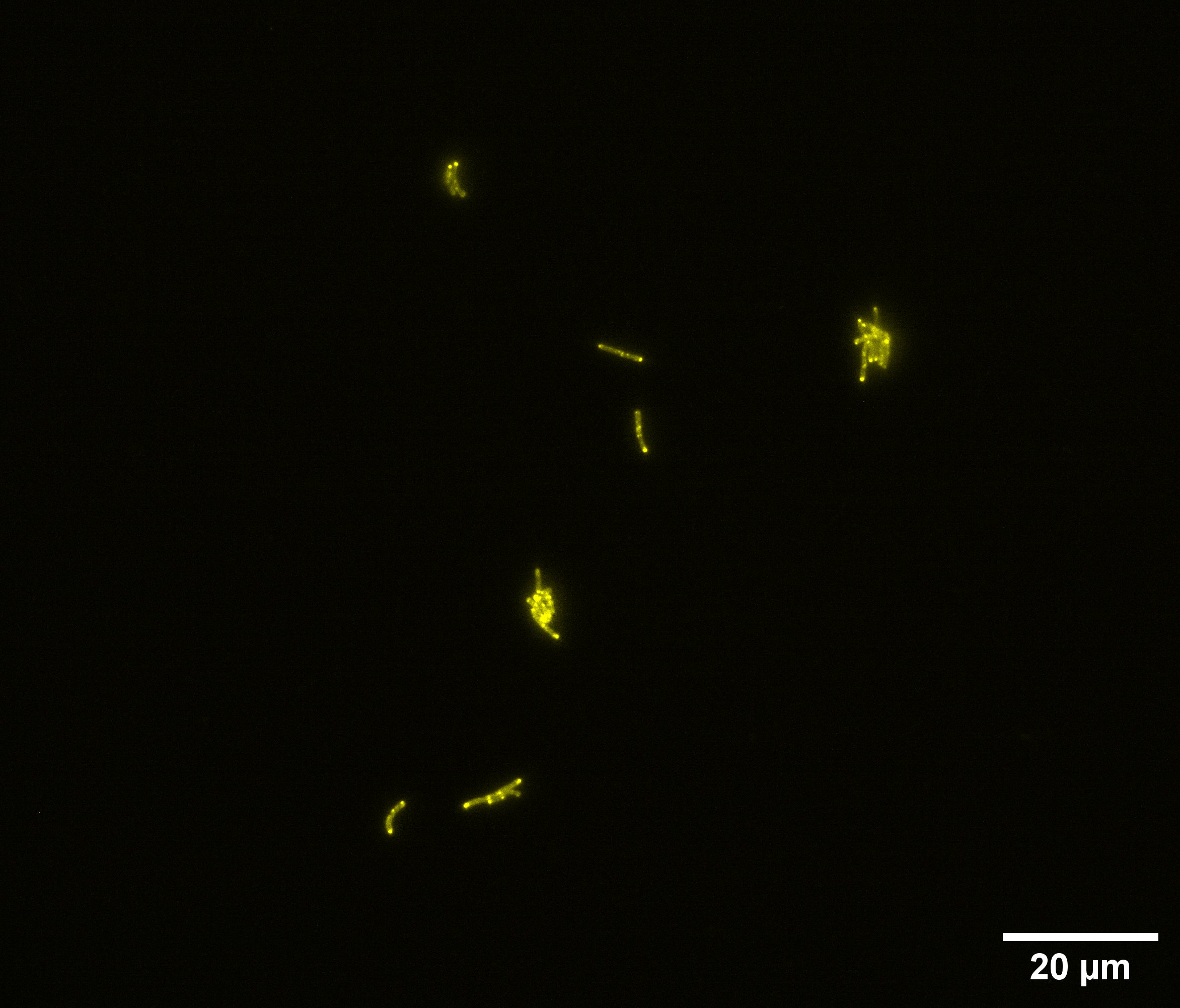

Supplement: Supplementary file 20 — Source data Fig. 4 [file 44318_2026_715_MOESM20_ESM.zip › Figure 4/Figure 4J/Figure 4J bottom left.jpg]

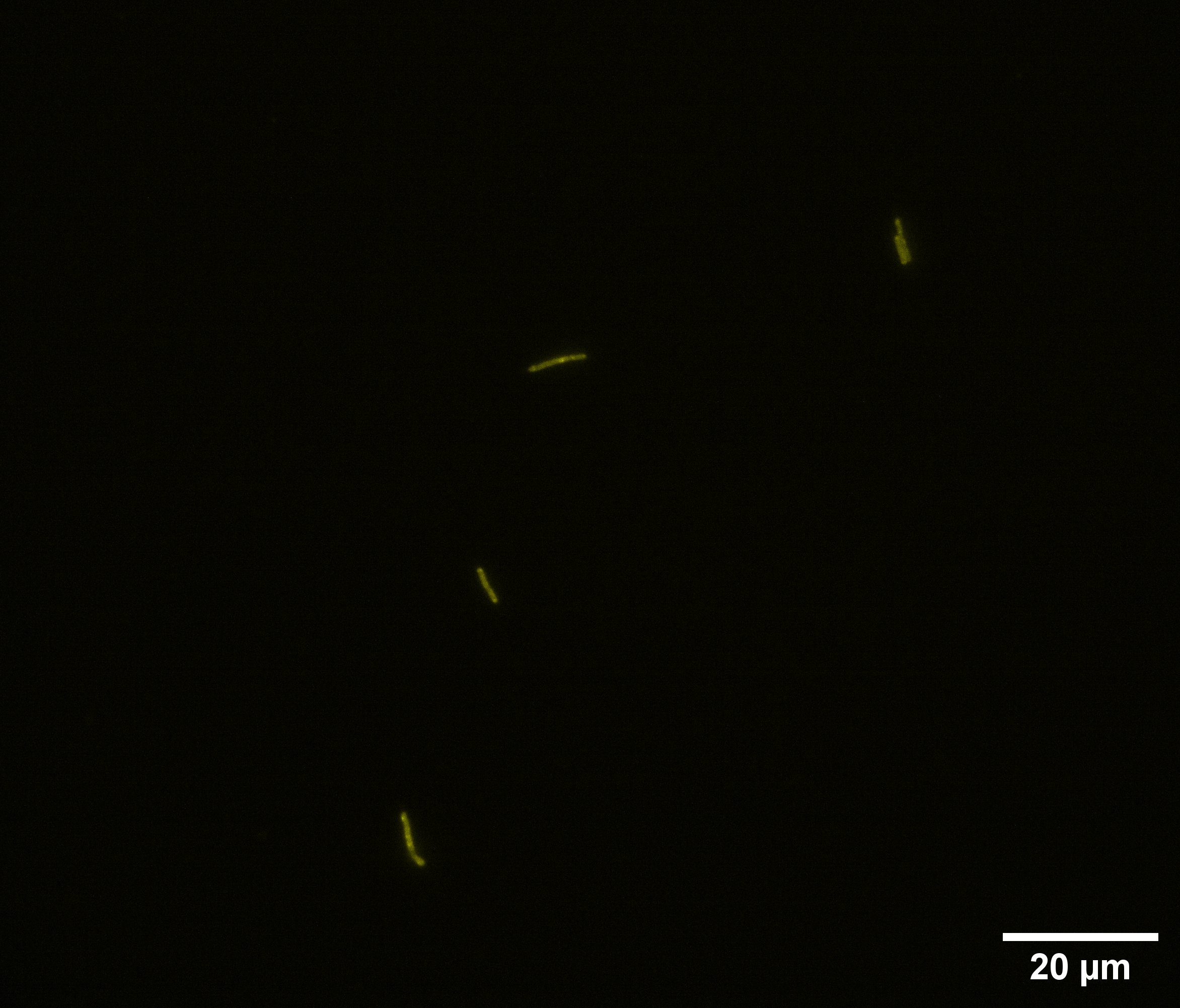

Supplement: Supplementary file 20 — Source data Fig. 4 [file 44318_2026_715_MOESM20_ESM.zip › Figure 4/Figure 4J/Figure 4J bottom right.jpg]

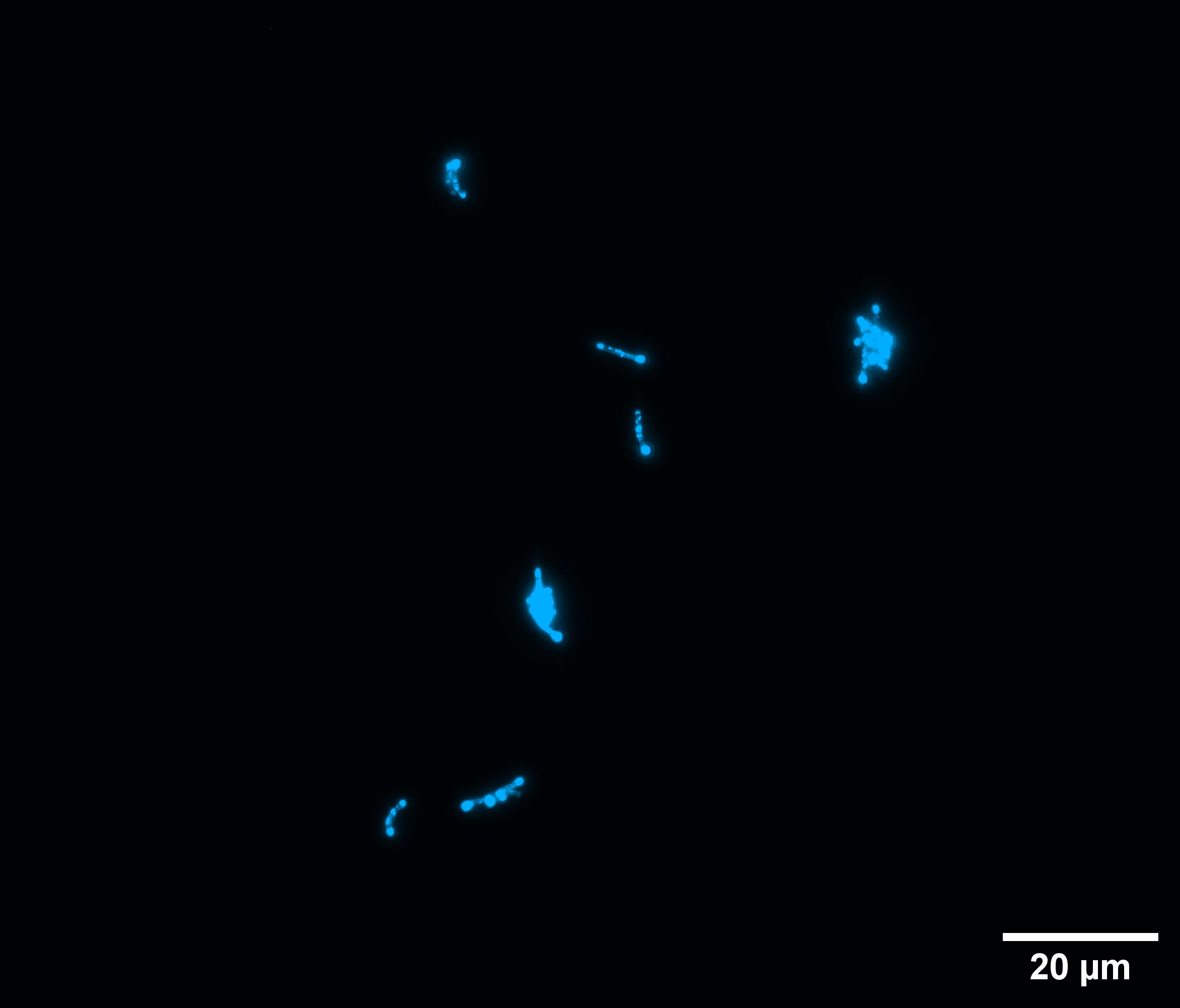

Supplement: Supplementary file 20 — Source data Fig. 4 [file 44318_2026_715_MOESM20_ESM.zip › Figure 4/Figure 4J/Figure 4J top left.jpg]

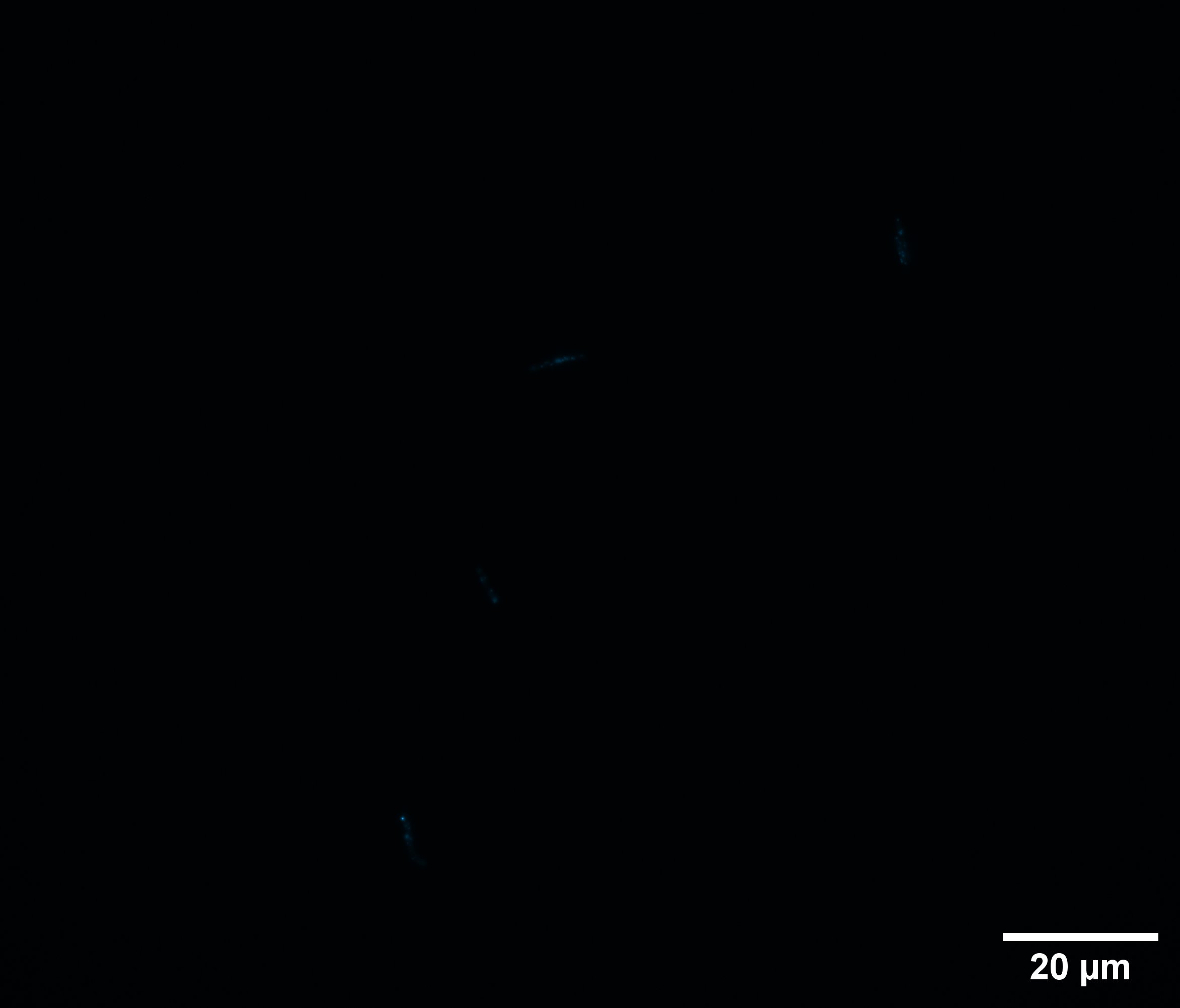

Supplement: Supplementary file 20 — Source data Fig. 4 [file 44318_2026_715_MOESM20_ESM.zip › Figure 4/Figure 4J/Figure 4J top right.jpg]

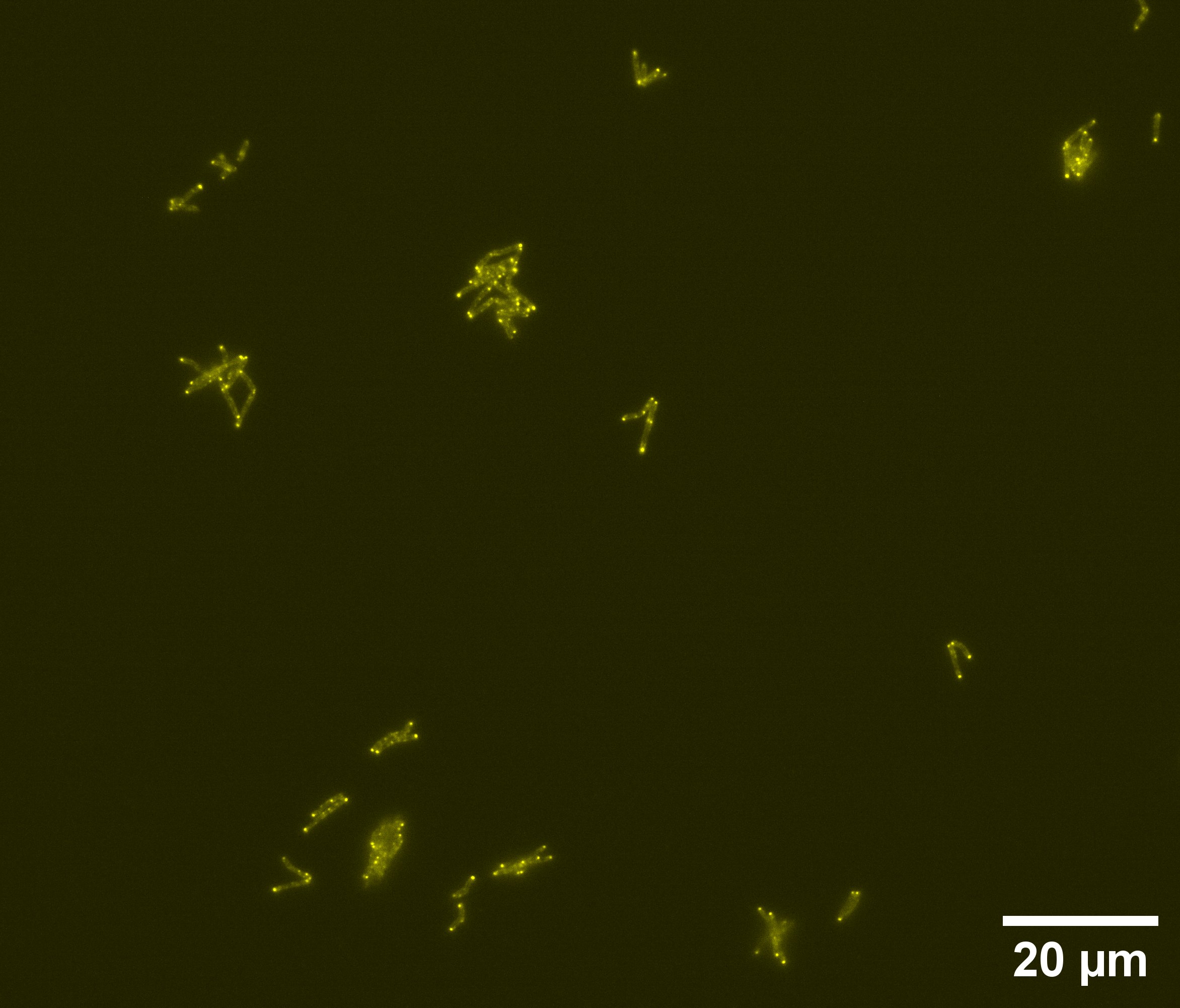

Supplement: Supplementary file 22 — Source data Fig. 6 [file 44318_2026_715_MOESM22_ESM.zip › Figure 6/Figure 6B/Figure 6B bottom left.jpg]

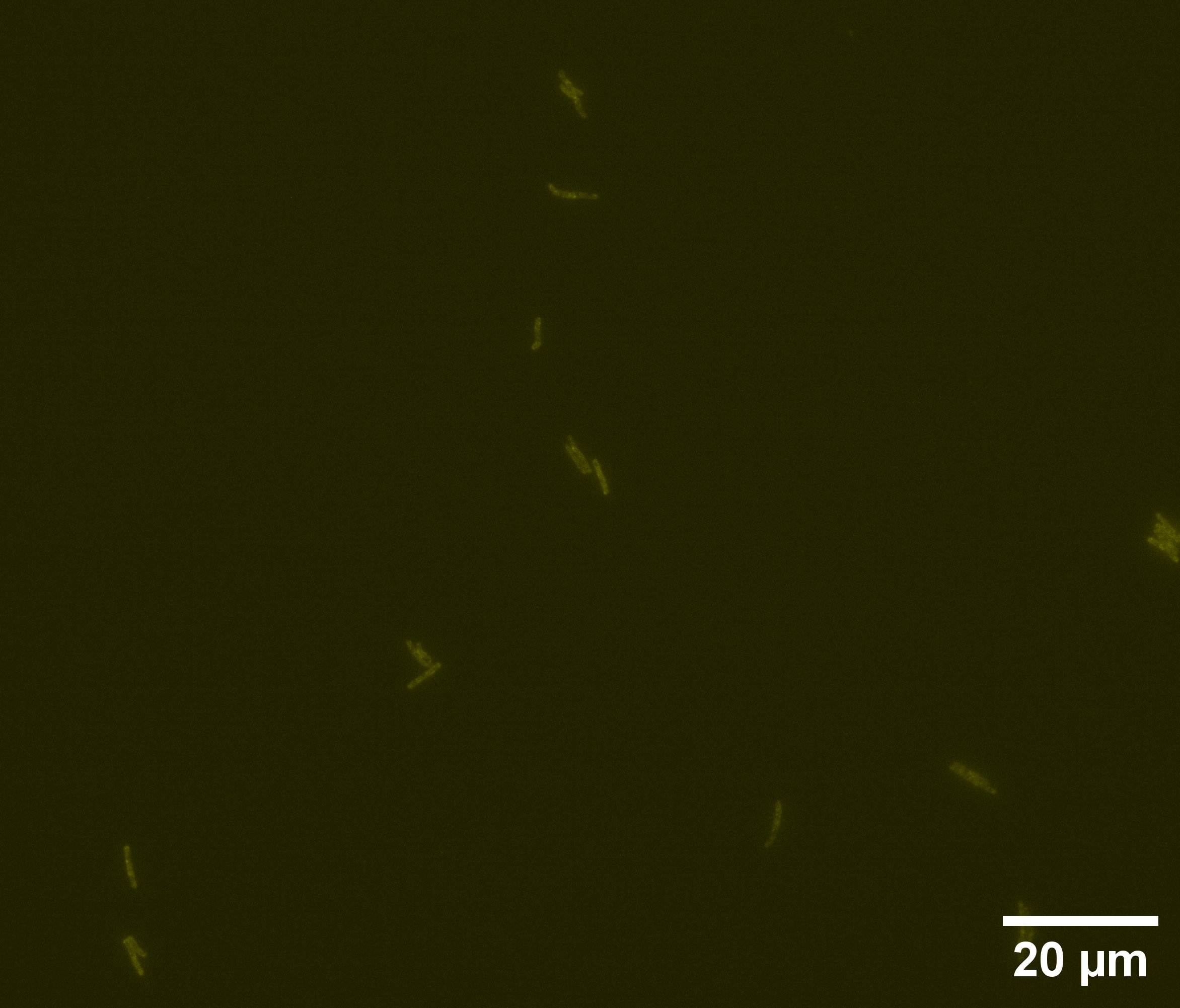

Supplement: Supplementary file 22 — Source data Fig. 6 [file 44318_2026_715_MOESM22_ESM.zip › Figure 6/Figure 6B/Figure 6B bottom right.jpg]

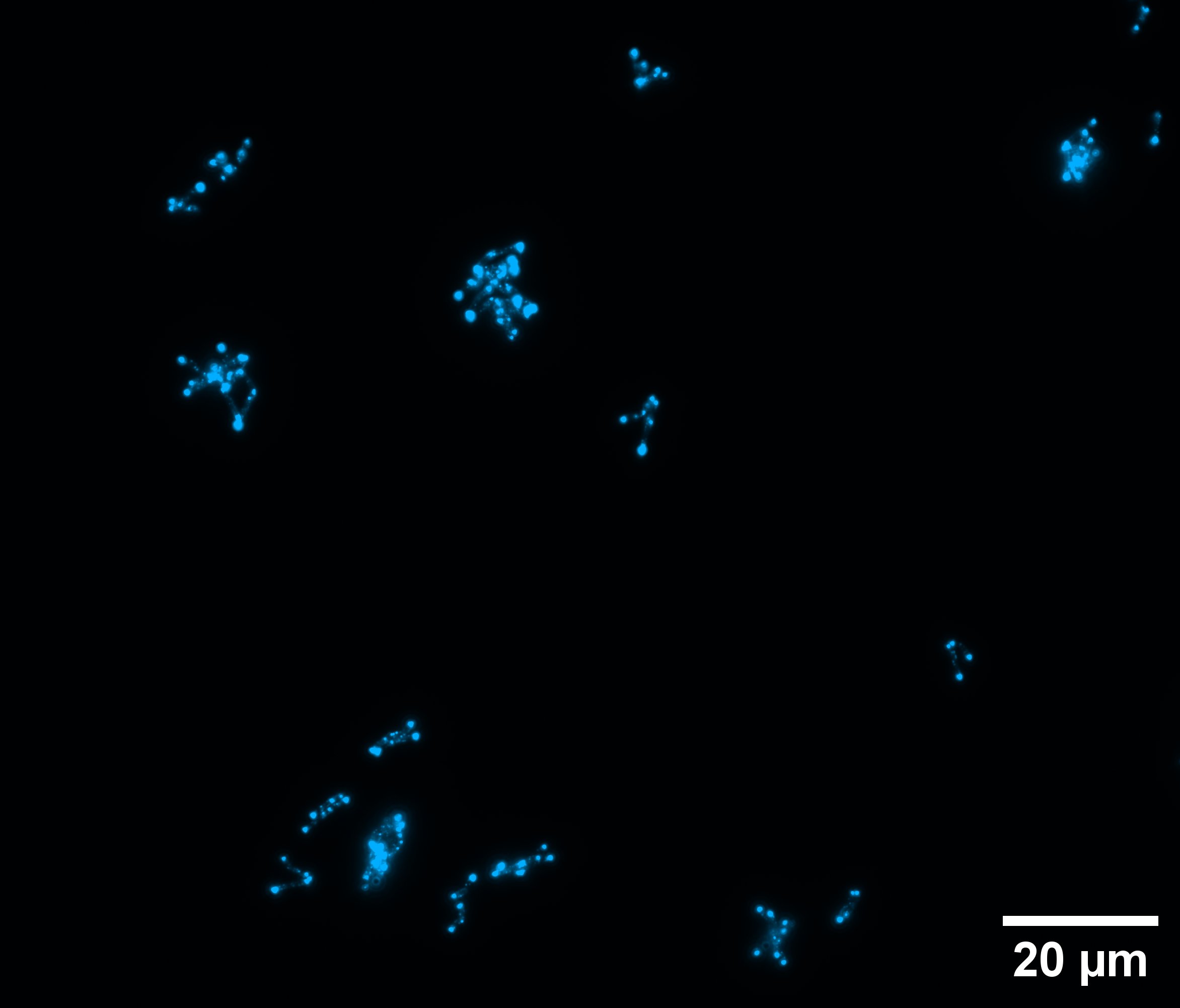

Supplement: Supplementary file 22 — Source data Fig. 6 [file 44318_2026_715_MOESM22_ESM.zip › Figure 6/Figure 6B/Figure 6B top left.jpg]

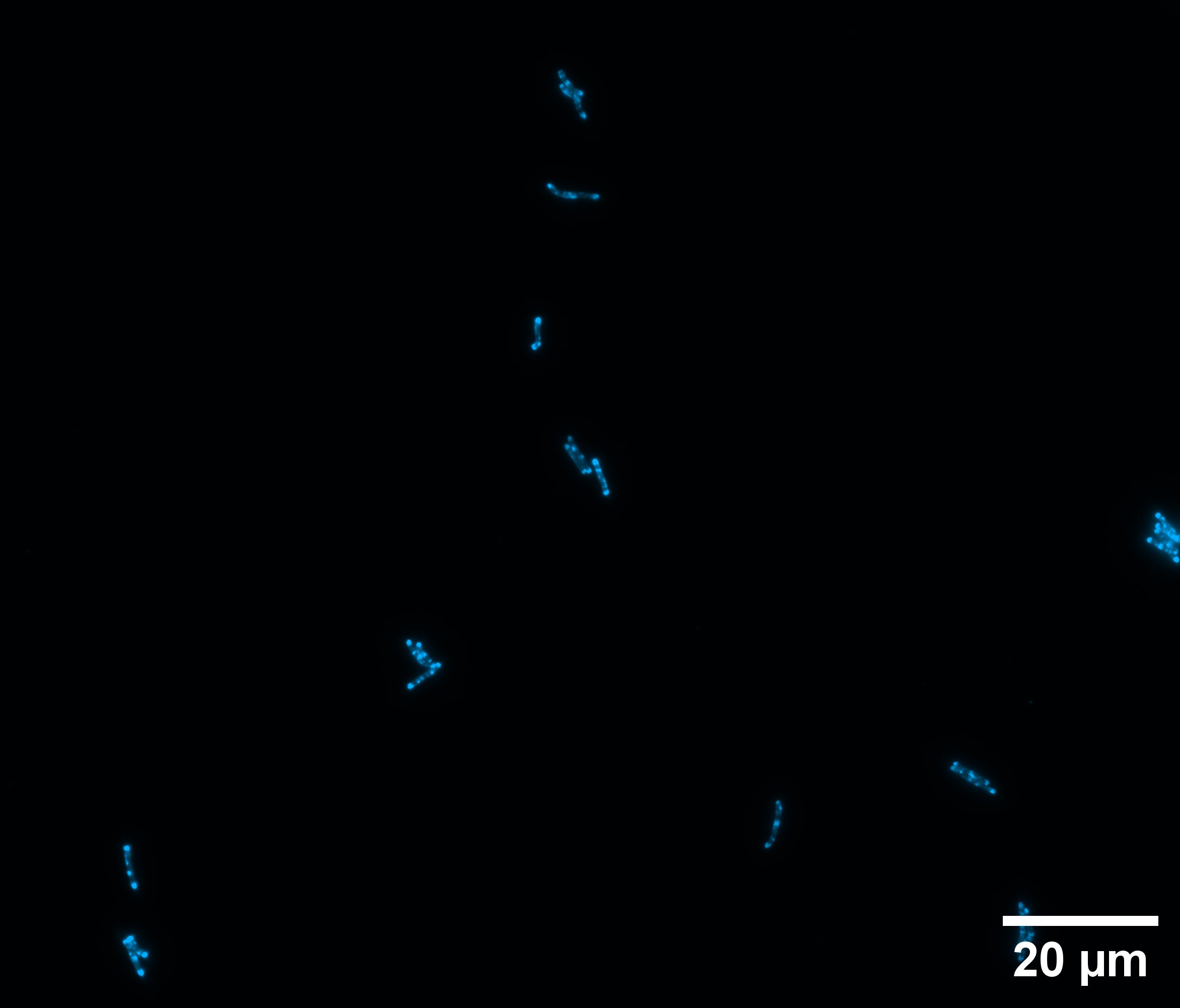

Supplement: Supplementary file 22 — Source data Fig. 6 [file 44318_2026_715_MOESM22_ESM.zip › Figure 6/Figure 6B/Figure 6B top right.jpg]

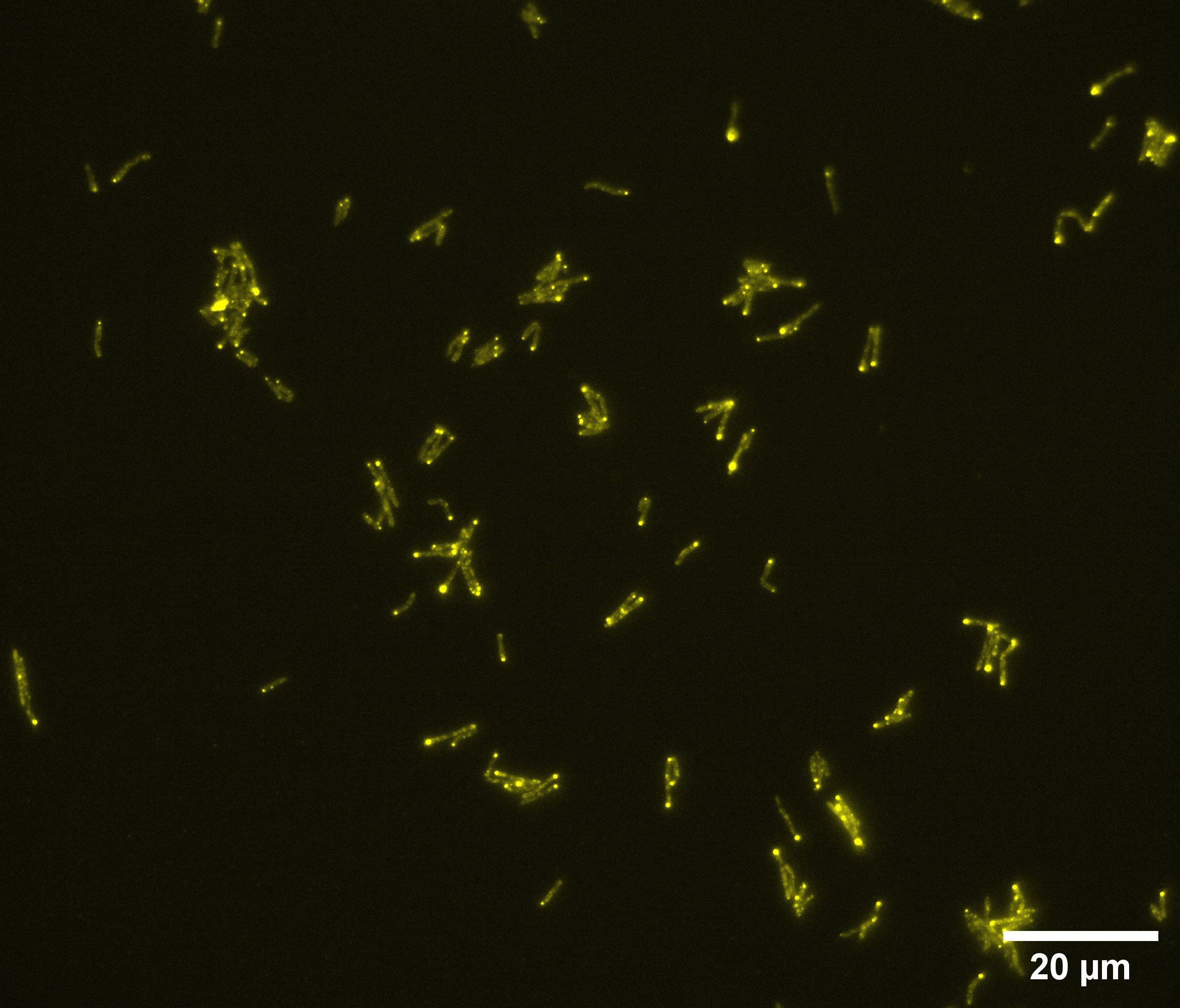

Supplement: Supplementary file 22 — Source data Fig. 6 [file 44318_2026_715_MOESM22_ESM.zip › Figure 6/Figure 6G/Figure 6G bottom left.jpg]

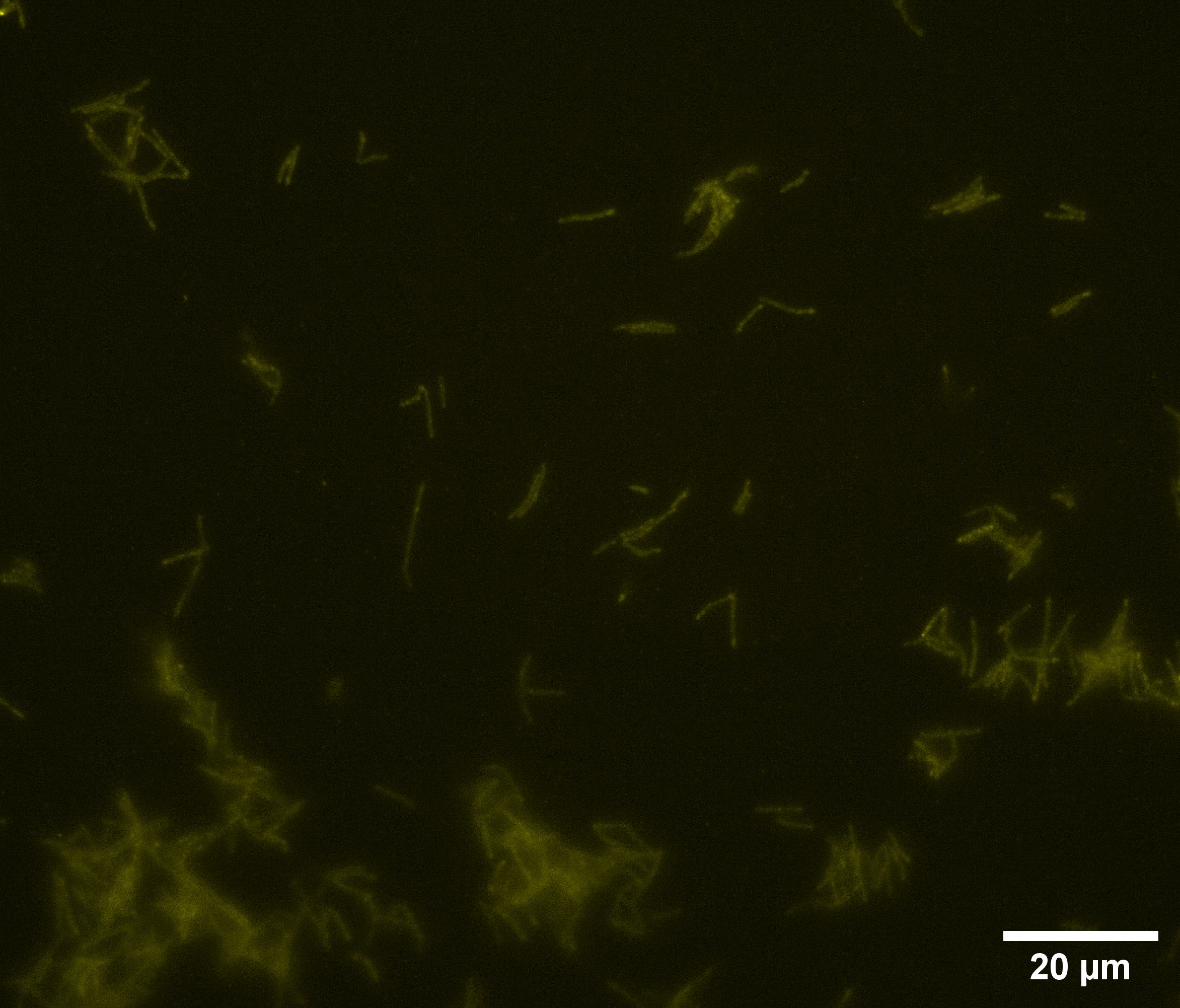

Supplement: Supplementary file 22 — Source data Fig. 6 [file 44318_2026_715_MOESM22_ESM.zip › Figure 6/Figure 6G/Figure 6G bottom middle.jpg]

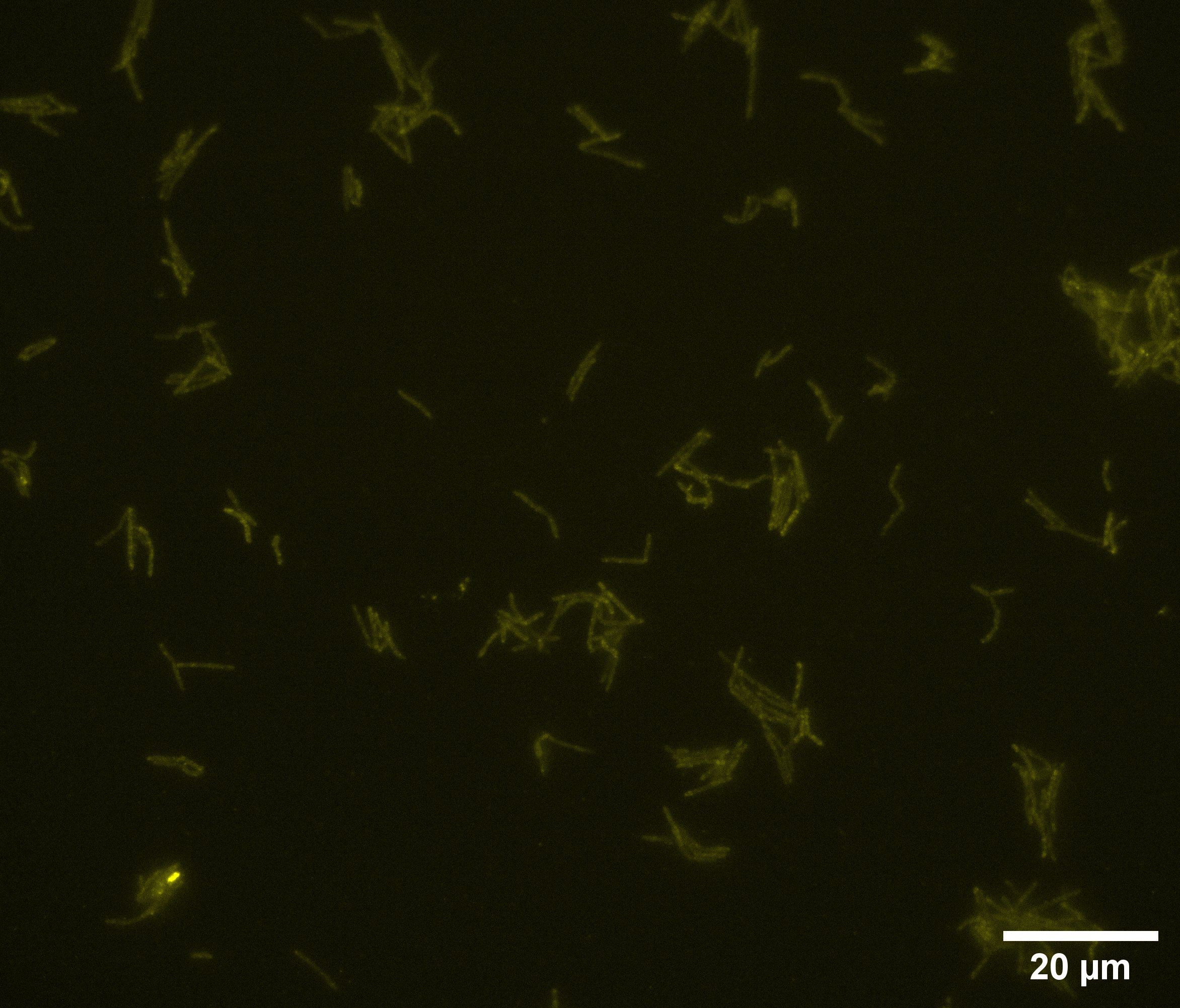

Supplement: Supplementary file 22 — Source data Fig. 6 [file 44318_2026_715_MOESM22_ESM.zip › Figure 6/Figure 6G/Figure 6G bottom right.jpg]

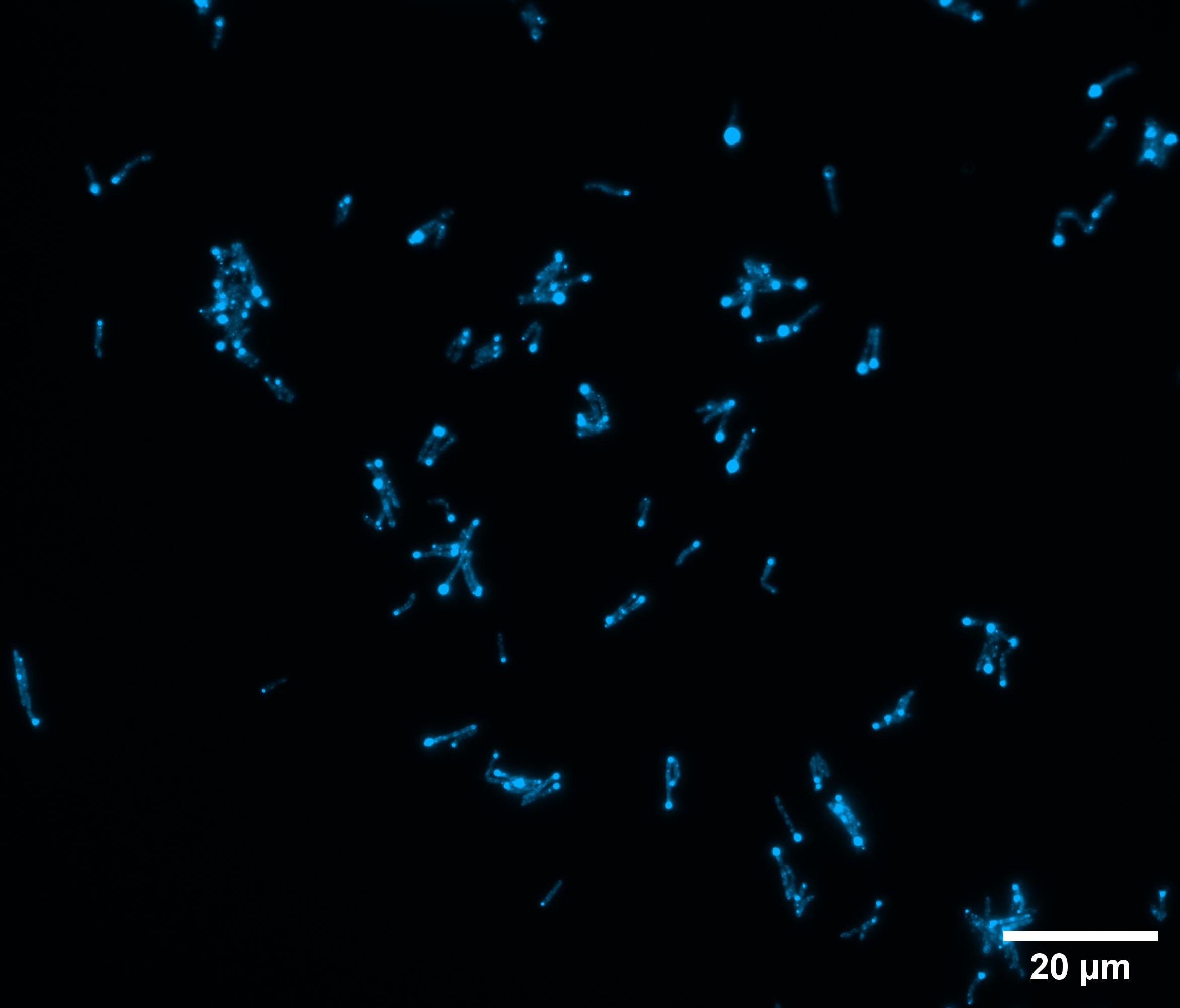

Supplement: Supplementary file 22 — Source data Fig. 6 [file 44318_2026_715_MOESM22_ESM.zip › Figure 6/Figure 6G/Figure 6G top left.jpg]

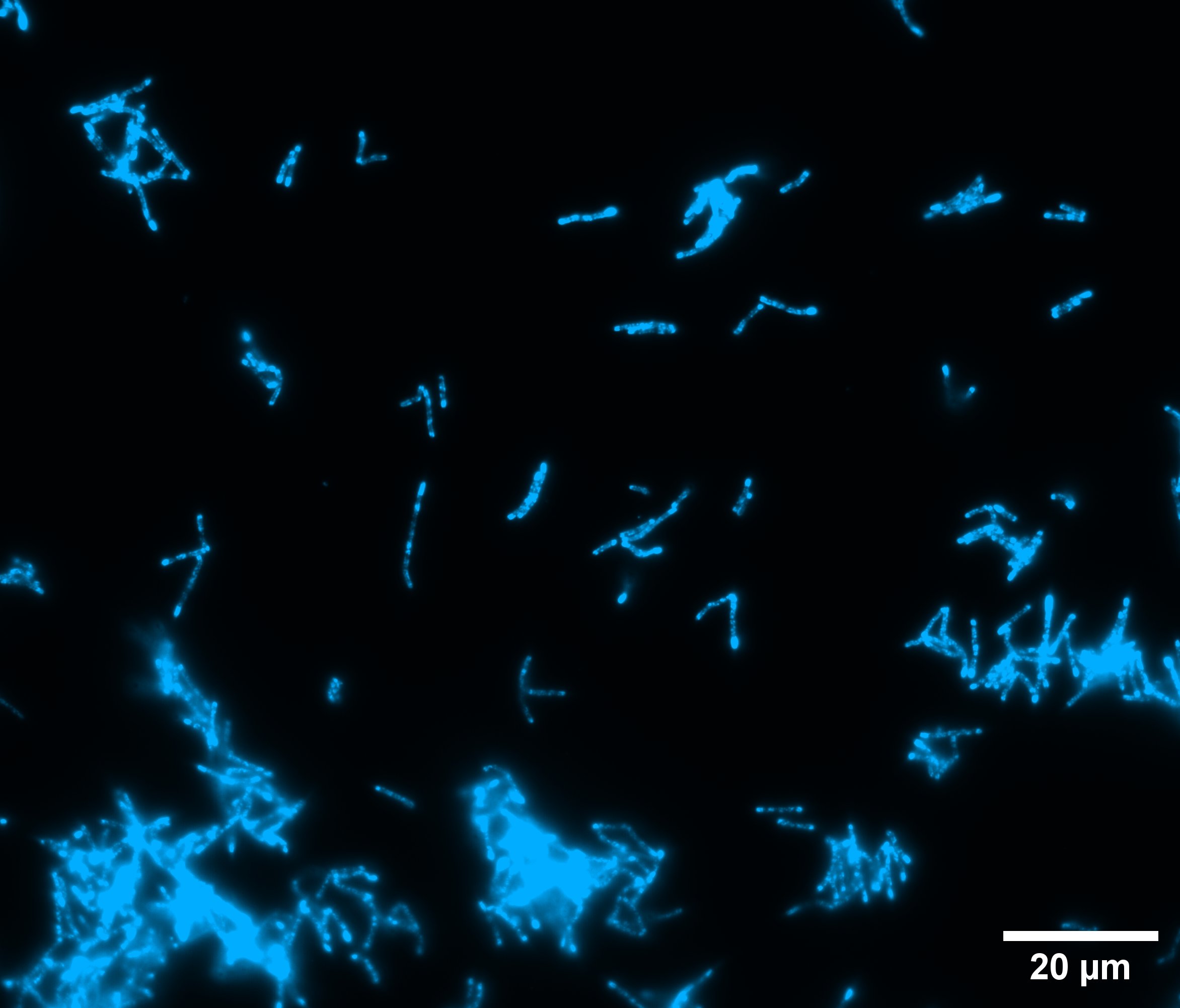

Supplement: Supplementary file 22 — Source data Fig. 6 [file 44318_2026_715_MOESM22_ESM.zip › Figure 6/Figure 6G/Figure 6G top middle.jpg]

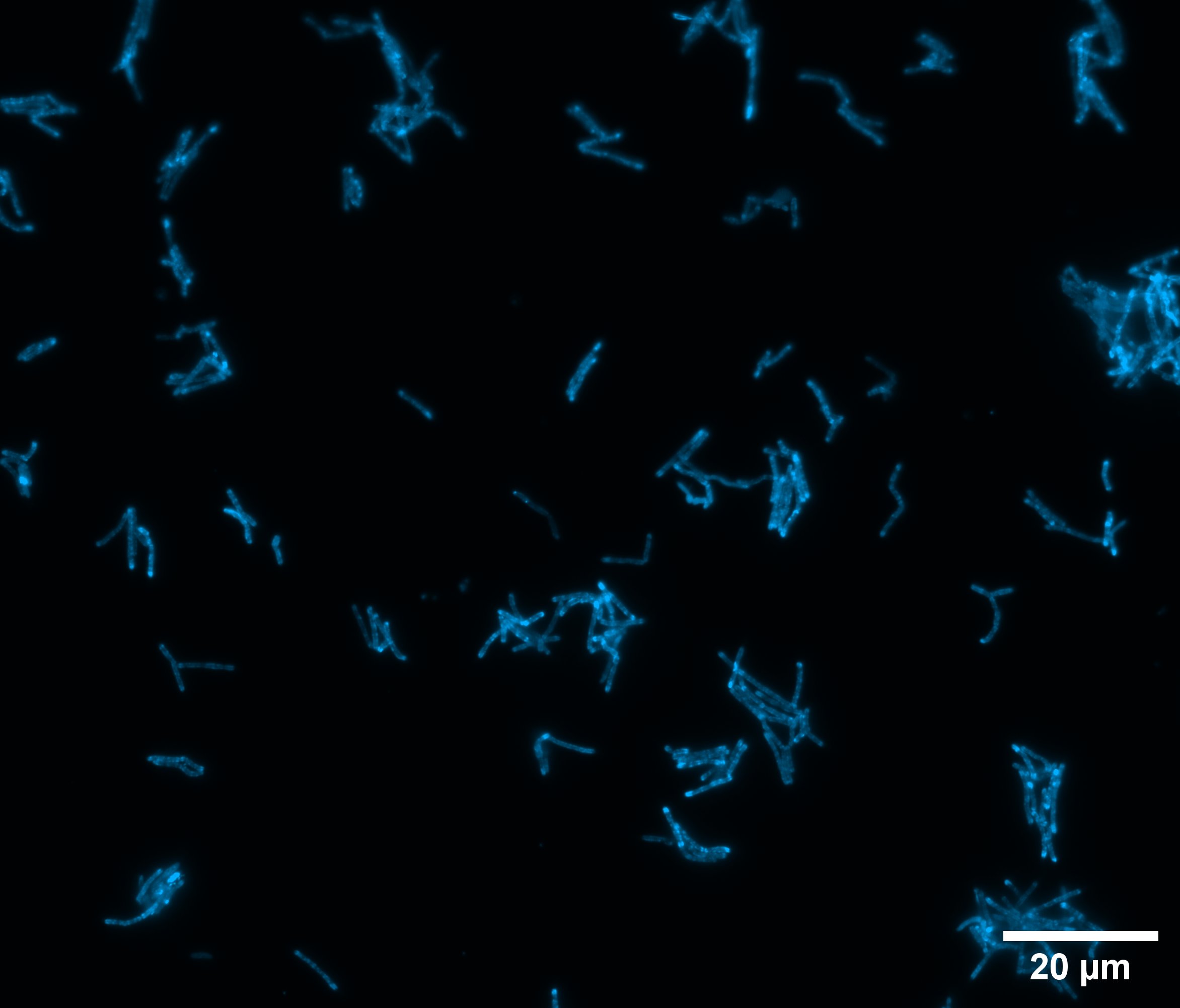

Supplement: Supplementary file 22 — Source data Fig. 6 [file 44318_2026_715_MOESM22_ESM.zip › Figure 6/Figure 6G/Figure 6G top right.jpg]

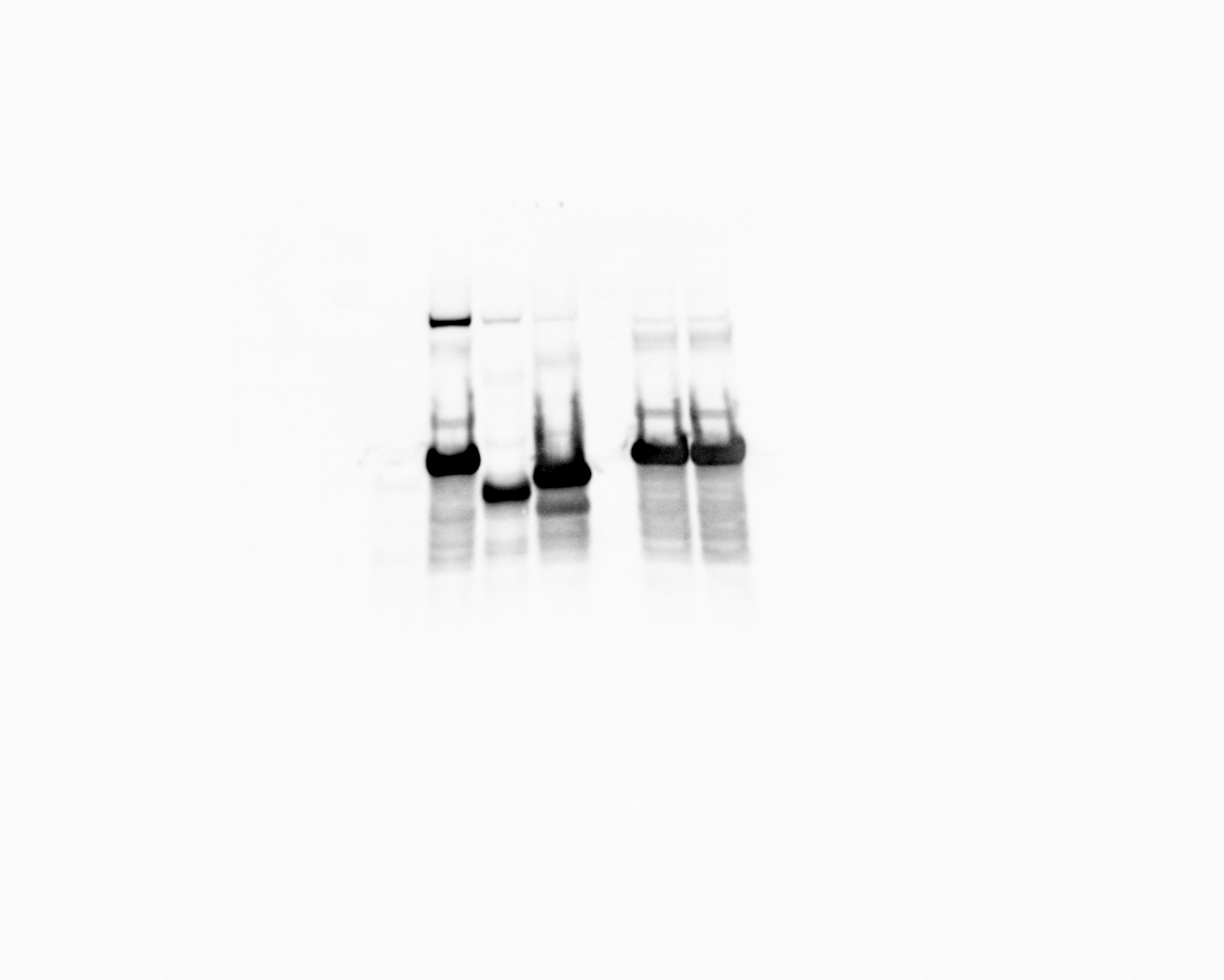

Supplement: Supplementary file 22 — Source data Fig. 6 [file 44318_2026_715_MOESM22_ESM.zip › Figure 6/Figure 6J/Figure 6J.jpg]

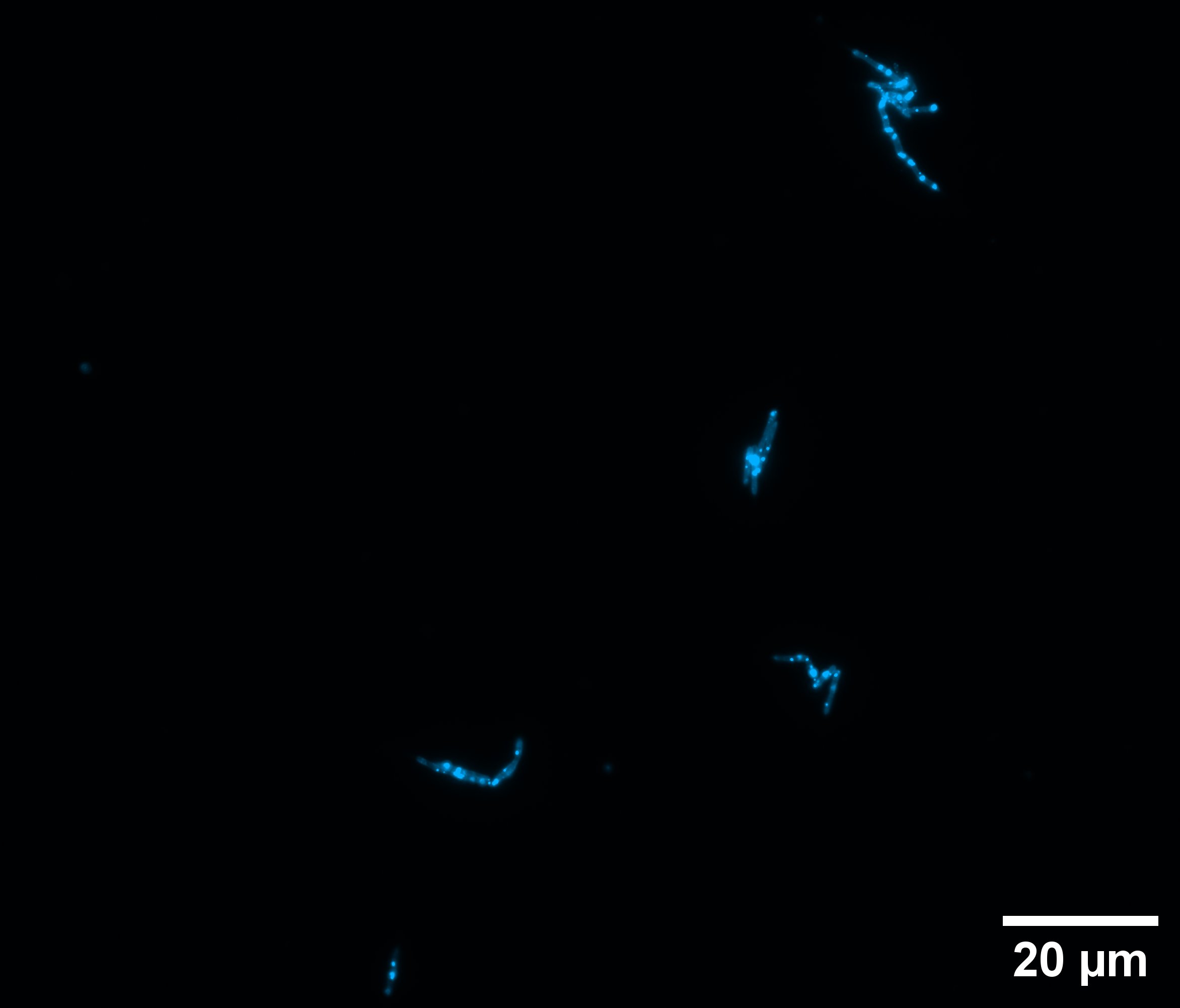

Supplement: Supplementary file 23 — Source data Fig. 7 [file 44318_2026_715_MOESM23_ESM.zip › Figure 7/Figure 7C/Figure 7C - bottom middle.jpg]

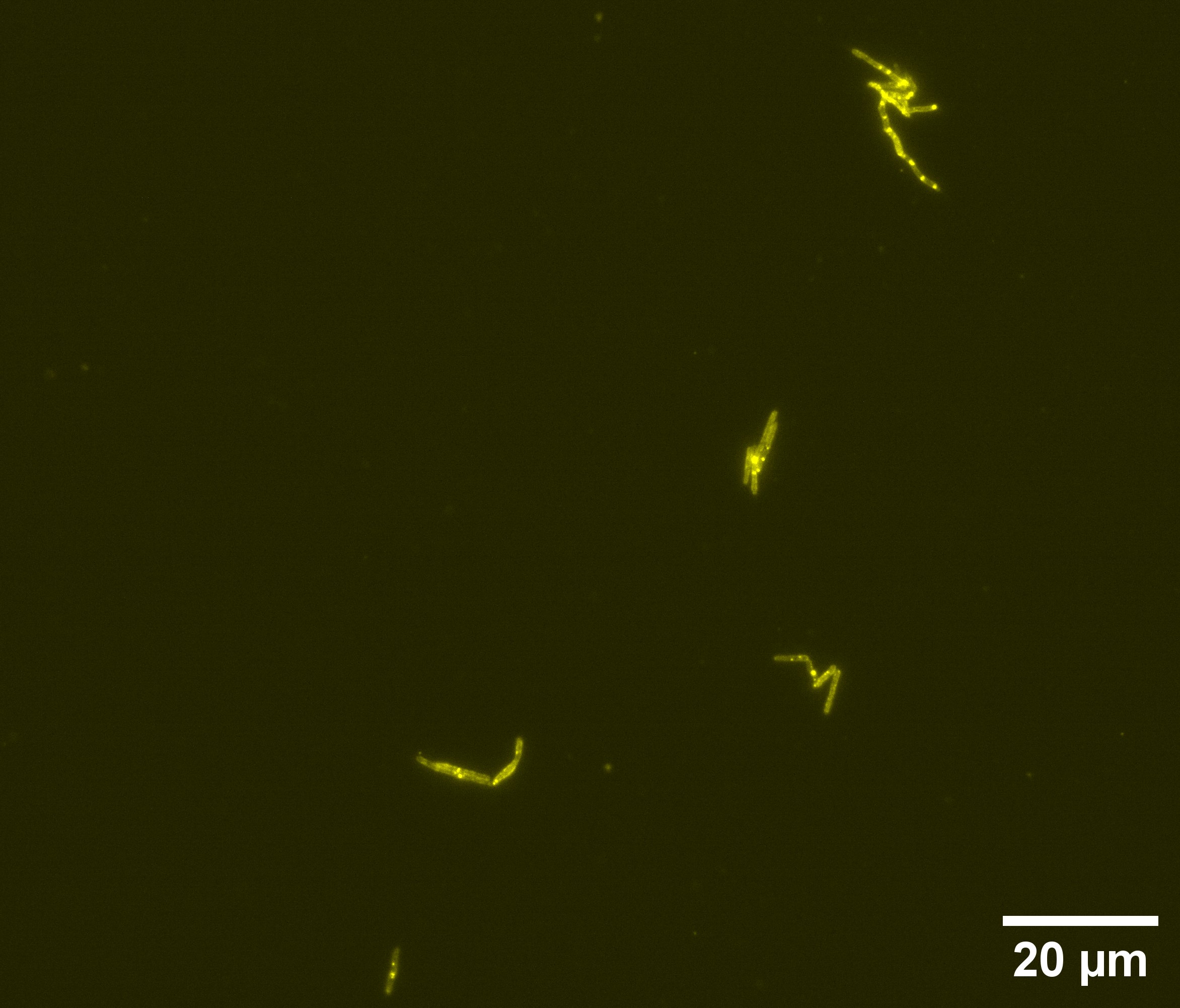

Supplement: Supplementary file 23 — Source data Fig. 7 [file 44318_2026_715_MOESM23_ESM.zip › Figure 7/Figure 7C/Figure 7C - bottom right.jpg]

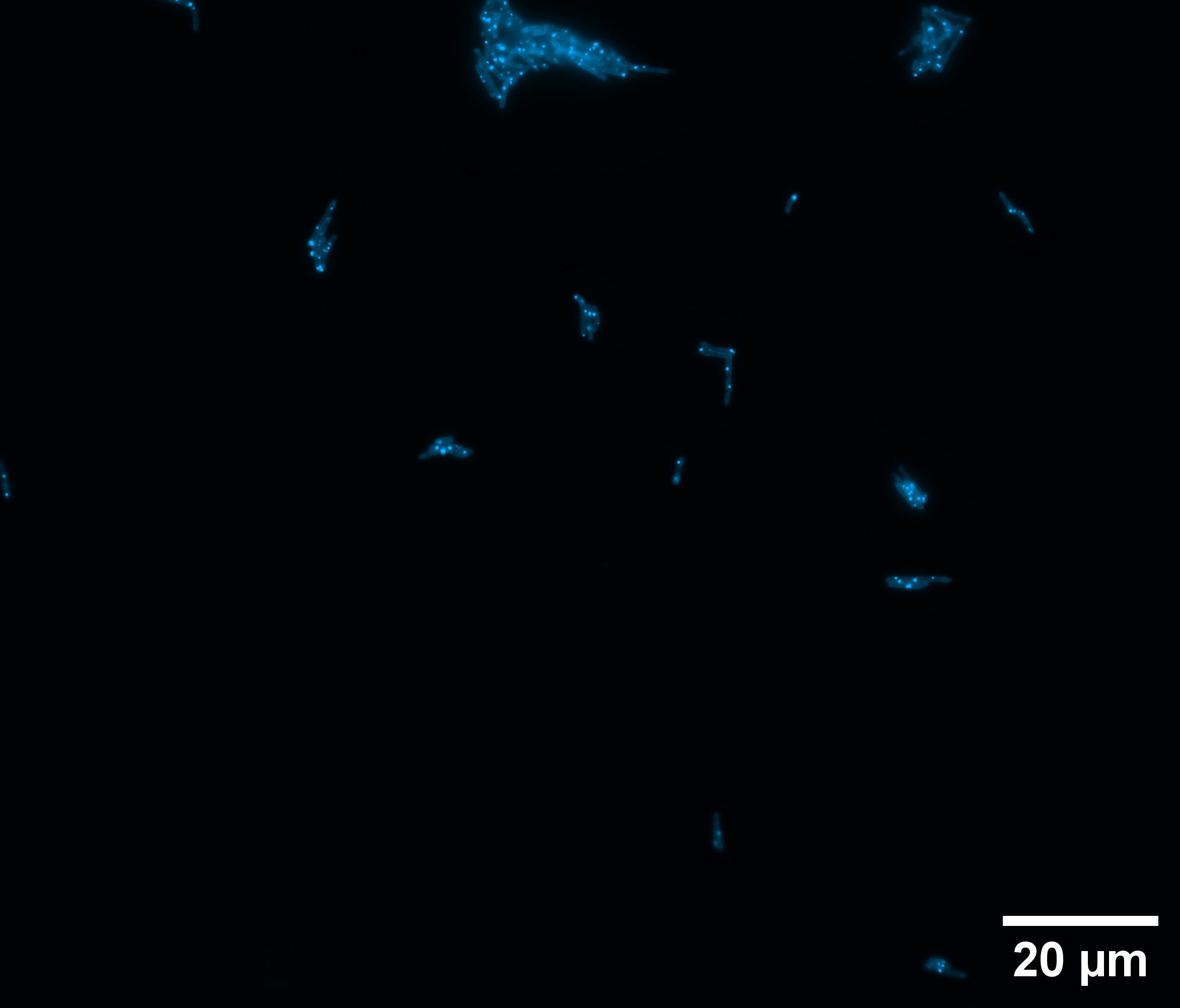

Supplement: Supplementary file 23 — Source data Fig. 7 [file 44318_2026_715_MOESM23_ESM.zip › Figure 7/Figure 7C/Figure 7C - middle middle.jpg]

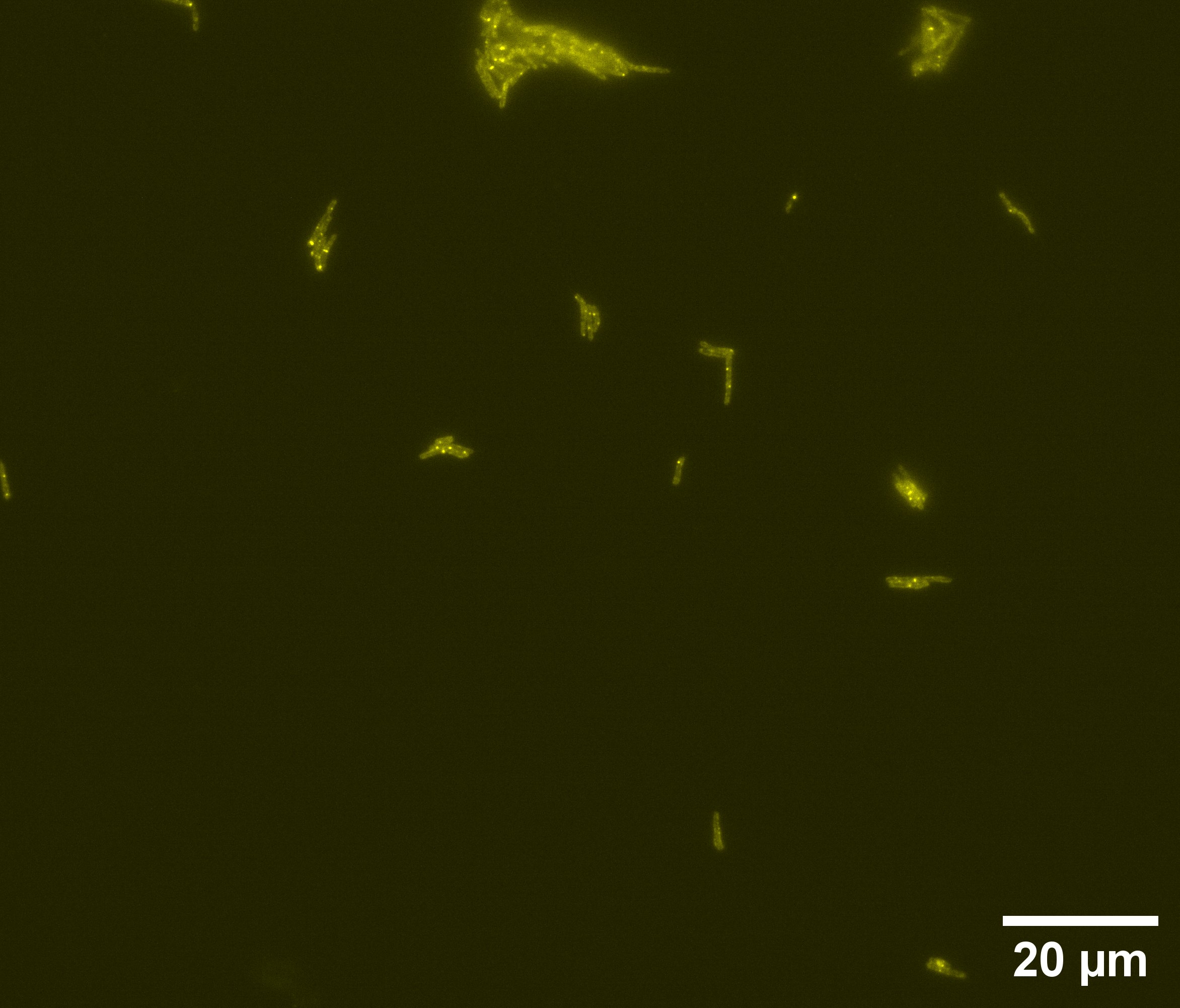

Supplement: Supplementary file 23 — Source data Fig. 7 [file 44318_2026_715_MOESM23_ESM.zip › Figure 7/Figure 7C/Figure 7C - middle right.jpg]

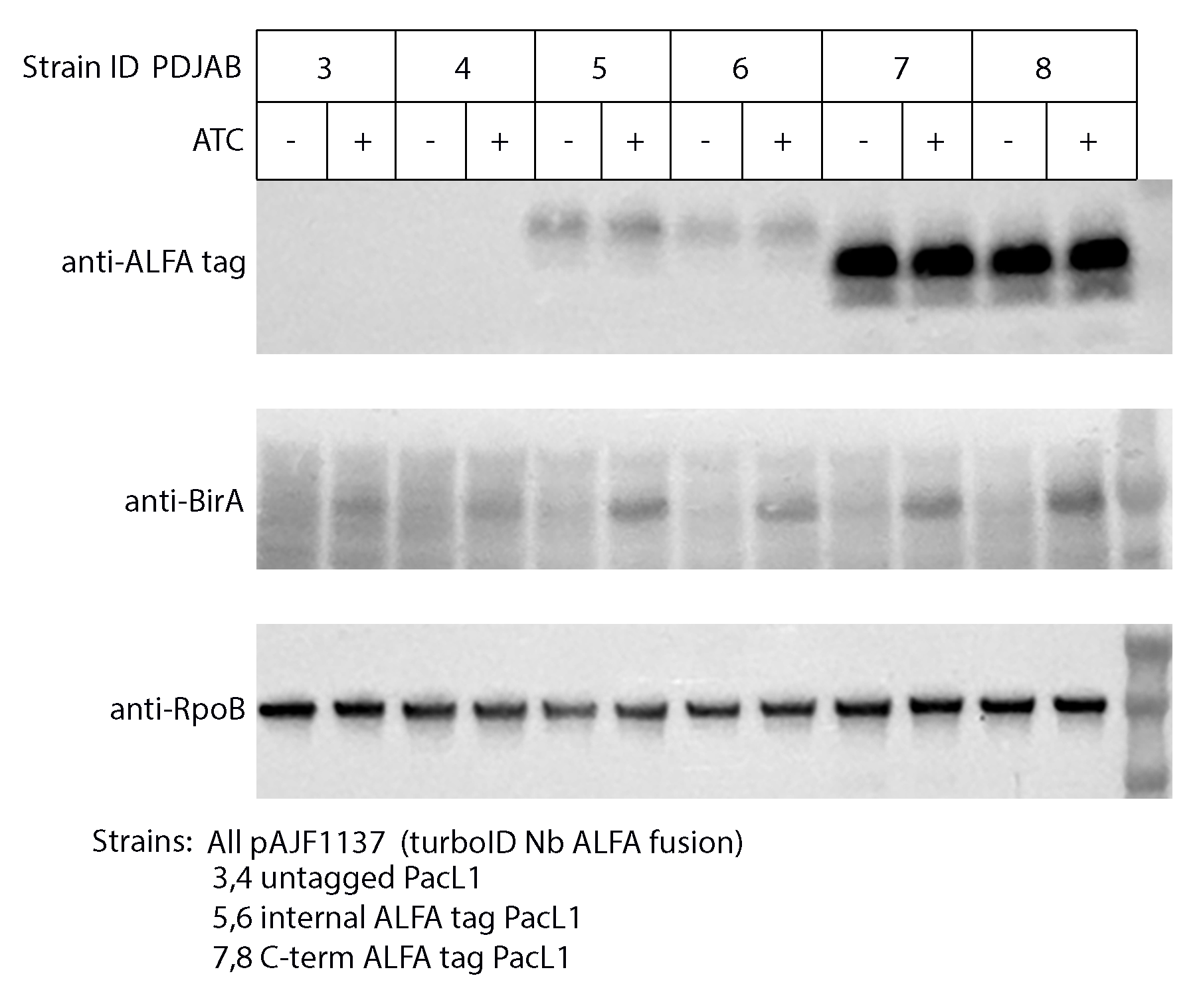

Supplement: Supplementary file 26 — Figure EV5 Source Data [file 44318_2026_715_MOESM26_ESM.zip › Figure EV5/Figure EV5B/Figure EV5B.tif]
