# Supplementary material for: Membrane-associated effluxosomes coordinate multi-metal resistance in Mycobacterium tuberculosis
Source: EMBO J. 2026 Feb 13;45(7):2306–37. doi: 10.1038/s44318-026-00715-1 (PMC13043812; doi:10.1038/s44318-026-00715-1)
Supplement: Supplementary file 11 — Movie EV3 [file 44318_2026_715_MOESM11_ESM.zip › Movie EV3/Movie EV3 legend.docx]

**Movie EV3. Super resolution localization of CtpG within the mycobacterial membrane.** 3D reconstruction of PacL2-mEos fusion protein localizations assessed by photoactivated localization microscopy (PALM) in live *M. smegmatis*. Green and magenta indicate unclustered and clustered proteins, respectively. Bacteria were cultured in the presence of 10 µM CdSO₄.
