# Supplementary material for: Membrane-associated effluxosomes coordinate multi-metal resistance in Mycobacterium tuberculosis
Source: EMBO J. 2026 Feb 13;45(7):2306–37. doi: 10.1038/s44318-026-00715-1 (PMC13043812; doi:10.1038/s44318-026-00715-1)
Supplement: Supplementary file 12 — Movie EV4 [file 44318_2026_715_MOESM12_ESM.zip › Movie EV4/Movie EV4 legend.docx]

**Movie EV4. Cluster segmentation of PacL2 proteins within the mycobacterial membrane.** 3D reconstruction of cluster segmentation using the PoCA software, based on single-molecule localizations of PacL2-mEos acquired by photoactivated localization microscopy (PALM) in live *M. smegmatis*. Bacteria were cultured in the presence of 10 µM CdSO₄.
