# Supplementary material for: Membrane-associated effluxosomes coordinate multi-metal resistance in Mycobacterium tuberculosis
Source: EMBO J. 2026 Feb 13;45(7):2306–37. doi: 10.1038/s44318-026-00715-1 (PMC13043812; doi:10.1038/s44318-026-00715-1)
Supplement: Supplementary file 14 — Movie EV6 [file 44318_2026_715_MOESM14_ESM.zip › Movie EV6/Movie EV6 legend.docx]

**Movie EV6. Representative super-resolution reconstruction of PacL2 trajectories acquired by sptPALM.** Left: fluorescent signal of PacL2-mEoS obtained via sptPALM in live *M. smegmatis*. Right: reconstructed trajectories with lengths greater than 6 time points. Bacteria were cultured in the presence of 10 µM CdSO₄.
