# Supplementary material for: Membrane-associated effluxosomes coordinate multi-metal resistance in Mycobacterium tuberculosis
Source: EMBO J. 2026 Feb 13;45(7):2306–37. doi: 10.1038/s44318-026-00715-1 (PMC13043812; doi:10.1038/s44318-026-00715-1)
Supplement: Supplementary file 16 — Movie EV8 [file 44318_2026_715_MOESM16_ESM.zip › Movie EV8/Movie EV8 legend.docx]

**Movie EV8. The K9A mutation in PacL2 disrupts the assembly of small mobile clusters within the mycobacterial membrane.** Epifluorescence microscopy time-lapse imaging (45-second duration, with one image captured per second) of live *M. smegmatis* expressing the PacL2^K9A^-mTurquoise protein fusion. Bacteria were cultured in the presence of 10 µM CdSO₄.
