# Supplementary material for: Membrane-associated effluxosomes coordinate multi-metal resistance in Mycobacterium tuberculosis
Source: EMBO J. 2026 Feb 13;45(7):2306–37. doi: 10.1038/s44318-026-00715-1 (PMC13043812; doi:10.1038/s44318-026-00715-1)
Supplement: Supplementary file 27 — Expanded View Figures [file 44318_2026_715_MOESM27_ESM.pdf]

Expanded View Figures

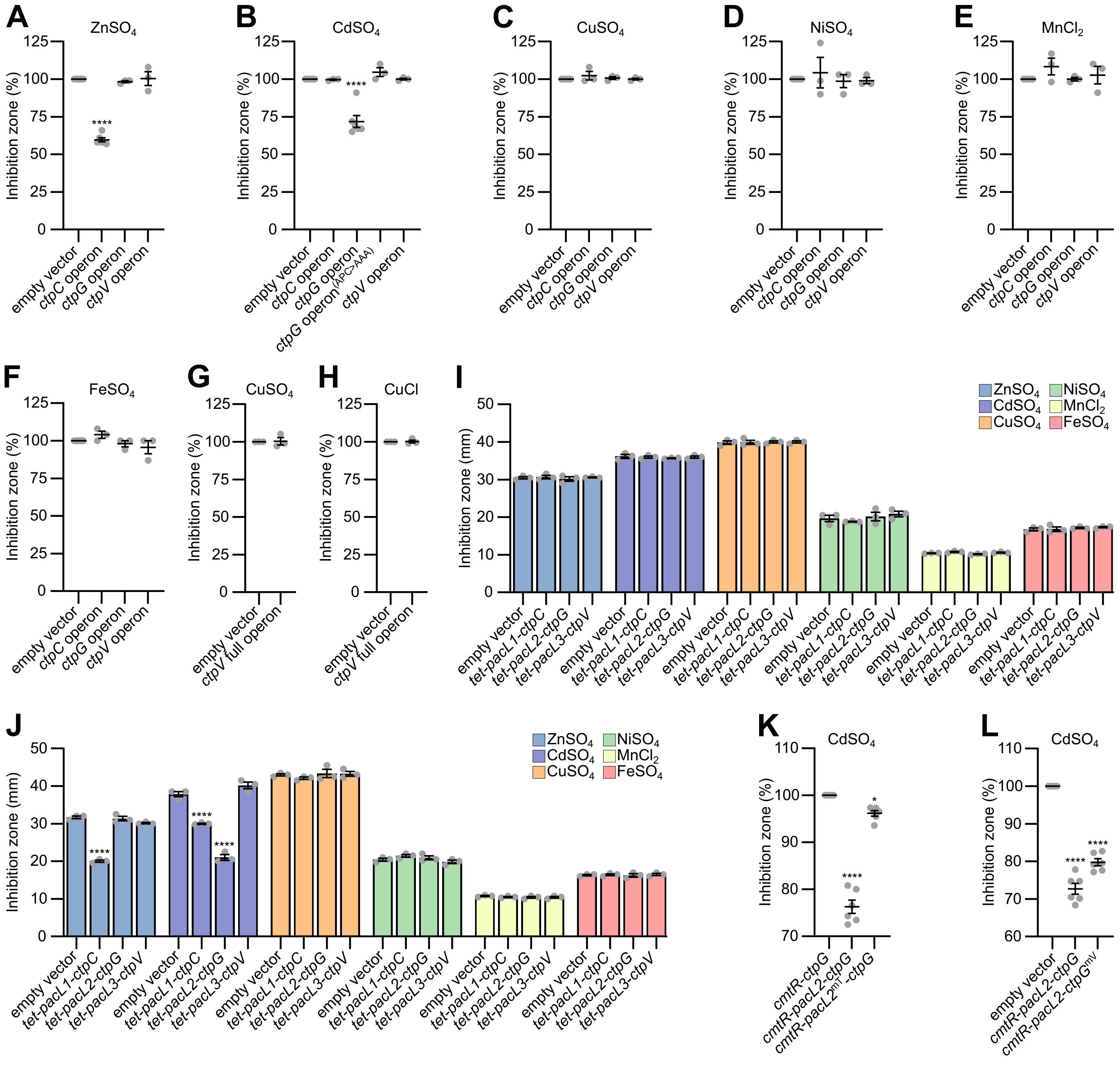

Figure EV1. Metal sensitivity of *M. smegmatis* strains expressing PacL/Ctp systems.

(A–L) Metal sensitivity of *M. smegmatis* strains carrying a genome-integrative vector expressing the indicated *M. tuberculosis* genes under the control of their native promoter (**A–H**, **K**, **L**) or an ATC-inducible promoter (**I**, -ATC; **J**, +ATC), assessed by disk diffusion assay. Inhibition zone diameters, normalized to the reference strain (empty vector (**A–J**, **L**)) or *cmtR-ctpG* (**K**), are shown as mean ± SEM from biological replicates, with individual values indicated by gray dots. For statistical analysis, one-way ANOVA with a Dunnett post-test were performed. Asterisks indicate statistically significant differences compared to the reference strain (**P* < 0.05; *****P* < 0.0001). Exact *p* values and biological replicate numbers presented in this figure are reported in Table EV6. ATC anhydrotetracycline, *tet* ATC-inducible promoter; *ctpC* operon: *pacL1-ctpC*; *ctpG* operon: *cmtR-pacL2-ctpG*; *ctpV* operon: *csoR-pacL3-ctpV*; *ctpV* full operon: *csoR-pacL3-ctpV-rv0970*; *pacL2*^{mT}, C-terminal translational fusion of PacL2 with mTurquoise; *ctpG*^{mV}, C-terminal translational fusion of CtpG with mVenus; (APC > AAA), amino acid substitutions within the conserved APC motif in the P-ATPase domain of CtpG. Source data are available online for this figure.

© The Author(s)

A

```
>Rv3269(PacL1)
MAIQVFLAKATTTVITGLAGVTAYEILKKAAAKAPLRQTAVSAAALGLRGTRKAEEAAESARLKVADVMA
EARERIGEESPTPAISDLHDHDH
>Rv1993c(PacL2)
MVTHELLVKAAGAVLTGLVGVSAYETLRKALGTAPIRRASVTVMEWGLRGTRRAEAAAESARLTVADVVA
EARGRIGEEAPLPAGARVDE
>Rv0968(PacL3)
MVWHGFLAKAVPTVVTGAVGVAAYEALRKMVVKAPLRAATVSVAAWGIRLAREAERKAGESAEQARLMFA
DVLAEASERAGEEVPPLAVAGSDDGHDH
```

B

```
PacL1    MAIQVFLAKATTTVITGLAGVTAYEILKKAAAKAPLRQTAVSAAALGLRG
PacL2    MVTHELLVKAAGAVLTGLVGVSAYETLRKALGTAPIRRASVTVMEWGLRG
PacL3    MVWHGFLAKAVPTVVTGAVGVAAYEALRKMVVKAPLRAATVSVAAWGIRL
         :. : :*.**. :*:** .**:*** *:*   .**:* ::*:.   *:*

PacL1    TRKAE----EAAESARLKVADVMAEARERIGEESPTPAISDLHD-HDH
PacL2    TRRAE----AAAESARLTVADVVAEARGRIGEEAPLPAGARVDE----
PacL3    AREAERKAGESAEQARLMFADVLAEASERAGEEVPPLAVAGSDDGHDH
         :*.**     :**.*** .***:***  * *** *  * :  .:
```

Figure EV2. High conservation of amino acid sequences among the three *M. tuberculosis* PacL proteins.

(**A**) Amino acid sequences of *M. tuberculosis* PacL1, PacL2, and PacL3 proteins. (**B**) Sequence alignment of *M. tuberculosis* PacL1, PacL2, and PacL3. Predicted transmembrane domains are shown in blue, AE repeats in red, and putative metal-binding motifs in purple.

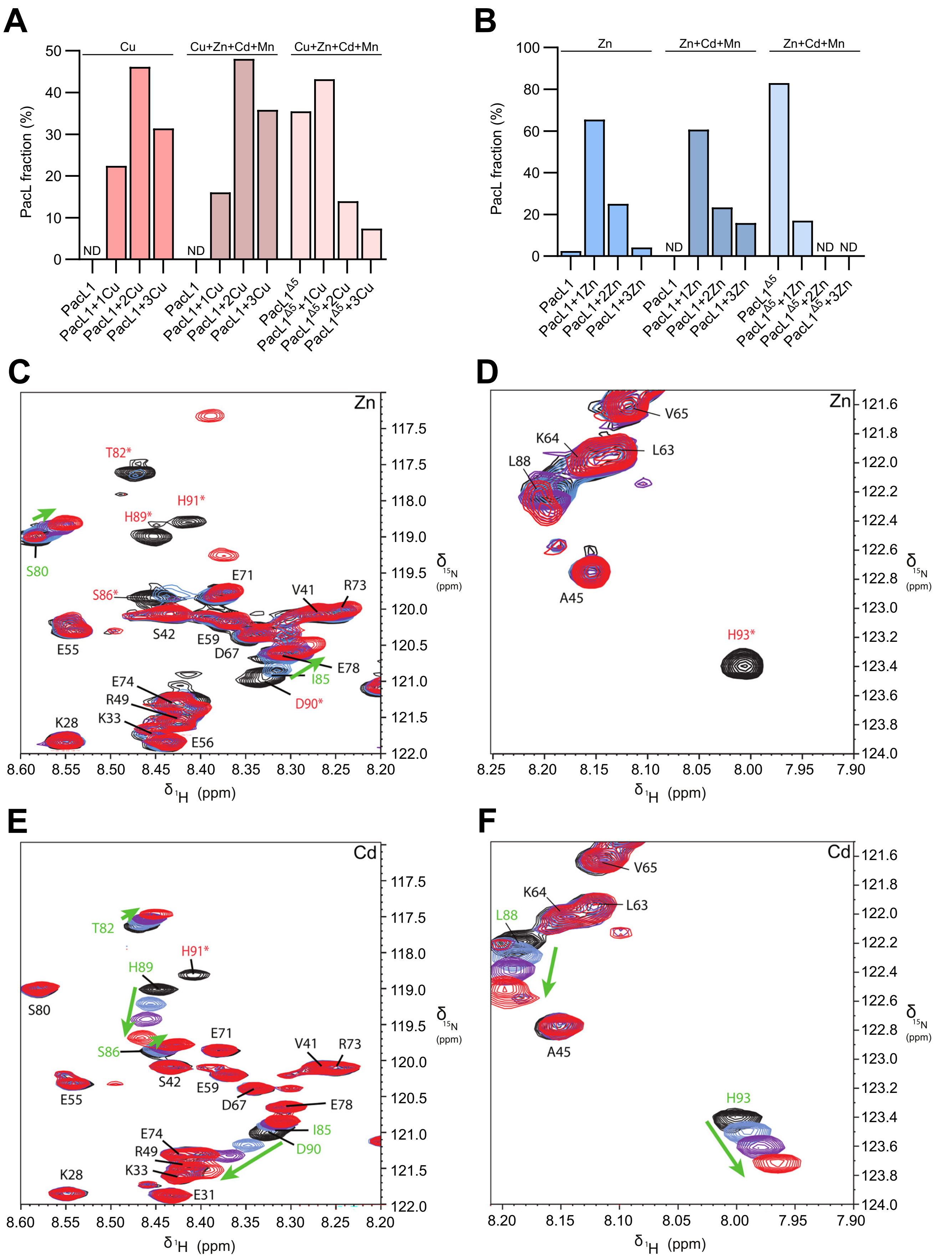
A
PacL fraction (%)
Cu
Cu+Zn+Cd+Mn
Cu+Zn+Cd+Mn
ND
ND
PacL1
PacL1+1Cu
PacL1+2Cu
PacL1+3Cu
PacL1Δ5
PacL1Δ5+1Cu
PacL1Δ5+2Cu
PacL1Δ5+3Cu
B
PacL fraction (%)
Zn
Zn+Cd+Mn
Zn+Cd+Mn
ND
ND ND
PacL1+1Zn
PacL1+2Zn
PacL1+3Zn
PacL1Δ5+1Zn
PacL1Δ5+2Zn
PacL1Δ5+3Zn
C
Zn
T82*
H91*
H89*
S80
E71
S86*
V41
R73
E55
S42
E59
D67
E78
I85
D90*
E74
R49
K33
K28
E56
δ 1H (ppm)
δ 15N (ppm)
D
Zn
V65
K64
L63
L88
A45
H93*
E
Cd
T82
H91*
H89
S80
E71
S86
V41
R73
E55
S42
E59
D67
E78
I85
D90
E74
R49
K33
K28
E31
F
Cd
V65
K64
L63
L88
A45
H93

 © The Author(s)

Figure EV3. PacL1 binds various metal ions mainly through its metal-binding motif, with distinct affinities and binding modes.

(**A**, **B**) Proportion of the indicated purified proteins bound to specific numbers of metal atoms, assessed by native mass spectrometry after incubation with either (**A**) 500 µM Zn/Cu/Cd/Mn or (**B**) 500 µM Zn/Cd/Mn. PacL1: soluble domain of PacL1; PacL1Δ5: soluble domain of PacL1 deleted of its C-terminal metal-binding motif. (**C–F**) ^{1}H-^{15}N HSQC overlay spectra of 90 µM ^{15}N-labeled SolPacL1 with 0 (black), 0.4 (light blue), 1.0 (purple), and 2.0 (red) equivalents of zinc, spectra (**C**, **D**), and cadmium, spectra (**E**, **F**). Peak assignments are directly annotated on the spectra with the following color code: black, no perturbation; green, perturbed residues in fast exchange; red with a star, perturbed residues in intermediate exchange. We observed a distinct binding mode for zinc, which induces significant peak broadening (intermediate chemical exchange) beyond the detection of residues involved in the metal-binding site (H89-H93), compared to cadmium, which remains mainly in fast exchange except for H91. Source data are available online for this figure.

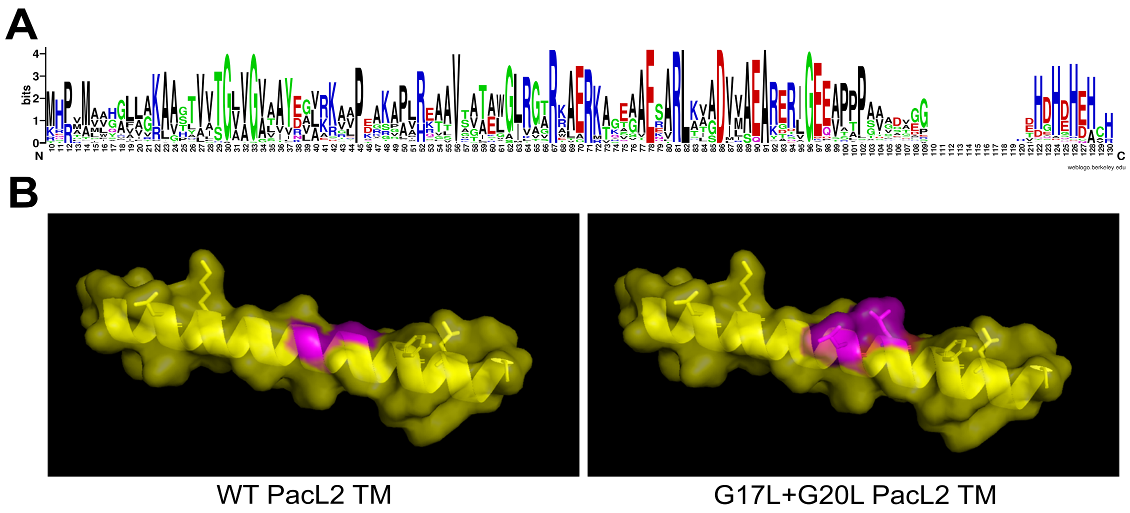

Figure EV4. Modeling of the conserved GXXG motif in PacL2.

(**A**) Sequence conservation logo of 120 bacterial PacL-like proteins (DUF1490-containing proteins). (**B**) Predicted structural models of the PacL2 transmembrane domain: (left) wild-type (WT) and (right) G17L + G20L mutant. Amino acids 17 and 20 are shown in magenta.

 © The Author(s)

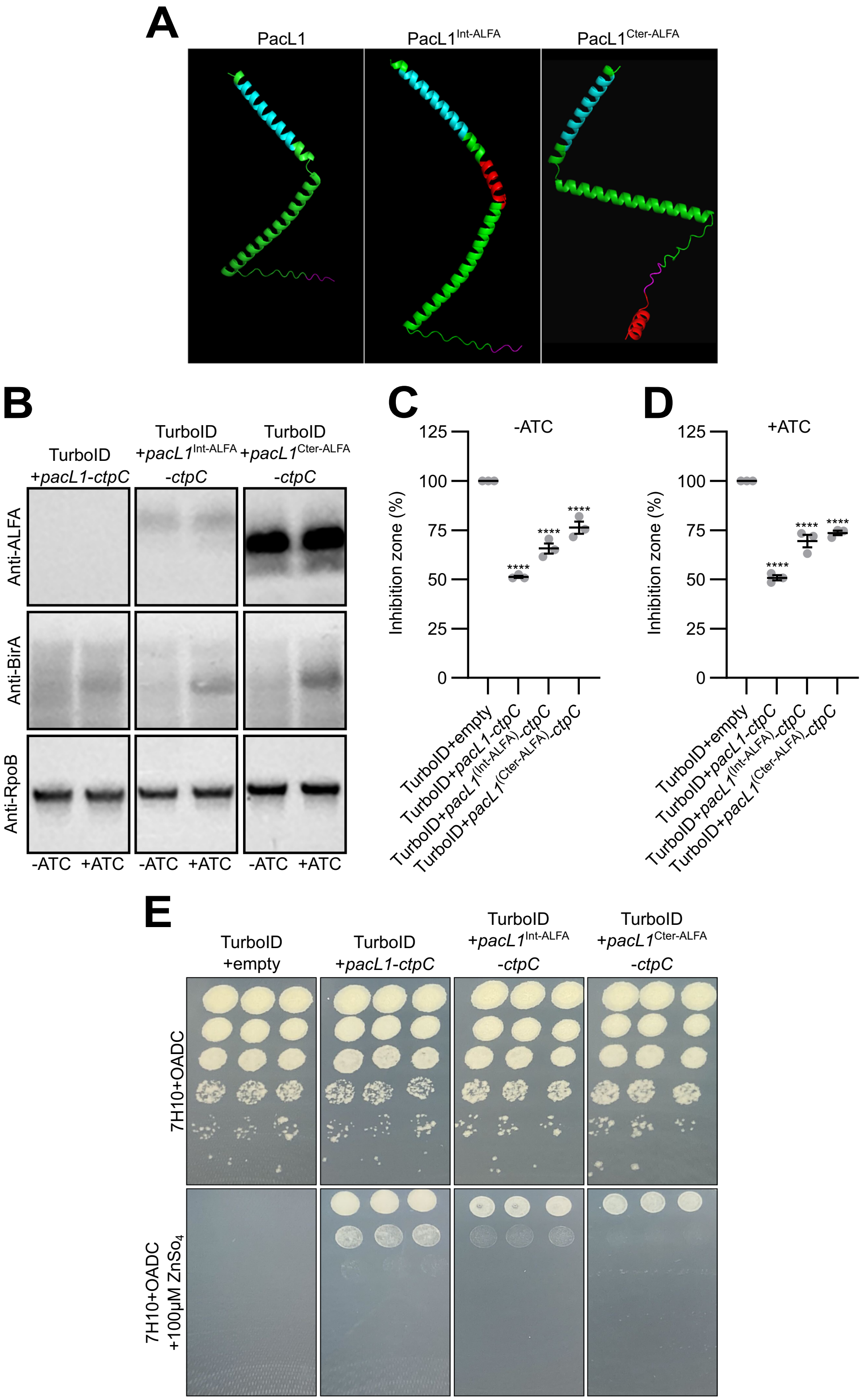
A
PacL1
PacL1 Int-ALFA
PacL1 Cter-ALFA
B
TurboID +pacL1-ctpC
TurboID +pacL1 Int-ALFA -ctpC
TurboID +pacL1 Cter-ALFA -ctpC
Anti-ALFA
Anti-BirA
Anti-RpoB
-ATC +ATC -ATC +ATC -ATC +ATC
C
-ATC
D
+ATC
Inhibition zone (%)
125
100
75
50
25
0
TurboID+empty
TurboID+pacL1-ctpC
TurboID+pacL1(Int-ALFA)-ctpC
TurboID+pacL1(Cter-ALFA)-ctpC
E
TurboID +empty
TurboID +pacL1-ctpC
TurboID +pacL1 Int-ALFA -ctpC
TurboID +pacL1 Cter-ALFA -ctpC
7H10+OADC
7H10+OADC +100µM ZnSo4

Figure EV5. Binding of TurboID to the PacL1^{ALFA} proteins does not abolish zinc tolerance.

(**A**) AlphaFold structural models of PacL1, PacL1 fused to a C-terminal ALFA tag (PacL1$^{Cter\text{-}ALFA}$), and PacL1 fused to an internal ALFA tag (PacL1$^{Int\text{-}ALFA}$), with the predicted transmembrane domain shown in blue and the ALFA epitope in red. (**B**) Anti-ALFA, anti-BirA, and anti-RpoB immunoblots of the indicated *M. tuberculosis* strains harboring the indicated plasmids, cultivated in the absence or presence of the anhydrotetracycline (ATC) inducer. (**C**, **D**) Zinc ($ZnSO_4$) sensitivity of *M. smegmatis* strains harboring the indicated plasmids, assessed by disk diffusion assay. The experiment was performed in (**C**) the absence or (**D**) the presence of the ATC inducer in the agar medium. Inhibition zone diameters normalized to the empty vector control are shown as mean ± SEM from biological replicates, with individual values indicated by gray dots. For statistical analysis, one-way ANOVA with a Dunnett post-test were performed. Asterisks indicate statistically significant differences compared to the TurboID+empty strain (****$P < 0.0001$). Exact *p* values and biological replicate numbers presented in this figure are reported in Table EV6. (**E**) Serial dilutions (5 µL) of *M. tuberculosis* cultures harboring the indicated plasmids, spotted on agar plates supplemented or not with 100 µM $ZnSO_4$. Three independent biological replicates are shown per condition. TurboID, replicative plasmid carrying the TurboID-nanobody fusion protein expressed under the control of an ATC-inducible (*tet*) promoter and detected with the anti-BirA antibody; *pacL1-ctpC*, *pacL1*$^{int\text{-}ALFA}$-*ctpC*, and *pacL1*$^{Cter\text{-}ALFA}$-*ctpC*, genome-integrative plasmids expressing WT PacL1, PacL1 with an internal ALFA tag, or PacL1 with a C-terminal ALFA tag, in operon with *ctpC* under the control of their native promoter. Source data are available online for this figure.

 © The Author(s)
